# Measuring the frequency and distribution of meiotic crossovers in homozygous barley inbred lines

**DOI:** 10.3389/fpls.2022.965217

**Published:** 2022-08-11

**Authors:** Miriam Schreiber, Yun-Yu Chen, Luke Ramsay, Robbie Waugh

**Affiliations:** ^1^Informational and Computational Sciences, James Hutton Institute, Dundee, United Kingdom; ^2^Cell and Molecular Sciences, James Hutton Institute, Dundee, United Kingdom; ^3^Division of Plant Sciences, School of Life Sciences, University of Dundee, Dundee, United Kingdom

**Keywords:** barley, recombination, homozygous lines, whole genome shotgun, ethyl methanesulfonate

## Abstract

We report a novel approach for establishing the number and position of CO events in individual homozygous inbred plants by combining low level EMS mutagenesis, speed breeding, whole genome shotgun sequencing and sliding window analysis of the induced molecular variant data. We demonstrate the approach by exploring CO frequency and distribution in self-fertilised progeny of the inbred barley cultivar Bowman and compare these observations to similar data obtained from a Bowman nearly isogenic line (BW230 *Hvmlh3)* containing a mutation in the DNA mismatch repair gene *HvMLH3.* We have previously shown that *Hvmlh3* decreases both plant fertility and recombination by ~50%. We compare our results to those from previously published traditional genetic analysis of F3 families derived from multiple F2 lines containing WT or mutant alleles of *HvMLH3,* revealing a high level of correspondence between analyses. We discuss possible applications of the approach in streamlining the assessment of recombination in plant meiosis research.

## Introduction

Meiotic recombination in plants is a key element in the drive to increase genetic diversity in plant breeding and genetics. During meiosis homologous chromosomes pair and DNA double stranded breaks (DSB), introduced throughout the genome by the topo-isomerase-related enzyme SPO11, define the possible spectrum of crossover (CO) positions within the genome (Higgins et al., 2014). However, the repair of only approximately 5% of these DSBs result in CO formation and the exchange of parental genetic materials along chromosomes. The remaining breaks are resolved as non-CO events (Osman et al., 2011). Successful crossing over is essential for proper chromosome segregation and shuffling of the genetic variation that is subsequently transmitted to the next generation (Altendorfer et al., 2020). However, in most large genome cereal crops such as barley, COs are highly skewed towards the telomeric ends of the chromosomes. The vast peri-centromeric regions which represent around 22% of individual chromosomes and contain 8% of the genes (Mascher et al., 2017) rarely, if ever recombine. Understanding and manipulating the frequency and distribution of successful COs has become a major objective in plant biology in general as this holds a potentially significant impact in practical crop improvement by releasing currently inaccessible genetic diversity, and hopefully leading to an increase in the rate of genetic gain (Able et al., 2009).

To establish the impact that either genetic perturbations (e.g., Colas et al., 2019) or imposed environmental stresses (e.g., Phillips et al., 2015) have on CO frequency and distribution, a host of approaches have been adopted over time by the plant research community. Cytological maps derived from the visualisation of COs (Sybenga, 1965; Anderson et al., 2003; Phillips et al., 2013), genetic maps assembled from molecular markers segregating in bi-parental populations (Colas et al., 2016), tetrad analysis (Copenhaver et al., 2000) and fluorescent protein-tagged loci expressed in pollen (Berchowitz and Copenhaver, 2008) or seed (Melamed-Bessudo et al., 2005) have proved informative in a variety of experimental scenarios. In addition, a range of genetic approaches for detecting polymorphisms between parental genotypes have been applied to assess the inheritance of parent-specific DNA polymorphisms in the male gametes (pollen) from F1 hybrid plants (e.g., Drouaud and Mezard, 2011; Khademian et al., 2013; Dreissig et al., 2015). Today, a combination of informational (e.g., genomic reference sequences) and technological (e.g., cell sorting, next generation sequencing) advances mean these methods have progressed to the point where whole genome short read or linked-read sequencing of high molecular weight DNA can provide an accurate picture of genome-wide CO frequency and distribution in populations of individual (Dreissig et al., 2017) or pooled gametes (Sun et al., 2019) or individual plants from populations segregating for chemically induced sequence variants (Blary et al., 2018; Lian et al., 2022).

However, all of these approaches rely first on the generation of hybrids between suitably diverse parental genotypes where divergent sequences at either the nucleotide or structural level (e.g., inversions or PAVs) may influence the observed outcomes. While they successfully catalogue and contextualise CO events at high resolution, they are constrained to chromosomal mosaics observed in the surviving genotypes of a population of progeny plants. For example, Dreissig et al. (2017) demonstrated that abundant segregation distortion observed in a doubled haploid barley population was completely absent in F1 pollen from the same parental lines allowing them to conclude that meiosis alone was not the main cause of the observed distortion.

Given we work on barley, a large genome (4.3Gb) inbreeding crop plant with a six-month generation time, we were motivated to explore a more cost and time-effective approach for monitoring productive recombination events. We wanted to take advantage of recently available reference genome information (Monat et al., 2019), new sequencing technologies and accelerated plant development (Ghosh et al., 2018; Watson et al., 2018). A major objective was to improve our efficiency at practically exploring crossing over in WT parental and derived mutant genotypes, or the impact of applying environmental treatments. We wanted to overcome the time consuming need to make sexual hybrids between diverse parents and avoid assessing crossing over in F3 families derived from multiple carefully chosen F2 individuals using current molecular marker technologies (e.g., SNP-arrays, GBS) as described previously (Colas et al., 2016). Here we report a new approach for establishing the number and position of CO events in individual homozygous inbred lines of barley.

## Materials and methods

### Mutagenesis and speed breeding conditions

Bowman and BW230 (*Hvmlh3*) seeds were treated with 25 mM Ethyl methanesulfonate EMS exactly as described previously (Caldwell et al., 2004). 35 M1 seeds for each genotype were sown and the M1 plants grown in a growth cabinet under speed breeding conditions with light conditions set at 2 h dark and 22 h light. The threshold for lighting was 200 μmol.m-2-1.s. Night temperature was set to 14°C and day temperature to 18°C. Plants were grown to maturity and M2 seeds harvested. Multiple seeds were sown and again grown under speed breeding conditions as described above. One individual for each genotype, that was indistinguishable from its respective parental line, was selected for whole genome shotgun sequencing as the M2 parent. These plants were grown to maturity and the M3 seeds harvested. 55 M3 seeds from each of the two M2 plants (Bowman and BW230), respectively, were germinated under normal glass house conditions of 16 h light at 20°C (nominal) and 8 h night at 15°C (nominal).

### DNA extraction and sequencing

Young, 14 days old, barley leaves were collected, frozen in liquid nitrogen, and stored at-80C. DNA extractions were carried out using Macherey-Nagel NucleoSpin Plant II Maxi kit (Germany) as per manufacturer’s instructions. Between 1.5 and 2 g of frozen leaf tissue was used per sample. DNA was sent on dry ice to Novogene for library preparation and whole genome shotgun sequencing. All samples were sequenced at Novogene using Illumina short reads 2x150bp on a NovaSeq 6,000. The sequencing depth was adjusted during the experiment. The two ‘parental’ M2 plants were sequenced to a depth of 15x coverage. Initially, 13 M3 plants derived from each M2 genotype were sequenced to a depth of 2x coverage. Subsequently, 12 different M3 plants from each M2 genotype were sequenced to a depth of 4x coverage.

### Bioinformatics analysis

#### Data availability statement

All scripts are available through the following GiHub repository: https://github.com/SchreiberM/Measuring-frequency-and-distribution-of-meiotic-cross-overs. The raw data has been deposited at the European Nucleotide Archive (ENA): PRJEB52593 (https://www.ebi.ac.uk/ena/browser/view/PRJEB52593).

#### Mapping and variant calling

Reads were mapped against Morex V2 (Monat et al., 2019) with default parameters of bwa mem (Li and Durbin, 2009; Li, 2013). Reads with more than 6 mismatches per read were removed, the remaining reads sorted, and duplicates marked using sambamba (Tarasov et al., 2015). Following the previous GATK3 best practice pipeline indel realignment was followed by two rounds of haplotype calling with a filtering and recalibration step in between (McKenna et al., 2010; DePristo et al., 2011). Freebayes was used for variant calling combining one M2 individual (either BW230.M2 or Bowman.M2) with an available whole genome shotgun dataset of Bowman wildtype (our unpublished data). Variant calling was done with a minimum coverage of 1, a minimum alternate fraction of 0, a minimum alternate count of 1 and a mapping quality of 30 (Garrison, 2012). Freebayes was also run with legacy mode (−-legacy-gls) and without any population priors. The two resulting variant files from BW230.M2 and Bowman.M2 were compared and all identical sites between the two files removed. This filtering step removed any background variation due to cultivar differences (Morex ‘reference’ versus Bowman ‘test’).

In a last filtering step, using more stringent filtering (minimum quality of 30, read depth of 3 for homozygous and read depth of 10 for heterozygous, an alternate count of at least 3, a total depth below 100) only high confidence SNPs were kept. In addition, we used very specific filtering steps reflecting the experimental setup. Firstly: we only kept SNPs that were heterozygous in the M2 but homozygous in the Bowman wildtype; secondly: we filtered for most likely EMS-induced mutations G - > A or C - > T; thirdly: we removed the previously defined introgressed region around *HvMLH3* on 5H and a short introgression at the end of 4H identified previously from genotypic analysis of BW230 and Bowman. For statistical analysis and identification of the effect of background mutations we included SNPs which had homozygous alternative alleles in the M2 but homozygous wildtype background in Bowman. SNPeff (Cingolani et al., 2012) and PROVEAN (Choi et al., 2012; Choi and Chan, 2015) were used to study the effect of the mutations. For SNPeff, the nonsense mutations introducing early stop codons were extracted. To predict the impact of amino acid changes from PROVEAN only sequences with at least 30 related sequences as support were considered and highlighted as deleterious if the cut-off score was below-4.1 which corresponds to a sensitivity prediction of above 90%.^1^

The heterozygous SNPs were further filtered for equal distribution and same number of SNPs with PLINK (Purcell et al., 2007). Overrepresented regions were thinned with --bp-space 10,000 and afterwards the whole dataset was reduced to 20,000 SNP markers for each dataset using –thin-count 20,000.

For mapping the M3 sequencing reads, the same parameters as described above were used. This time variant calling was carried out with Freebayes, using the previously identified and filtered heterozygous positions as an input file and only calling variants at those locations in the genome. For the individuals sequenced to a 2x coverage the SNPs were filtered for a read depth of 2 while for the individuals sequenced to a 4x coverage the SNPs were filtered for a read depth of 4 before processing further.

#### Switches from heterozygosity to homozygosity

We used a sliding window approach to identify clear switches from heterozygosity to homozygosity along each chromosome. To take account of the fact that each barley chromosome varies in length and distribution of genomic features (Data Figure 5 in Mascher et al., 2017) we first divided each chromosome into segments designed to take account of highly skewed patterns of recombination. We set the physical size of the sliding windows empirically; largest towards the centre of each chromosome (where recombination is virtually absent) and smallest towards the telomeric ends, providing greater resolution in highly recombinogenic regions. Double CO with less than four variants as support were not considered in the analysis. Full details of the approach are given in the GitHub repository under Sliding_window_approach.r. As a heterozygous call was more unlikely to be identified than a homozygous call as two alternative allele reads are necessary to score in comparison with only one, a weighted median was used for each window size with the weight of the heterozygous mutations set to 1.5 and the weight of the homozygous mutations to 1, choosing the lowest possible weight that removed background noise. For five samples (Bowman_EMS_M3_4, Bowman_EMS_M3_9, Bowman_EMS_M3_11, BW230_EMS_M3_6, BW230_EMS_M3_12) from the 2x sequence coverage data this weight was increased to 3 for the heterozygous mutations due to a higher background noise. The sliding window script was written in R (R Core Team, 2021). Full details and the script itself can be found in the above-mentioned GitHub repository.

## Results

**Figure 1:**
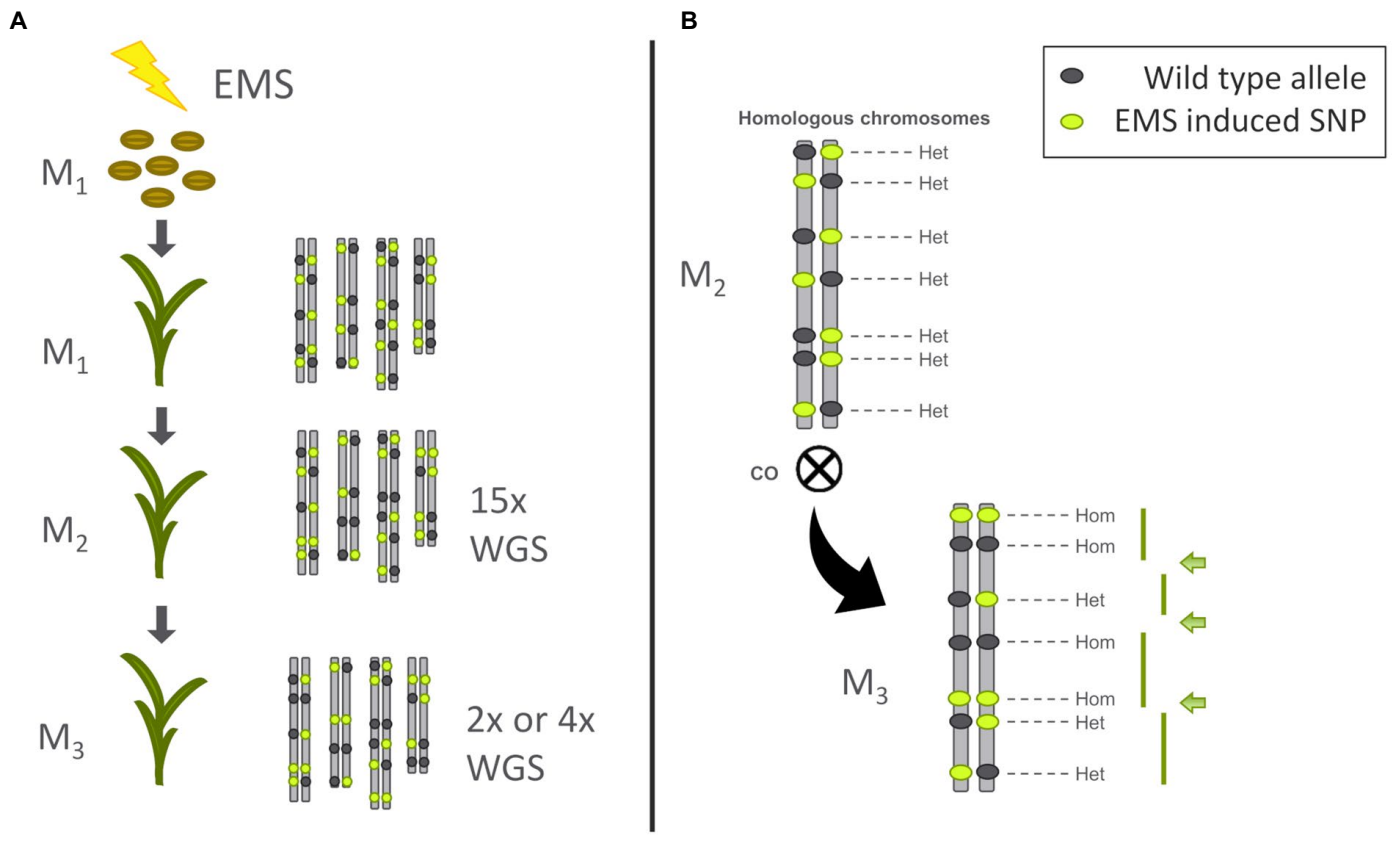
Experimental setup. **(A)** Overview from ethyl methanesulfonate (EMS) treatement to whole genome shotgun sequencing (WGS) **(B)** Counting of cross overs (CO) in the M3 plant.

We mutagenised M_0_ seeds of Bowman and BW230 (*Hvmlh3*), a Bowman near isogenic line carrying a mutation in the *HvMLH3* gene, with EMS. The resulting M_1_ plants were expected to be chimeric, with a different suite of mutations in different progenitor cell files. From the treated M_1_ seeds 71.43% germinated for Bowman and produced M_2_ seeds and 62.86% germinated for BW230 and produced M_2_ seeds. Barley has been previously determined to have an average Genetically Effective Cell Number (GECN) of six (i.e., the number of cells within the shoot meristem of the M_0_ seed that will ultimately contribute to the seed output of the M_1_ plants), which will lead to an expected segregation ratio for each EMS induced SNP of 21 WT: 2 heterozygous: 1 homozygous mutant (i.e., 23:1) in a population of M_2_ plants (Hodgdon et al., 1981). However, in each individual M_2_ plant, EMS induced SNPs should be present at a frequency of 1:2:1 (WT: heterozygous: mutant). Initially, multiple M_2_ seeds of each genotype were chosen and grown under speed breeding conditions. Leaf material was harvested from all M_2_ plants which were grown to maturity while monitoring plant health. All M_2_ plants were indistinguishable from their parental lines. One individual M_2_ plant was then chosen from each genotype (Bowman and BW230) and seeds harvested. DNA was extracted from the leaf material previously collected from these two plants and used for whole genome shotgun (WGS) sequencing to a depth of 15x coverage in order to construct a reference variant file and provide sequence context of SNPs introduced by the EMS treatment. Focusing only on the heterozygous SNPs in a single M_2_ plant then allowed us to determine changes in SNP phase (i.e., the switch from heterozygous to homozygous WT or Mutant allele) in the M_3_ families and therefore pinpoint CO positions (Figure 1).

WGS reads were mapped against the Morex V2 genome reference (Monat et al., 2019), duplicated reads removed and variant calling done by filtering for EMS introduced mutations of G to A or C to T (see Material and Methods). At this stage the phase of individual SNPs was unknown. We used SNPeff to predict the effect of the mutations and determine mutation frequency in the M_2_. For Bowman.M2 37,493 SNPs were identified with 98.9% of those being heterozygous. The mutation frequency for the homozygous mutations was 1 variant per 10 Mb, while the frequency for the heterozygous mutations was 1 every 116 kb. Of the homozygous mutations only one exon and two intron events were identified with the remaining variants being intergenic. Looking at the heterozygous mutations showed that only 1% of all heterozygous mutations were exonic and 1% intronic, while the remaining SNPs were intergenic. Of the exonic mutations 8 were predicted to cause a nonsense mutation (Table 1).

**Table 1:** Nonsense gene mutations in the M2 plants.

Gene	Position	Gene confidence class	Description	M2 plant
HORVU.MOREX.r2.1HG0037860	chr1H:320608594–320608836	LC	4-hydroxythreonine-4-phosphate dehydrogenase	Bowman
HORVU.MOREX.r2.2HG0114620	chr2H:203167494–203168468	LC	RNA-directed DNA polymerase (reverse transcriptase)-related family protein	Bowman
HORVU.MOREX.r2.2HG0135750	chr2H:456605166–456608394	HC	Potassium transporter	Bowman
HORVU.MOREX.r2.3HG0256650	chr3H:572579922–572584559	HC	ATP-dependent DNA helicase pif1	Bowman
HORVU.MOREX.r2.4HG0347090	chr4H:618733369–618736725	HC	Leucine-rich repeat receptor-like protein kinase family protein, putative	Bowman
HORVU.MOREX.r2.5HG0359800	chr5H:42617918–42618469	LC	Pericentriolar material 1 protein	Bowman
HORVU.MOREX.r2.5HG0388450	chr5H:362747281–362759083	HC	F-box protein	Bowman
HORVU.MOREX.r2.6HG0454880	chr6H:17765483–17770751	HC	SPOC domain/transcription elongation factor S-II, putative	Bowman
HORVU.MOREX.r2.1HG0043350	chr1H:369161477–369161977	LC	Transposon Ty3-I Gag-Pol polyprotein	BW230
HORVU.MOREX.r2.1HG0067510	chr1H:495315740–495316228	LC	Protein CHUP1, chloroplastic	BW230
HORVU.MOREX.r2.5HG0360590	chr5H:50824396–50825040	LC	Transposase	BW230
HORVU.MOREX.r2.6HG0503180	chr6H:469517357–469517830	HC	Trihelix transcription factor GT-3b	BW230
HORVU.MOREX.r2.7HG0602150	chr7H:564637316–564644792	HC	Type 2 DNA topoisomerase 6 subunit B-like	BW230

Gene confidence is split into low confidence (LC) and high confidence (HC) classes. All identified nonsense mutations were heterozygous.

For BW230.M2 25,303 SNPs were identified with 97.1% of those being heterozygous. There were small differences between BW230.M2 and Bowman.M2, with homozygous mutations identified at a frequency of 1 variant per 5 Mb and heterozygous mutations at a frequency of 1 variant every 175 kb. Of the homozygous mutations 7 were in exons and 6 in introns while the remaining were intergenic. The distribution of the heterozygous variants across the genome was almost identical to Bowman.M2 with 1.1% of the variants located in the exons and another 1.1% located in the introns. The remaining SNPs were intergenic. Of the exonic SNPs, 5 were predicted as nonsense mutations (Table 1).

PROVEAN was used to predict the impact of amino acid changes on the protein function. A total of 392 genes were checked across Bowman.M2 and BW230.M2. For 55 of those, which were all heterozygous in the M2, potentially deleterious amino acid changes were predicted (Supplementary Table 1).

**Figure 2:**
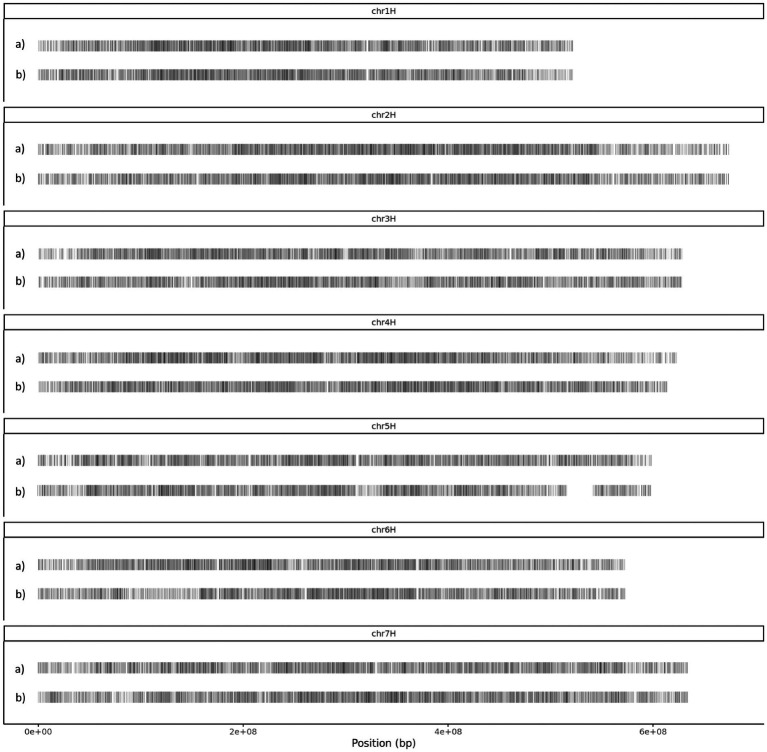
SNP distribution across the seven barley chromosomes, a) Bowman and b) BW230. The introgressed regions have been removed and can be seen as gaps on chromosome 5H and at the end of chromosome 4H.

As there was a difference in total number of heterozygous SNPs between Bowman.M2 and BW230.M2 the dataset was first thinned by using Plink to maintain 1 SNP every 10 kb and achieve a more equal distribution along the genome. We further reduced the whole dataset to 20,000 heterozygous SNPs of similar depth and distribution between the two genotypes. The distribution of the SNPs across the genome is shown in Figure 2. As the *HvMLH3* gene is located on the long arm of chromosome 5H as part of an introgression that carries a spontaneous *Hvmlh3* mutation in the donor Betzes, this region and another introgressed region at the end of chromosome 4H (identified previously by genotyping with the barley 50 K SNP array) were not considered for the final variant dataset in BW230.

**Figure 3:**
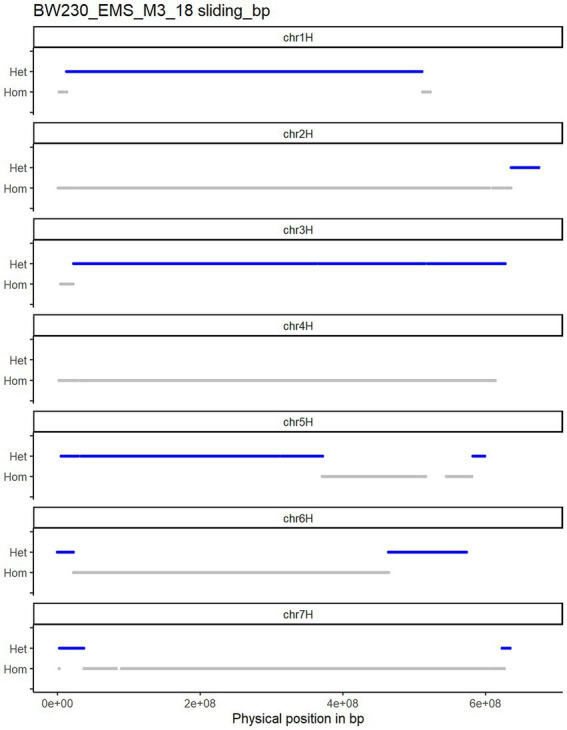
Plotted variant calling for Bowman_EMS_M3_18 plant after using the sliding window approach. COs can be counted at the positions where a switch from heterozygous (blue) to homozygous (grey) occurs.

We grew 50 seeds from each M_2_ genotype (i.e., the M_3_ generation) and initially sequenced 13 from each to a depth of 2×. After mapping and variant calling the SNPs were filtered for those already identified in the M_2_. In a first approach we plotted the unfiltered SNP zygosity along the chromosomes (raw plots in Supplementary material 1). The resulting plots were noisy, and clear switches between homozygous and heterozygous stretches were difficult to determine (as described in Figure 1). This is due to a combination of low sequencing depth, mismapping and sequencing errors. To be able to call a heterozygous SNP at least two reads are necessary one with the reference allele and one with the alternative allele. With a low sequencing read depth of 2 there will be many cases with just one read or no read at some positions. Nevertheless, the raw plots already showed regions with more dense heterozygous SNPs in different parts of the chromosome. Therefore, we adopted a sliding window approach to increase the robustness of the genotype calls. Taking the structure of the barley genome into account, we changed the size of the sliding window depending on the position along the chromosome. As most recombination events occur in the telomeric ends of the chromosomes we used a smaller physical window size towards the telomeric ends (average 14.3 Mbp) while using a larger window size in the pericentromeric and centromeric part (average 42.0 Mbp) of the genome (an explanation of how we established window sizes is given in the methods and the script provided in the GitHub repository). Importantly, only one mapped read is sufficient to be called as homozygous while at least two mapped reads containing alternative alleles are needed for a heterozygous call. Thus, noise is more likely to come from homozygous wild type calls. We therefore used a weighted median call across the sliding window, weighing the heterozygous calls as 1.5 in comparison with the homozygous calls as 1. This cleared up the phasing of the SNPs and allowed the straightforward calling of CO events (Figure 3). We then sequenced another 12 plants for each genotype to a depth of 4x to compare if increasing the sequencing depth led to different or improved results.

**Figure 4:**
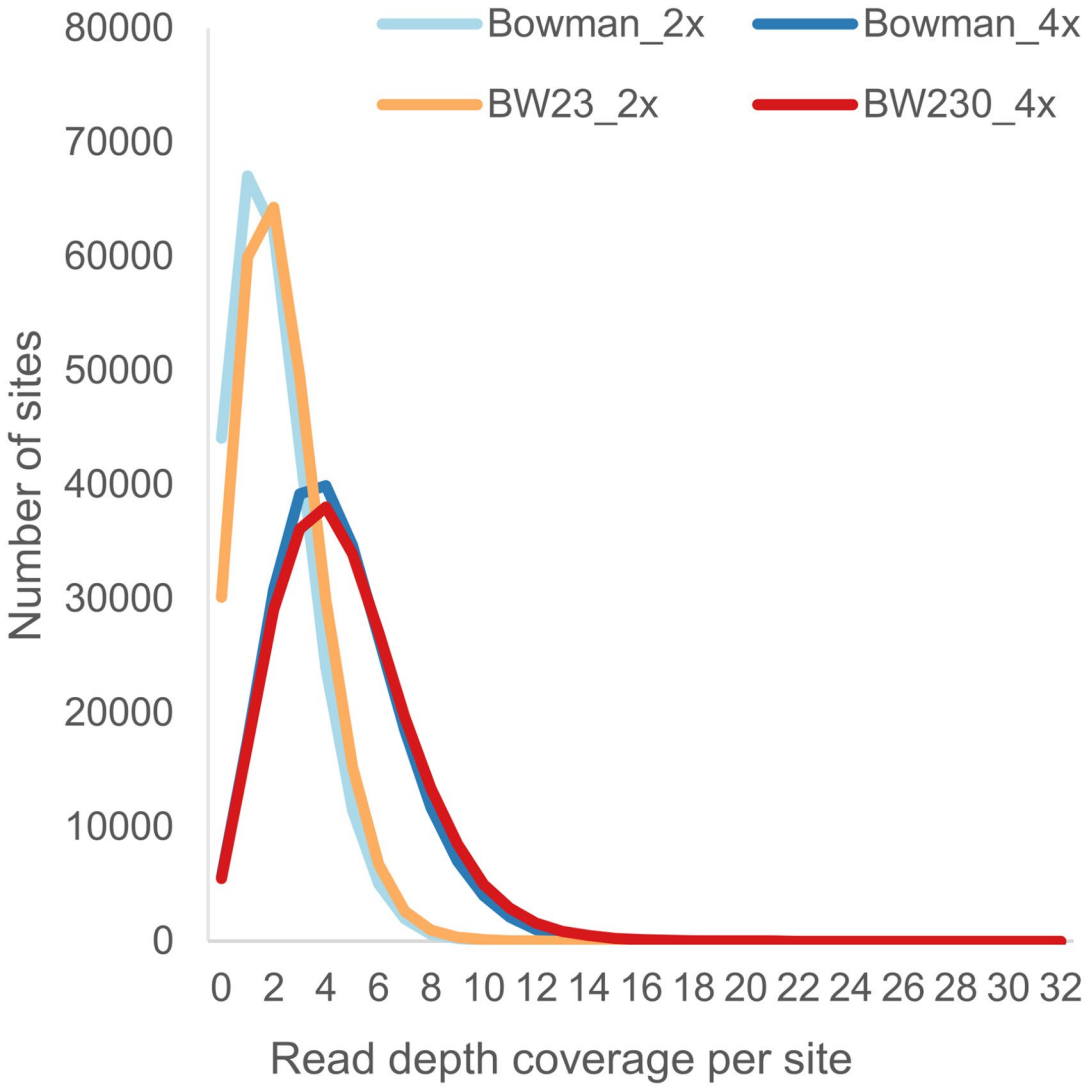
Read depth of the M3 individuals across the pre-called sites from the M2 individuals.

**Figure 5:**
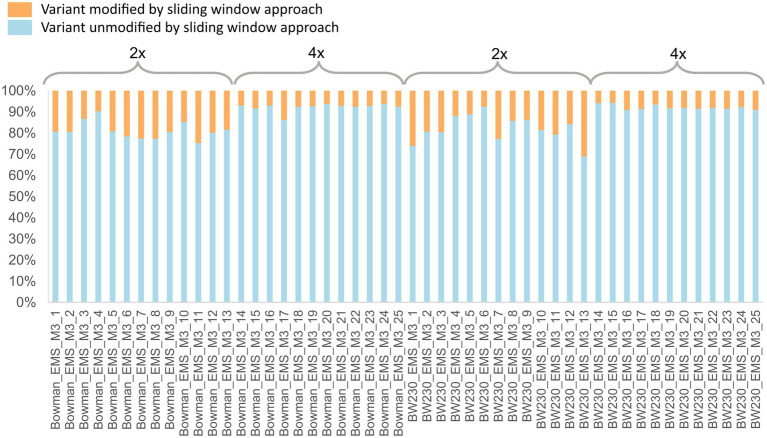
Percentage of variant sites which remained unmodified (blue) by the sliding window approach and variants with an adjusted zygosity call (orange) by the sliding window approach. Individuals 1–13 were sequenced to a depth of 2x coverage, while individuals 14–25 were sequenced to a 4× coverage read depth.

Quality control of the called SNP sites showed that the requested and expected read depth did correspond with the actual read depth (Figure 4). No obvious differences could be observed when comparing the raw data plots between the two different coverages (Supplementary material 1). To determine if there was a qualitative difference between the two coverages, we compared SNP zygosity before and after adjusting based on the sliding window approach. Between 5.7 to 31.2% of the SNP calls were changed from heterozygous to homozygous or vice versa by the sliding window approach with on average 18% of the positions adjusted in the samples with 2x coverage while only 8% of the positions needed to be corrected using the 4x coverage (Figure 5).

**Figure 6:**
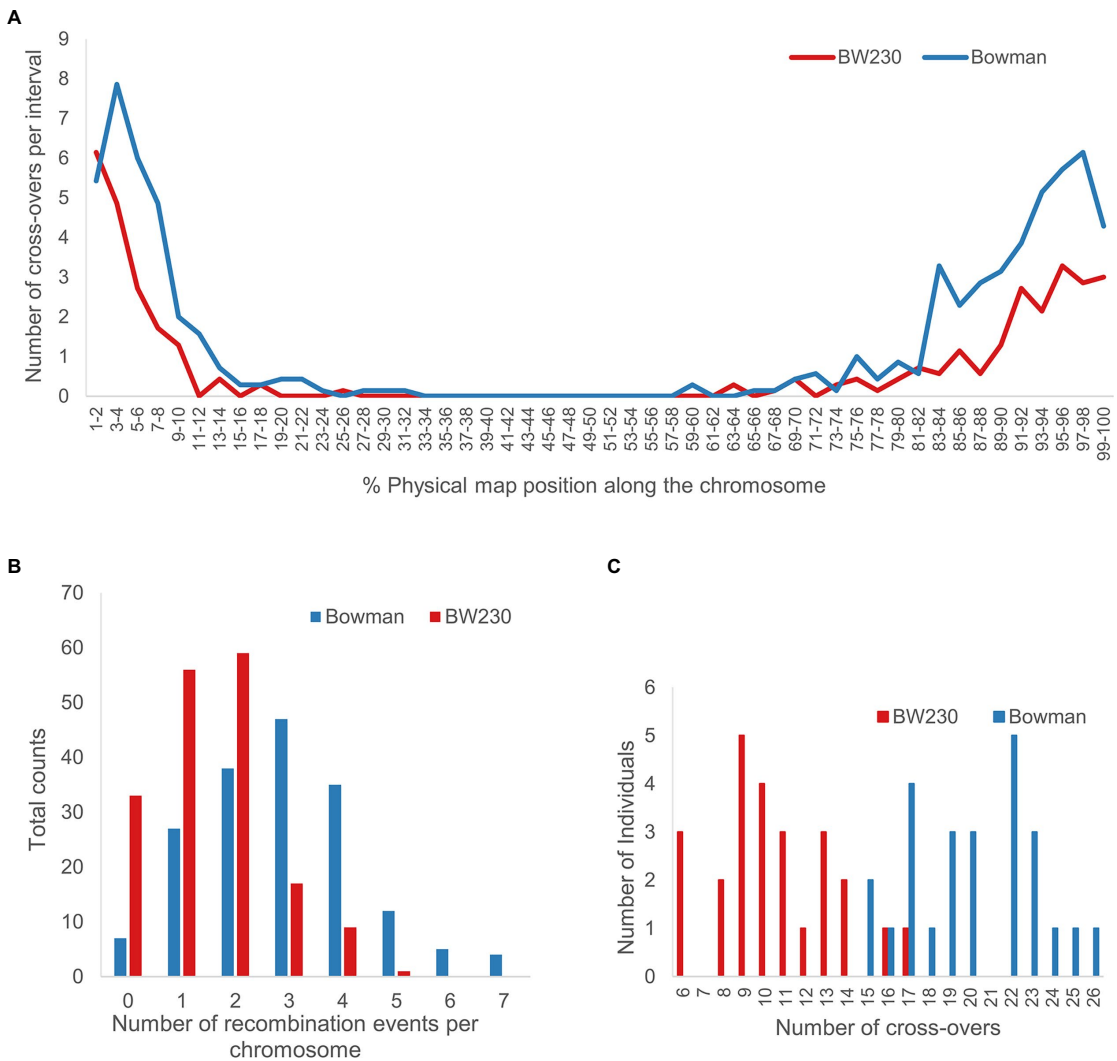
Number of CO events for the 175 chromosomes of 25 Bowman M3 individuals and 25 BW230 M3 individuals, respectively. **(A)** Average number of CO across all chromosomes per 2% intervals along the physical map. **(B)** Total counts of CO per individual chromosome. **(C)** Total number of CO per individual plant. Average number of CO for Bowman wildtype (blue) was 20.16 ± 3.1 and a median of 20. Average number of CO for BW230 was 10.64 ± 2.9 and a median of 10.

In total 25 individuals for Bowman.M3 and 25 individuals for BW230.M3 were analysed as described above and shown in Figure 3 (all remaining plots with the CO positions highlighted in Supplementary material 1). Combining all results and calculating the number of CO for each individual plant led to an average 20.16 ± 3.1 COs in Bowman.M3 and 10.64 ± 2.9 CO for BW230.M3 (Figure 6C). Plotting the average number of CO along the physical chromosome map split into 2 per cent intervals showed a higher number of COs towards the telomeric ends and a pericentromeric region almost completely depleted of COs (Figure 6A). For some of the individual chromosomes (Supplementary material 2) the first 2% of the chromosomes had a higher number of CO events in the BW230 in comparison with the wildtype, but for the remaining 98% the wildtype showed a higher number of CO.

Of the 175 WT chromosomes analysed, 27 revealed one CO event, 38 two, 47 three, 35 four, 12 five, 5 six and 4 seven CO events, numbers reflecting recombination in both male and female gametes. This compared to 56 with one CO event, 59 with two, 17 with three, 9 with four and 1 with five CO events in the 175 Hv*mlh3* mutant chromosomes analysed. 7 of the wildtype chromosomes and 33 of the mutant chromosomes had no CO events (Figure 6B).

## Discussion

In an inbreeding crop plant such as barley, the routine approach to compare recombination rate and distribution in WT and meiotic mutants or disruptive transgenics has been to construct and compare genetic maps using high density genetic marker analysis (Colas et al., 2016, 2019; Arrieta et al., 2021a). The approach generally uses the F3 progeny from multiple F2 individuals, that are homozygous for either WT or mutant alleles at the gene of interest, which are derived from a heterozygous F1 hybrid constructed from a cross between genetically diverse parents. The use of an F1 hybrid is required to introduce the genome-wide polymorphisms that enable genetic analysis. F3 families from multiple F2 individuals are required to cover all polymorphic regions of the parental genomes, and genome-wide segregation patterns can thus be monitored within the progeny. Any regions that are identical by descent are excluded from the analysis. A comparison of the resulting genetic maps reveals the extent to which the mutant allele has either increased or decreased recombination and/or altered the distribution of recombination events. Being monitored in the living progeny from a sexual cross, all CO events observed in the populations are derived from viable gametes that have been subject to gametic or zygotic selection. While informative, this genetic approach is relatively slow, requires at least two sexual generations, and is expensive due to the need for multiple rounds of genotypic analysis. Recombination in heterozygous F1’s may also be influenced by biological issues such as sequence diversity and segregation distortion in bi-parental populations from diverse origins (e.g., Salome et al., 2012). Furthermore, investigations in similar or identical inbred lines, for example between a NIL and its recurrent parent, or a treatment vs. control, are not suited to this traditional form of analysis.

Here we developed and tested an approach that maintains the biological constraints of observing recombination in viable gametes while also allowing analysis of recombination in essentially homozygous inbred lines. We assumed that the resulting data would provide more biologically accurate measures of recombination than those obtained by either high throughput sequence variation analysis of collections of individual gametic cells (Khademian et al., 2013; Dreissig et al., 2017; Sun et al., 2019), scoring recombination in specific genetic/physical intervals through pollen or seed-based assays (Melamed-Bessudo et al., 2005; Berchowitz and Copenhaver, 2008) or even the analysis of multiple F3 families derived from appropriate F2’s (as described above). We demonstrate the approach and test our assumptions on small populations of an inbred barley genotype cv. Bowman and a derived NIL (BW230) that contains a mutation in the DNA mismatch repair protein HvMLH3, which we have previously shown through traditional genetic analysis to reduce genome-wide recombination by approximately 50% in a population of viable *mlh3/mlh3* progeny (Colas et al., 2016). This allowed a direct comparison of recombination distribution and frequency observed independently in a meiotic mutant and its isogenic WT parent, and also a comparison with recombination measured in the F3 progeny from a traditional intercross obtained from using the *HvMLH3* mutant (*Hvmlh3/mlh3)* and a WT (*Mlh3/Mlh3*) as parents.

The barley genome is relatively large at ~4.3Gb and has been previously characterised both cytogenetically and using molecular markers as having extensive non-recombining pericentromeric regions covering roughly half of each chromosome (Mascher et al., 2017). In addition, measures of haplotype diversity reveal that these pericentromeric regions can be highly divergent. We were therefore interested to establish whether the observed lack of recombination in these regions was possibly the result of this extensive sequence divergence. We did not observe an increase in recombination in the pericentromeric regions despite them being identical at the sequence level.

We treated homozygous WT and isogenic *mlh3/mlh3* mutant seed with a low level of EMS (25 mM), which we had previously established induced few morphological or developmental phenotypes (Caldwell et al., 2004; Schreiber et al., 2019). Then, 15X coverage short read Illumina Sequencing of individual M2 seedlings followed by sequencing individual M3 progeny to 2X or 4X allowed us to catalogue the majority the induced polymorphisms. Only around 1% of the induced mutations were found in coding sequences and > 97% of the mutations were heterozygous. We found none in processes that were obviously related to gametogenesis, meiosis or recombination, a finding reflected in the chosen M2 plants which were phenotypically indistinguishable from their parental lines.

While the raw SNP data was noisy in terms of identifying true recombination events, incorporating a sliding window in the analysis of the low coverage data improved robustness and in combination with 2X coverage of individual progeny was sufficient to reliably score CO events. Recombination analysis then relies simply on counting the number of switches from heterozygosity to homozygosity in the M3 progeny. We then plotted their physical distribution against the barley genome reference (Mascher et al., 2017; Monat et al., 2019). Combining results from all individuals, the M3 revealed an average of 20.16 ± 3.1 CO in Bowman and 10.64 ± 2.9 CO in BW230 M3 confirming the negative impact of *mlh3/mlh3* on CO as shown previously (Colas et al., 2016). Despite the numbers being relatively small (i.e., 175 chromosomes in each case) they are close to the mean cytological chiasma counts of 18.4 ± 1.3 for WT and 9.2 ± 2.1 for *Hvmlh3* (Colas et al., 2016) and the CO estimates derived from genetic mapping of 19.7 for WT and 7.1 for *Hvmlh3* (Arrieta et al., 2021a) as well as being congruent with the mean CO numbers of 21.8 in WT populations estimated previously from genetic maps (Close et al., 2009) suggesting few issues associated with comparing carefully conducted cytological analyses (despite the difficulties of resolution), and with traditional genetic analyses. While the number of CO is reduced in *Hvmlh3* in comparison with WT the distribution along the chromosome does not seem to be affected (Figure 6A) which is again comparable to what has been observed by Colas et al. (2016) and Arrieta et al. (2021a) and to expectations given the role of MLH3 (Colas et al., 2016).

Making direct comparisons between our data and that from tetrad, fluorescent reporter or pollen-based sequencing assays are more problematic (Dreissig et al., 2015, 2017). Comparison with the latter, as it was also conducted in barley, is probably most appropriate. This approach involved low coverage (ave. 0.1X) short read sequencing of 40 quasi-random PCR-amplified DNA’s from individual flow-sorted pollen nuclei from a heterozygous F1 plant. The resulting data was effective in summarising the recombination landscape, exploring the origins of segregation distortion in doubled haploid populations and investigating CO interference in the barley genome (albeit only for male meiosis). Technically, however, it requires specialist equipment and expertise for flow sorting pollen nuclei originating from F1 hybrid plants, access to a reference genome sequence of at least one of the parental genotypes (cv. Morex, Monat et al., 2019) and a database of linearly ordered SNPs, in this case previously ascertained in a genetic population derived from the same parental genotypes that were used to generate the F1 pollen (ie. *cv*. Morex and *cv*. Barke, Mascher et al., 2013). In the approach we describe here, all heterozygous SNPs were ascertained directly in *cv*. Bowman. As in Dreissig et al. (2017) they were physically ordered along each chromosome according to the same reference barley genome (*cv.* Morex). Given the relatively frequent occurrence of small inversions or rearrangements in the barley genome this could potentially result in an inflated assignment of physically close double recombinants, but this was not explicitly tested here. A reference standard genome assembly of *cv.* Bowman will be required to explore this further.

An obvious question about the approach we describe here is ‘when would it be attractive to use?’ We give two examples. First, and continuing on the theme of using barley, large collections of phenotypically characterised homozygous ‘semi-fertile’ (or ‘desynaptic’) mutants are stored in international genebanks or local collections (e.g., we hold a collection of around 300 semi-fertile barley mutants in two different cultivated genetic backgrounds). As ‘semi-fertility’ is often associated with mutations in genes affecting meiosis, an obvious question for us is whether CO frequency or distribution is increased or decreased in some of these lines, and whether this will have practical value. Second, we know that different stresses or treatments (e.g., temperature, nutrients) can affect recombination and exploring these effects at the genetic level is a routine question (Arrieta et al., 2021b). While this could be done in both cases by the traditional approach of analysing the inheritance of genetic markers in contrasting F3 families derived from F1 hybrid seed, we suggest that our approach can streamline this analysis while avoiding biological issues resulting from genotypic effects (e.g., genotype dependent segregation distortion, levels of variation, identity by descent, lethal alleles, etc.). It can also be time and resource efficient as only few plants are grown to maturity. We start with a small number of seed and mutation induction is completed in just over a day. By growing primary M1 plants under speed breeding conditions, M2 seed can be available within 60 days. Growth of a small number of individual M2, short read sequencing, and analysing the data can be done while the M2 grow and set M3 seed. DNA from germinated M3 seed can be extracted in the lab 4–6 days after imbibition and molecular analysis (DNA isolation, library construction and sequencing followed by bioinformatic analysis) conducted as appropriate. We used largely standard molecular biology approaches throughout including commercial kits for library preparation and sequencing, and for the 4.3Gb barley genome we found the process both faster and logistically simpler. However, using standard commercial kits and approaches it was not significantly cheaper than the traditional genetic approach we have used in the past. We nevertheless we expect that bespoke sequencing library preparation protocols avoiding the use of commercial kits, higher multiplexing, instruments with higher sequencing throughput and use on plants with smaller genomes will all contribute to driving down these costs. For inbred diploid barley, comparing 2x and 4x sequencing depths, 2x coverage was sufficient when coupled with adjustments to the sliding window analysis.

## Conclusion

We show that introducing a low level of polymorphisms into the genomes of diploid homozygous inbred barley lines using EMS combined with low coverage whole genome shotgun sequencing and sliding window data analysis of induced variants in M3 populations can be used to determine CO numbers and recombination rate in essentially inbred lines. While we analysed a relatively small number of chromosomes (i.e., 175) in both WT and meiotic mutant plants, this was sufficient to demonstrate CO frequency and distribution and the impact of a homozygous mutation in *HvMLH3*. We found that the outputs of our analyses were directly comparable to those obtained using routine genetic analysis of segregating F3 families. The major advantage is that we achieved this both quickly and logistically simply, and in the absence of potentially complicating biological (e.g., doubled haploidy, pollen sequencing) or genotypic (e.g., deleterious allelic combinations) effects that in certain scenarios could influence our overall conclusions.

## Data availability statement

The datasets presented in this study can be found in online repositories. The names of the repository/repositories and accession number(s) can be found in the article.

## Author contributions

MS and Y-YC conducted the experimental and bioinformatics analysis. RW and LR designed the experimental setup. MS, LR, and RW wrote the manuscript. All authors contributed to the article and approved the submitted version.

## Funding

MS and Y-YC were supported by the ERC Advanced Grant SHUFFLE (Project ID: 669182) to RW. MS, LR, and RW were in addition funded from the Scottish Governments Rural and Environment Science and Analytical Services Division Theme 2 Work Program 2.1.

## Conflict of interest

The authors declare that the research was conducted in the absence of any commercial or financial relationships that could be construed as a potential conflict of interest.

## Publisher’s note

All claims expressed in this article are solely those of the authors and do not necessarily represent those of their affiliated organizations, or those of the publisher, the editors and the reviewers. Any product that may be evaluated in this article, or claim that may be made by its manufacturer, is not guaranteed or endorsed by the publisher.

## Footnotes

^1^ http://provean.jcvi.org/about.php
