# Supplementary material for: Measuring the frequency and distribution of meiotic crossovers in homozygous barley inbred lines
Source: Front Plant Sci. 2022 Aug 11;13:965217. doi: 10.3389/fpls.2022.965217 (PMC9403744; doi:10.3389/fpls.2022.965217)
Supplement: Supplementary file 1 [file Presentation_1.pptx]

## Slide 1
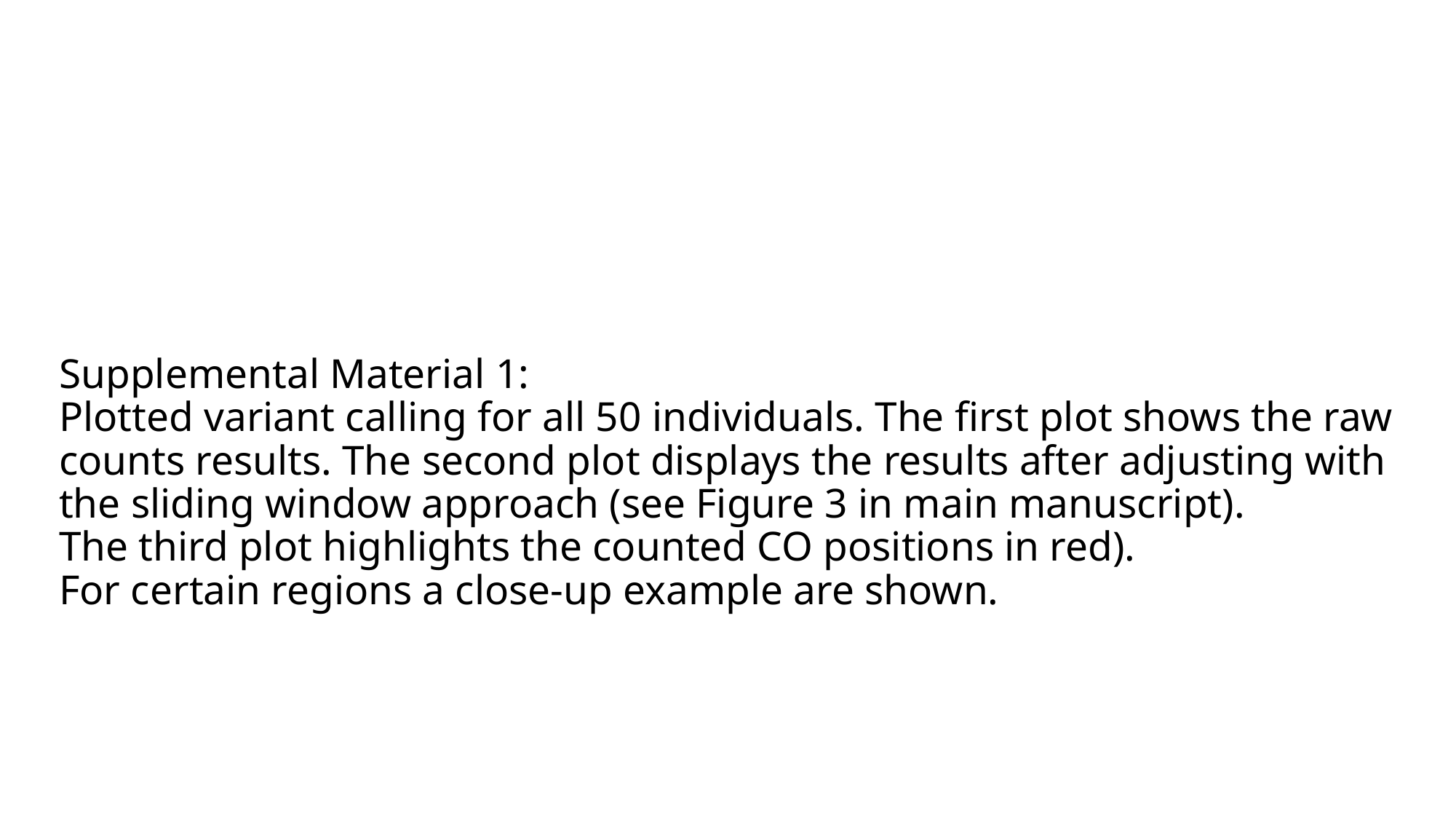

# Supplemental Material 1: Plotted variant calling for all 50 individuals. The first plot shows the raw counts results. The second plot displays the results after adjusting with the sliding window approach (see Figure 3 in main manuscript). The third plot highlights the counted CO positions in red). For certain regions a close-up example are shown.

## Slide 2
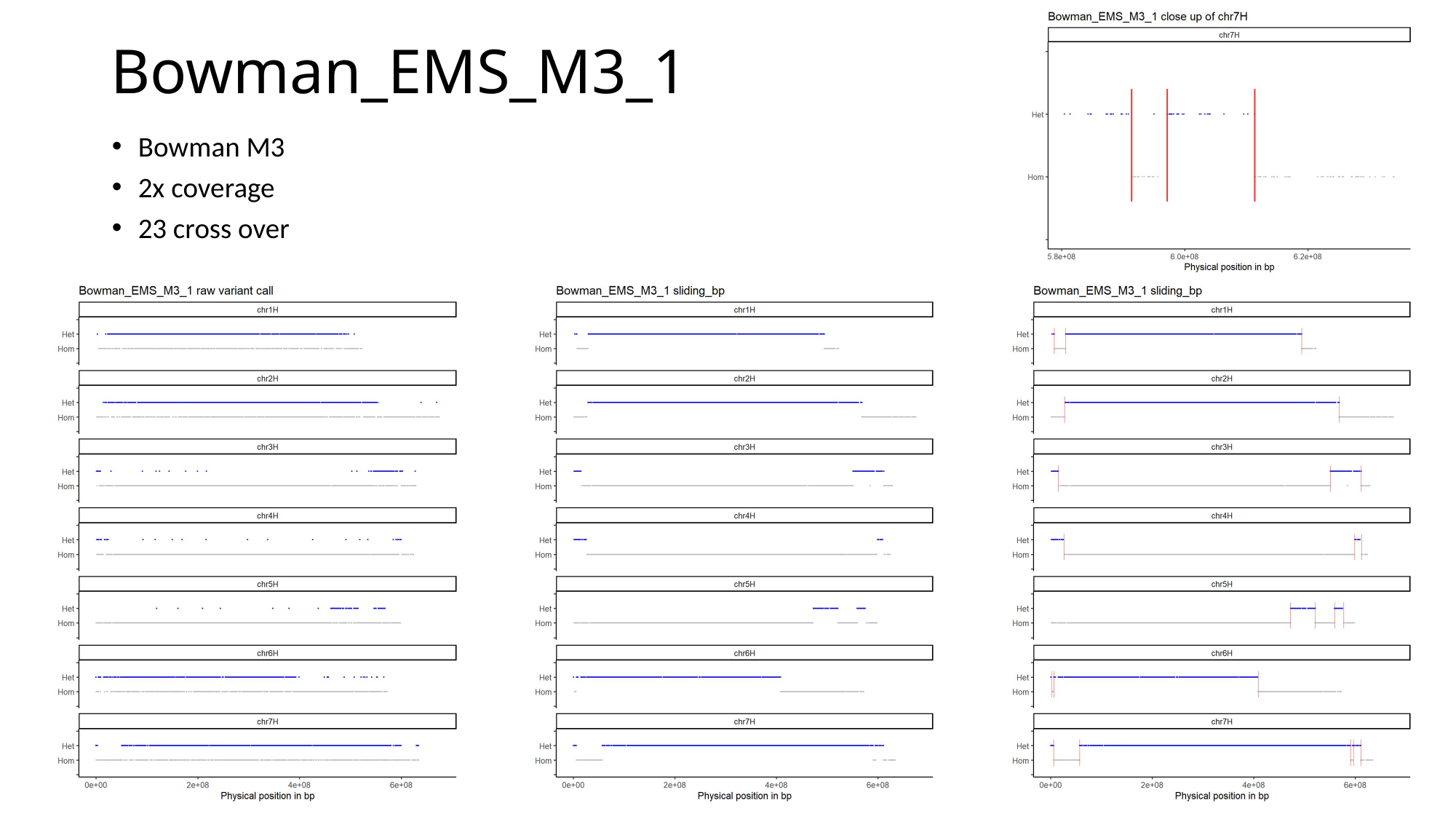

Bowman_EMS_M3_1
Bowman M3
2x coverage
23 cross over

## Slide 3
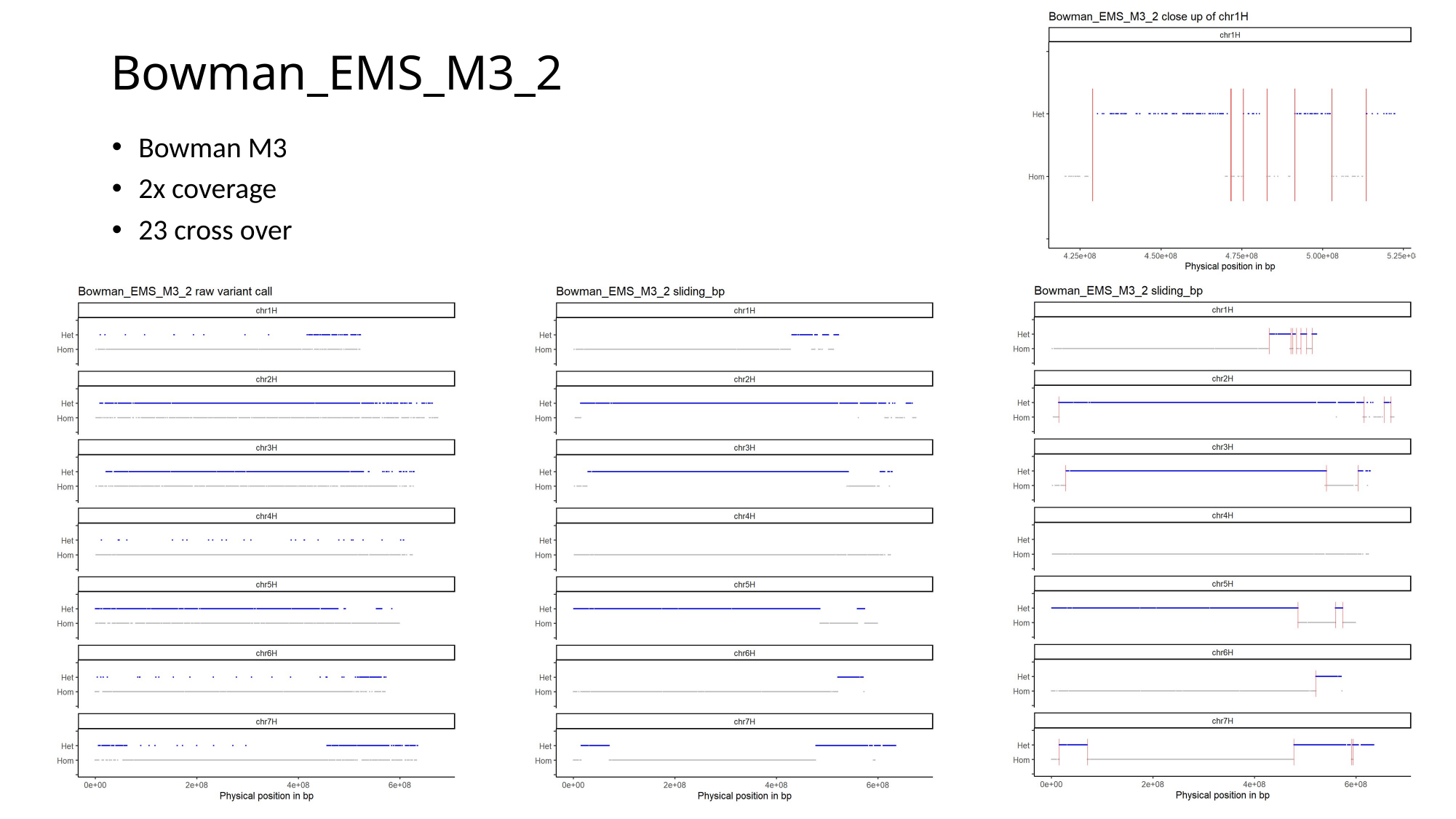

# Bowman_EMS_M3_2
Bowman M3
2x coverage
23 cross over

## Slide 4
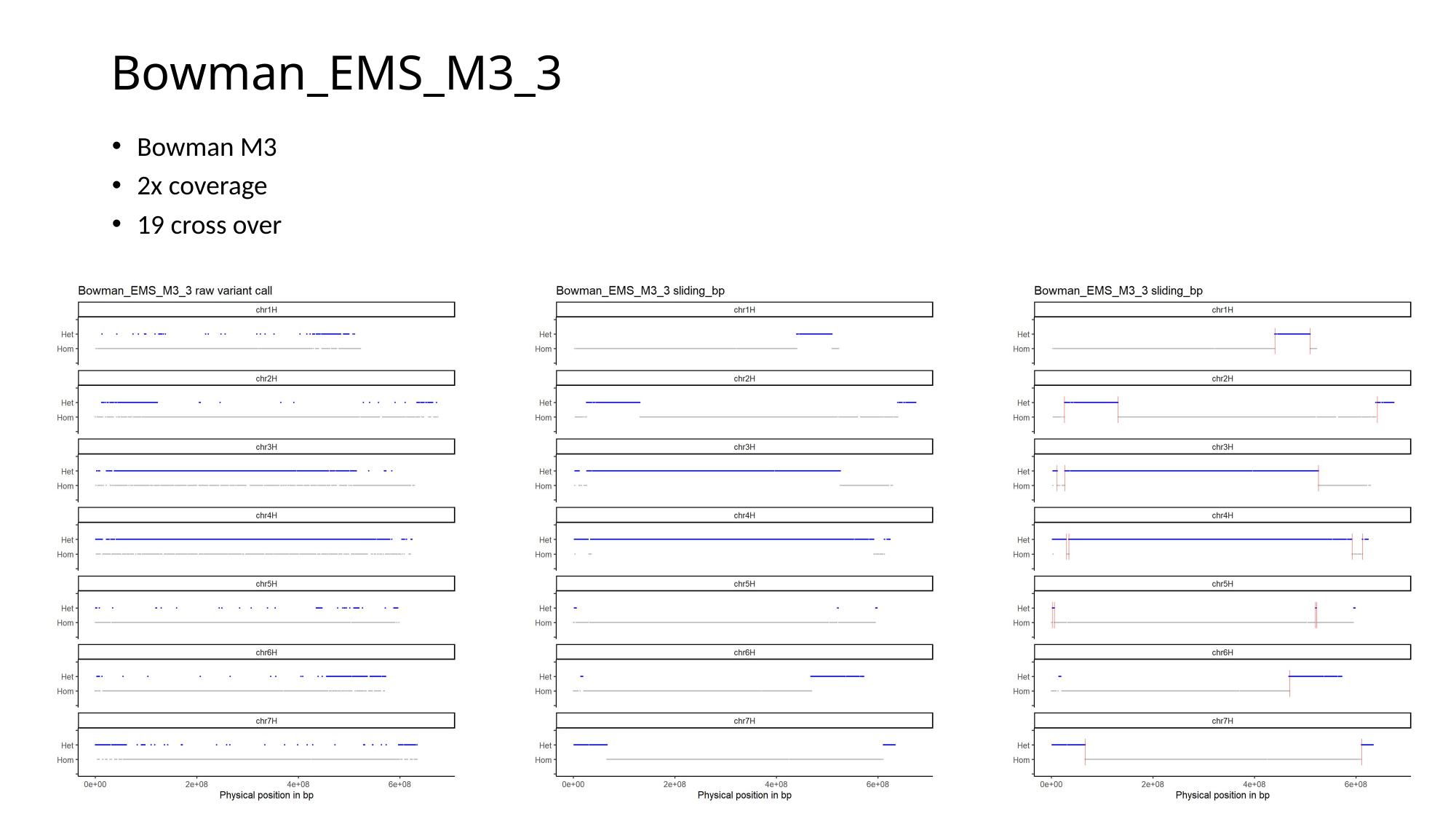

# Bowman_EMS_M3_3
Bowman M3
2x coverage
19 cross over

## Slide 5
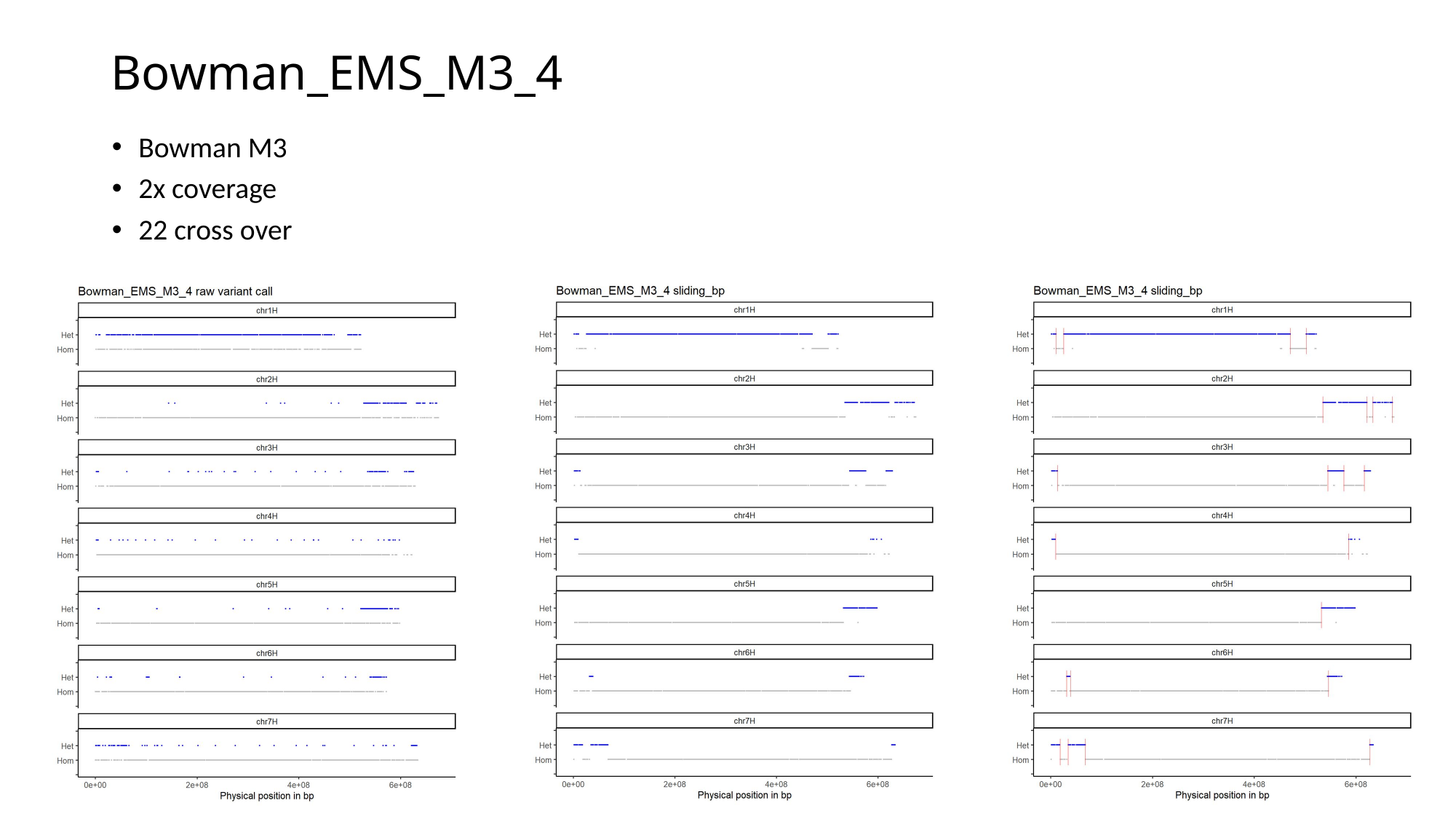

# Bowman_EMS_M3_4
Bowman M3
2x coverage
22 cross over

## Slide 6
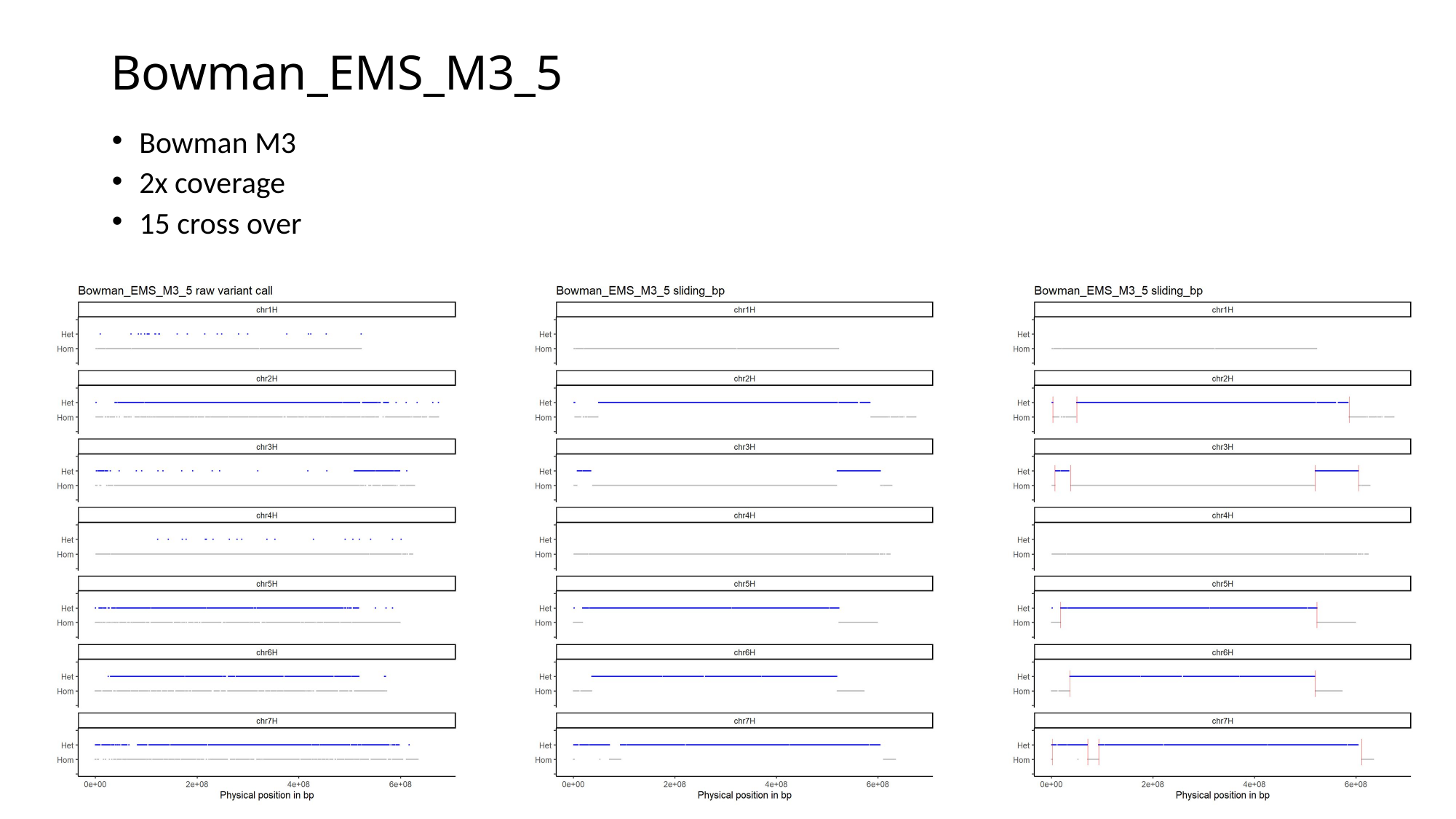

# Bowman_EMS_M3_5
Bowman M3
2x coverage
15 cross over

## Slide 7
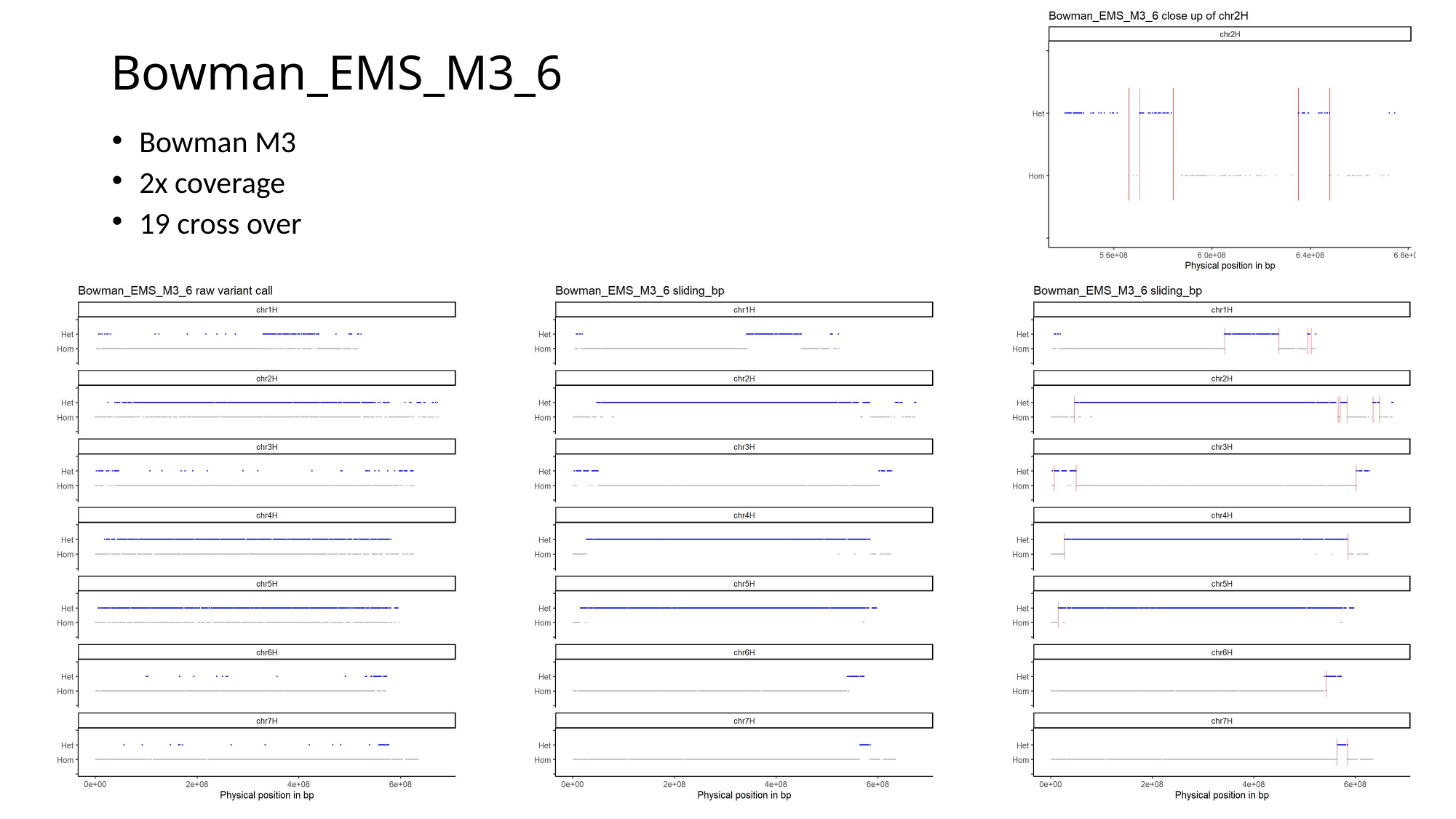

# Bowman_EMS_M3_6
Bowman M3
2x coverage
19 cross over

## Slide 8
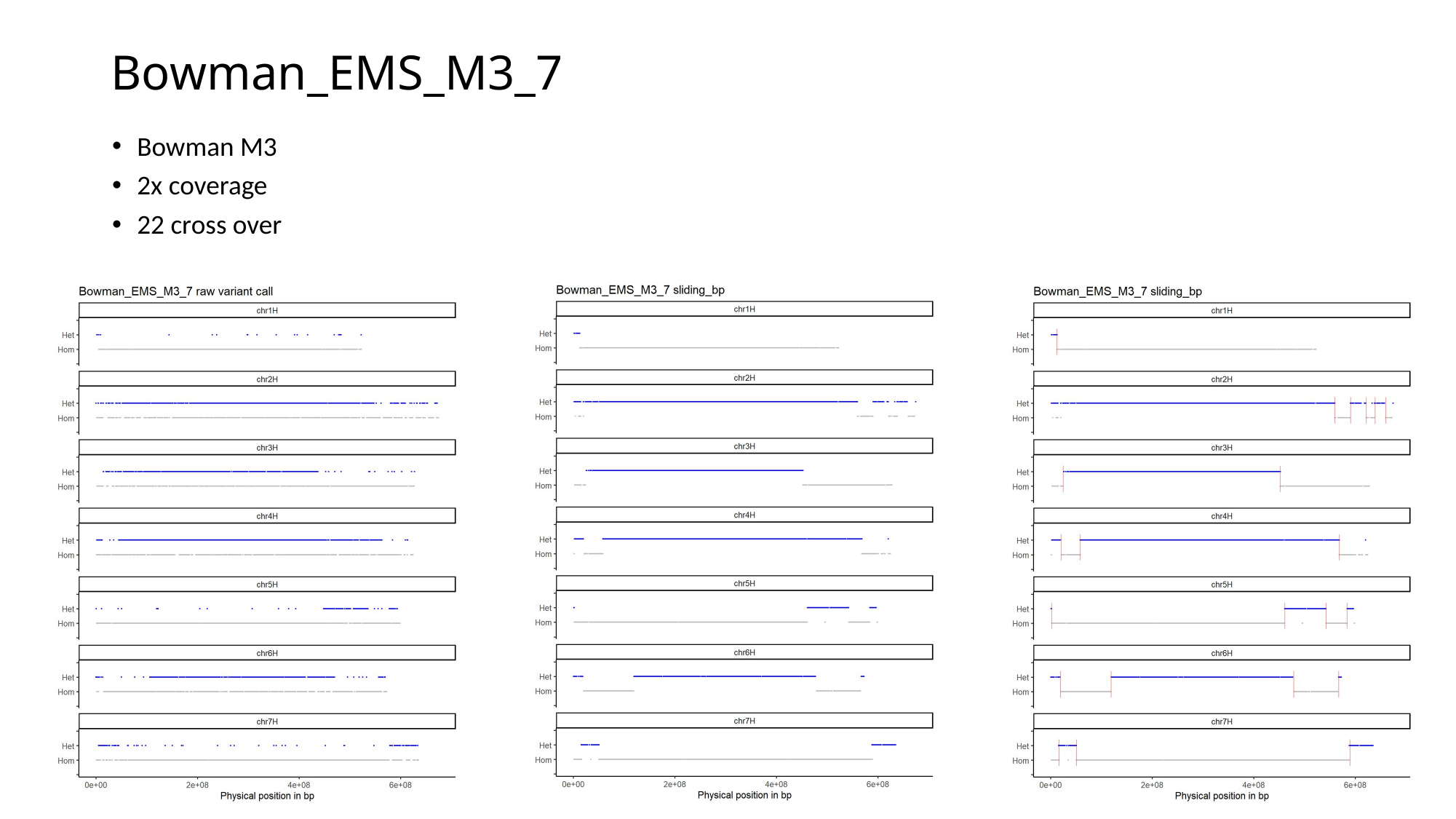

# Bowman_EMS_M3_7
Bowman M3
2x coverage
22 cross over

## Slide 9
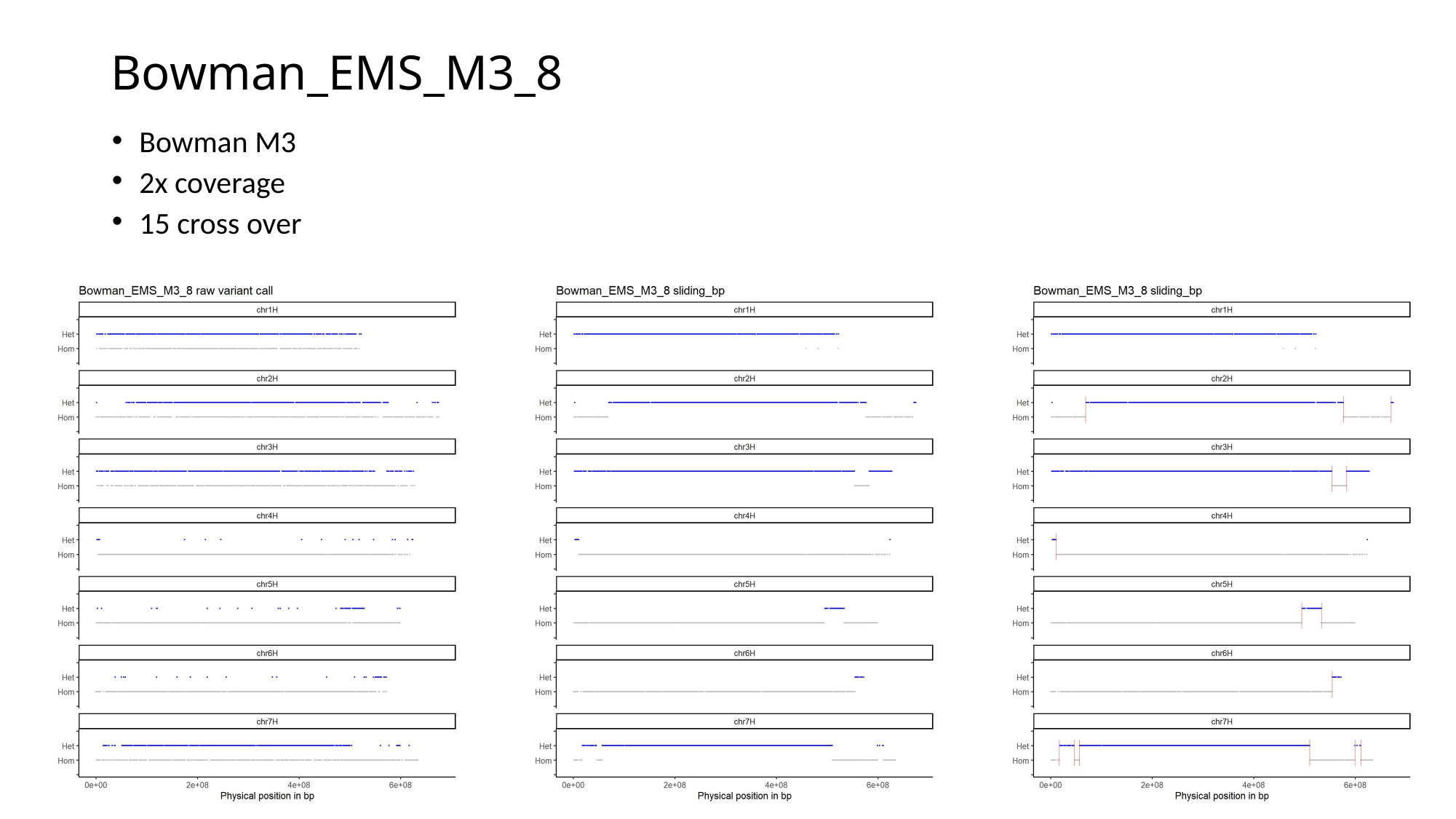

# Bowman_EMS_M3_8
Bowman M3
2x coverage
15 cross over

## Slide 10
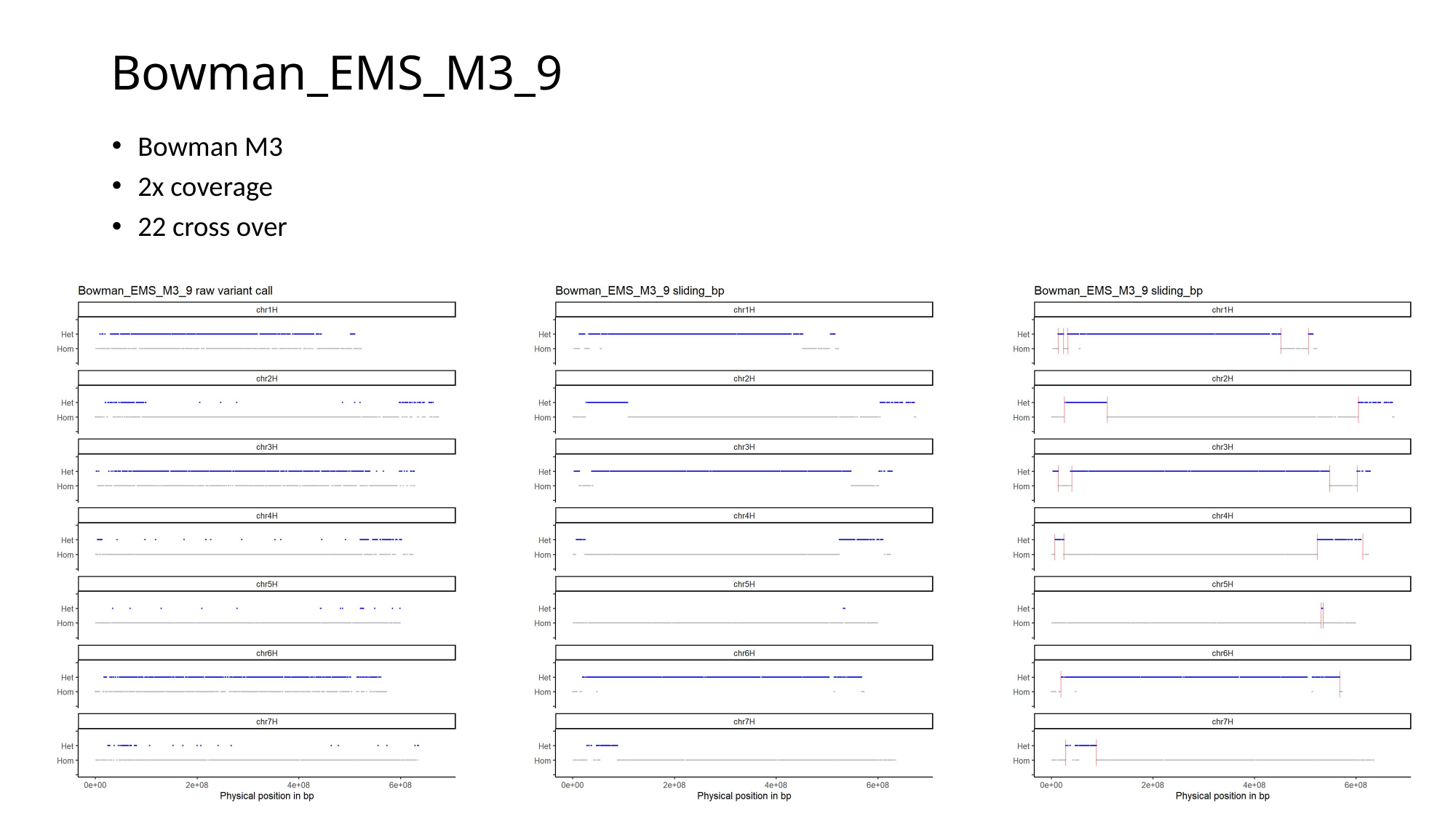

# Bowman_EMS_M3_9
Bowman M3
2x coverage
22 cross over

## Slide 11
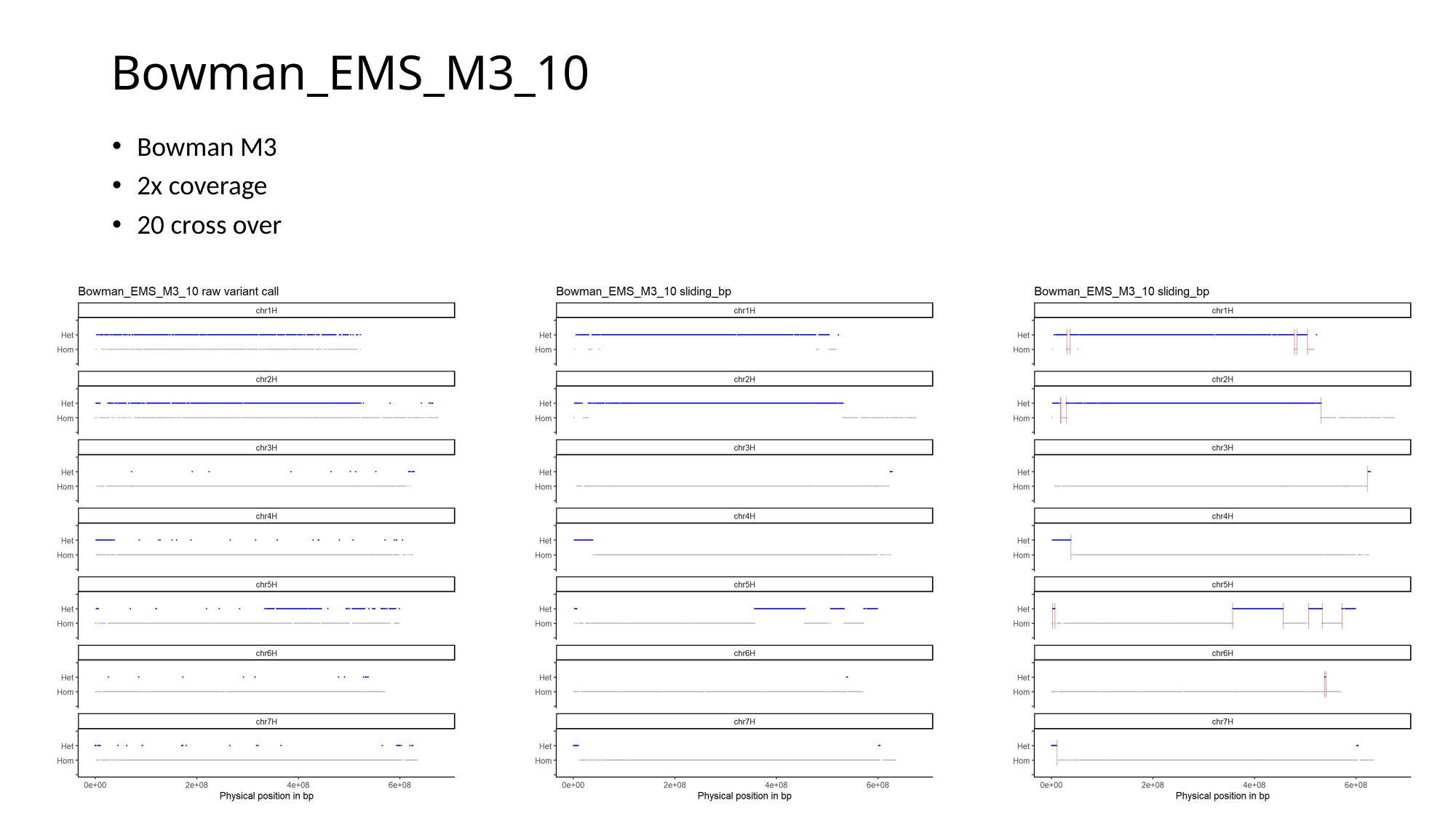

# Bowman_EMS_M3_10
Bowman M3
2x coverage
20 cross over

## Slide 12
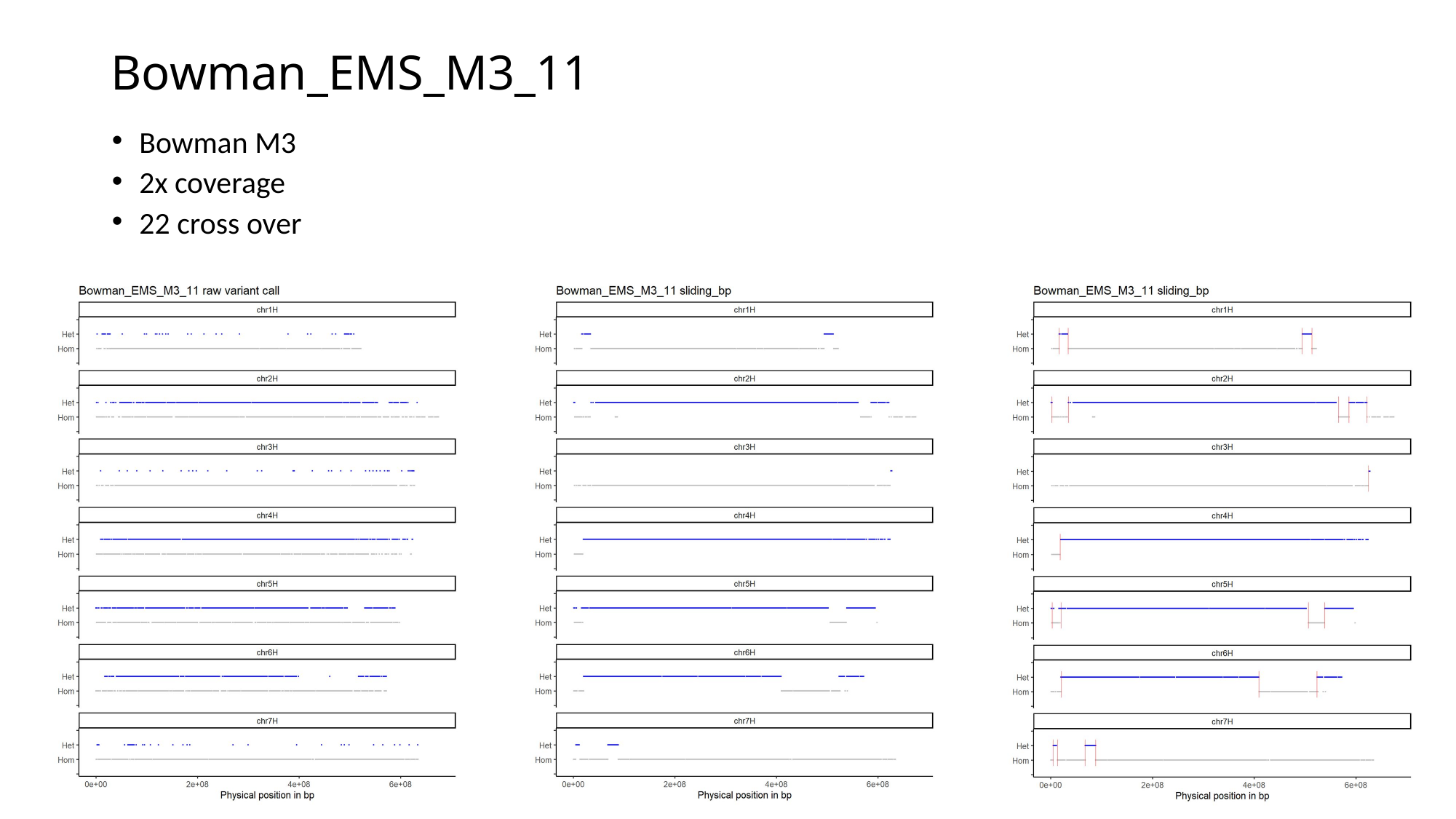

# Bowman_EMS_M3_11
Bowman M3
2x coverage
22 cross over

## Slide 13
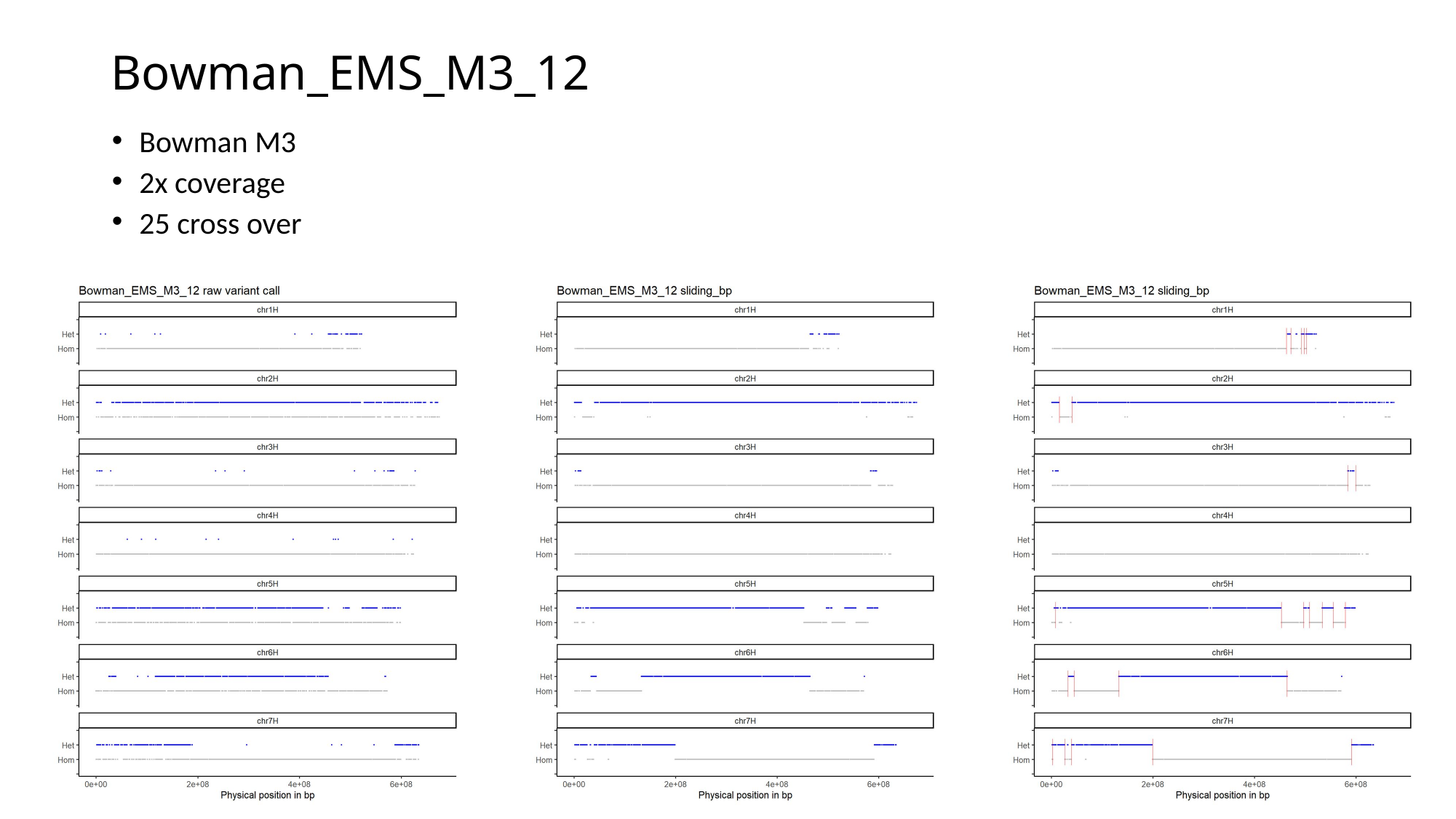

# Bowman_EMS_M3_12
Bowman M3
2x coverage
25 cross over

## Slide 14
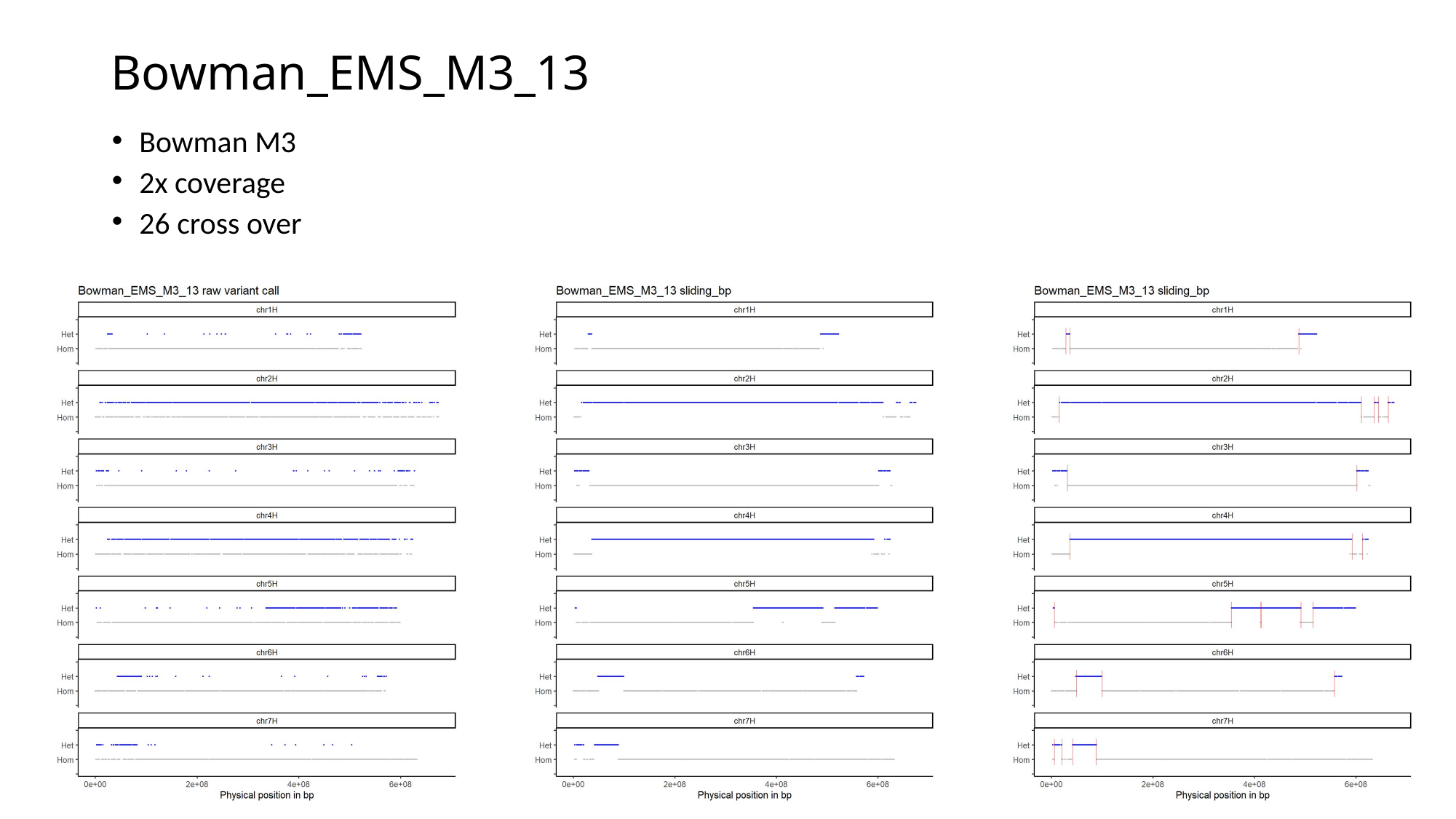

# Bowman_EMS_M3_13
Bowman M3
2x coverage
26 cross over

## Slide 15
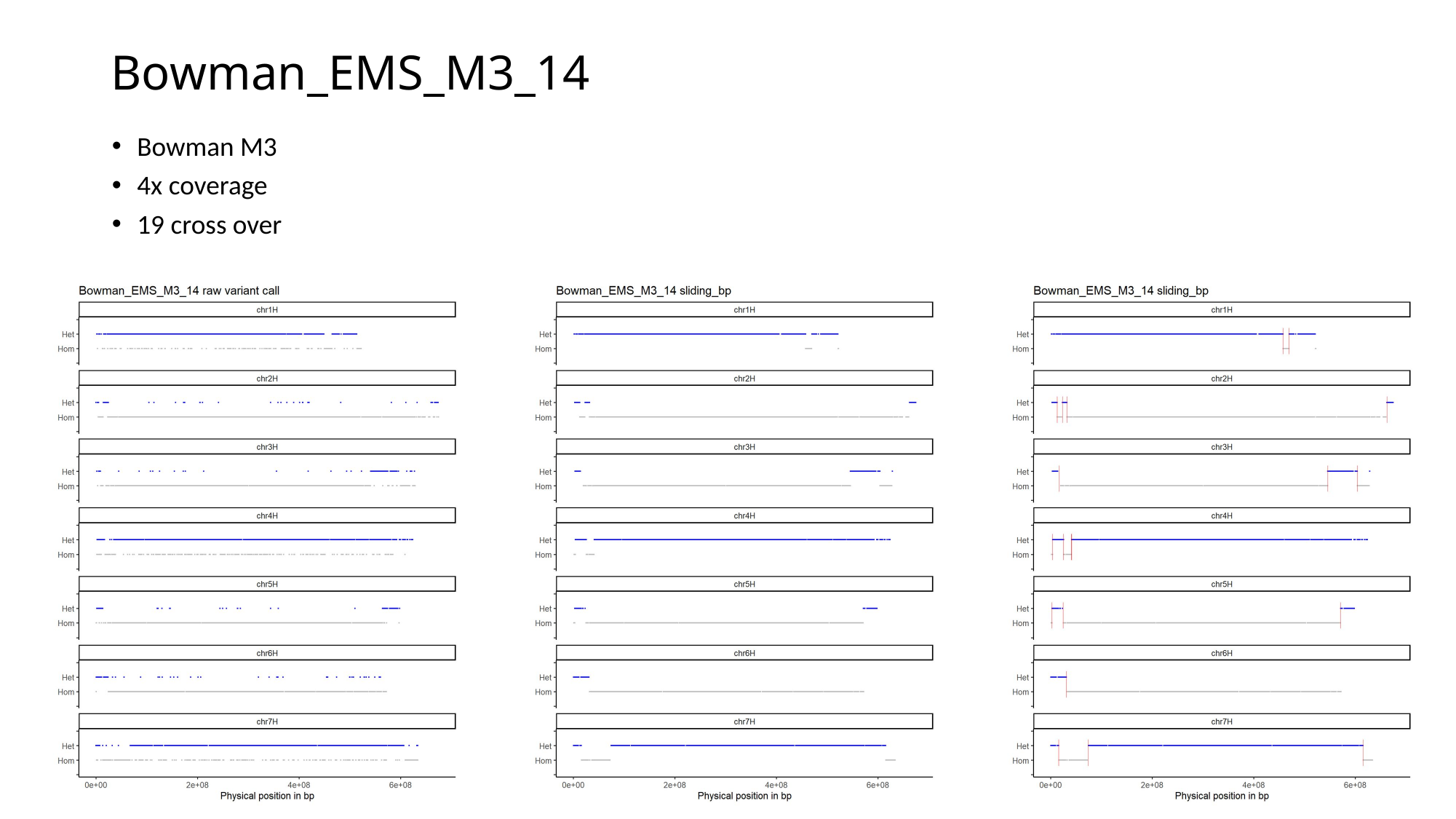

# Bowman_EMS_M3_14
Bowman M3
4x coverage
19 cross over

## Slide 16
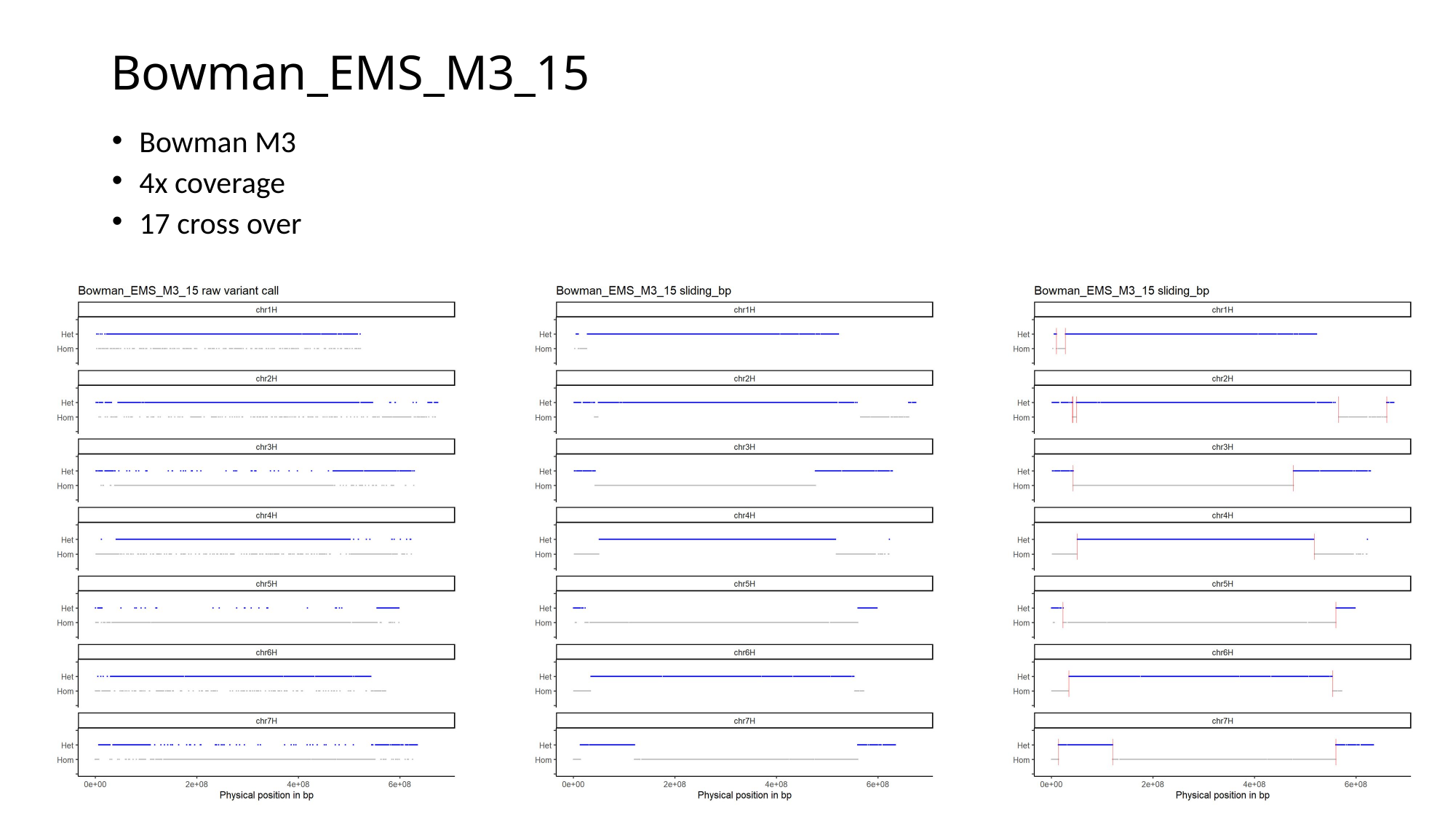

# Bowman_EMS_M3_15
Bowman M3
4x coverage
17 cross over

## Slide 17
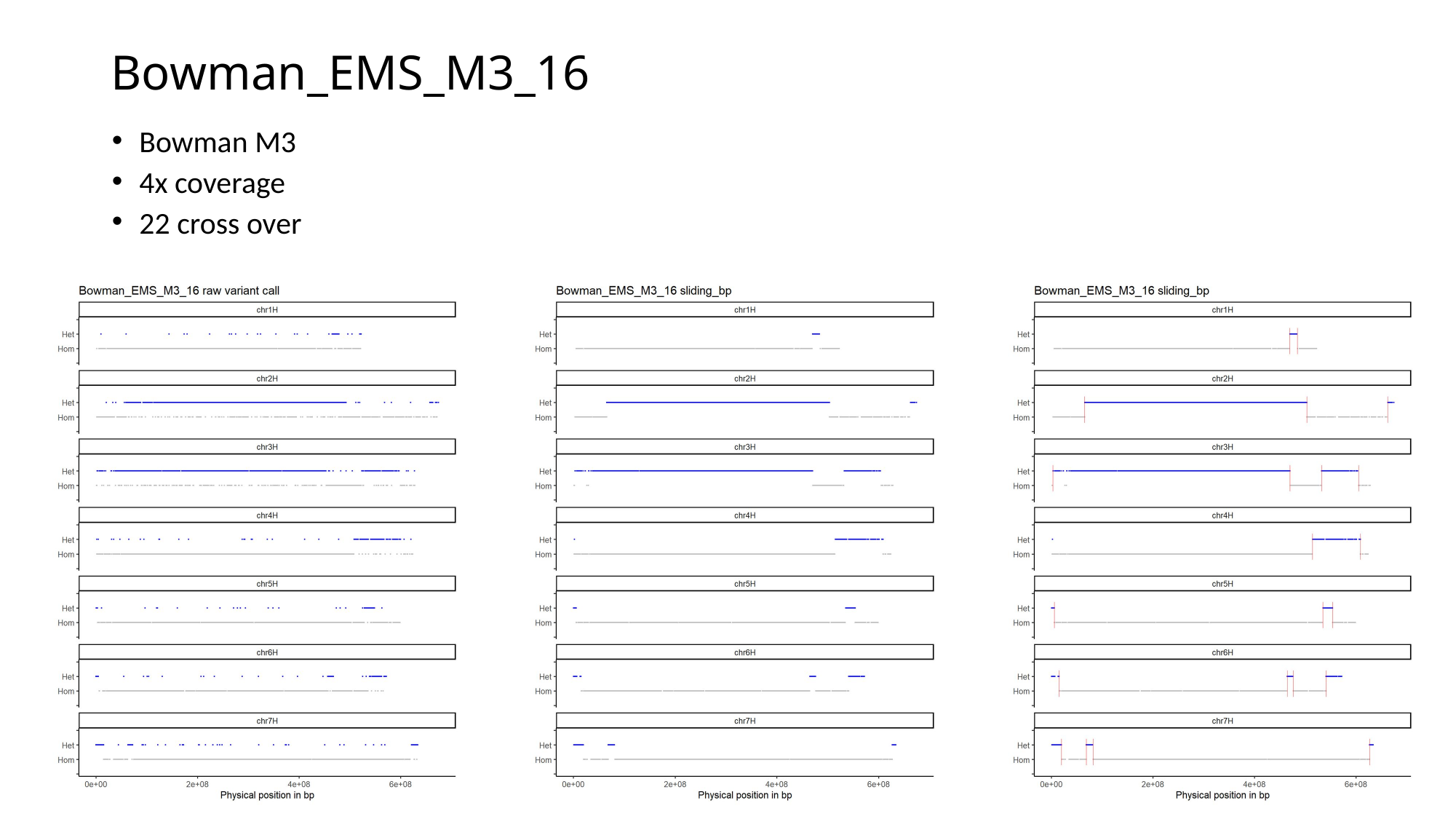

# Bowman_EMS_M3_16
Bowman M3
4x coverage
22 cross over

## Slide 18
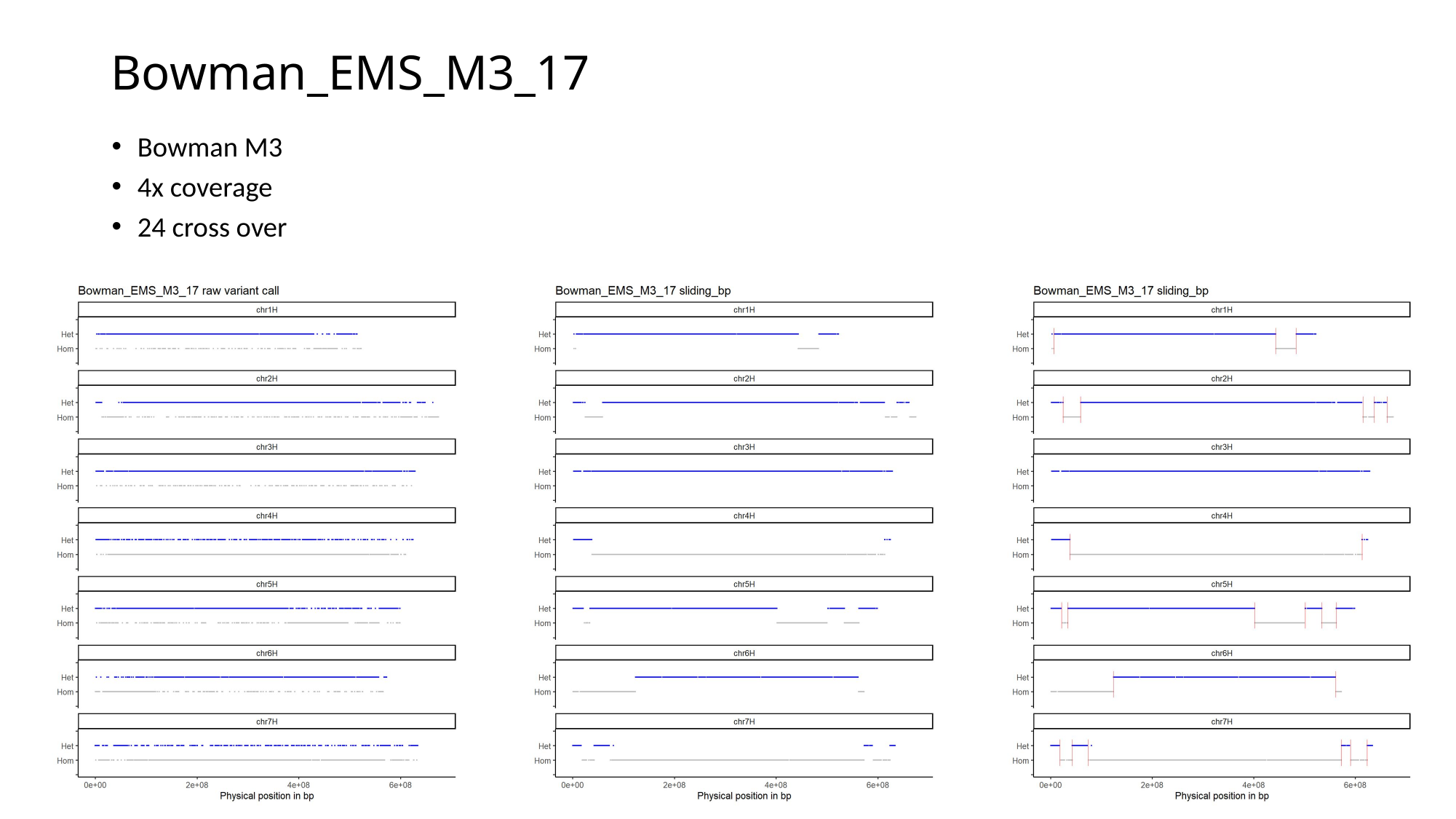

# Bowman_EMS_M3_17
Bowman M3
4x coverage
24 cross over

## Slide 19
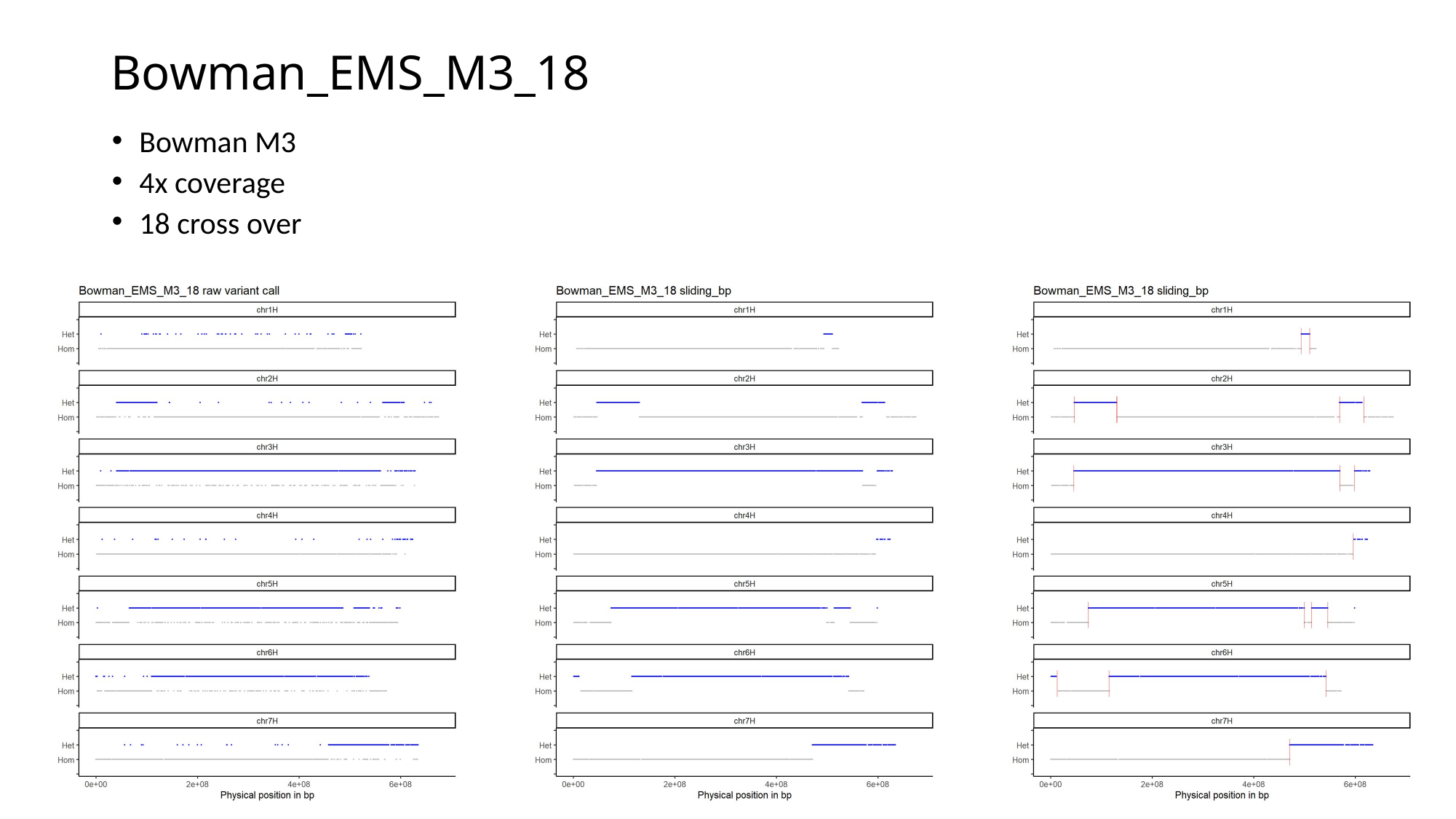

# Bowman_EMS_M3_18
Bowman M3
4x coverage
18 cross over

## Slide 20
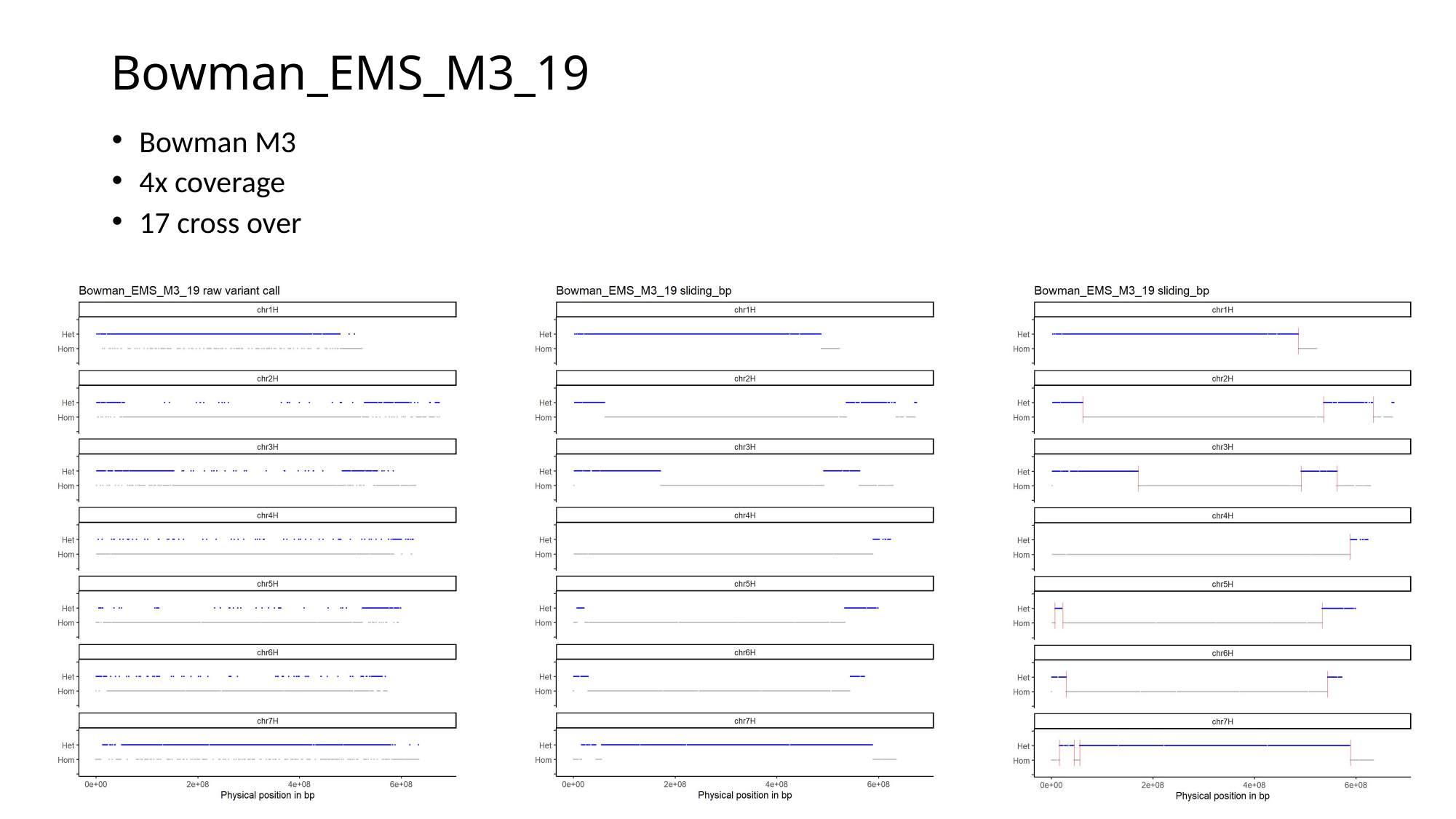

# Bowman_EMS_M3_19
Bowman M3
4x coverage
17 cross over

## Slide 21
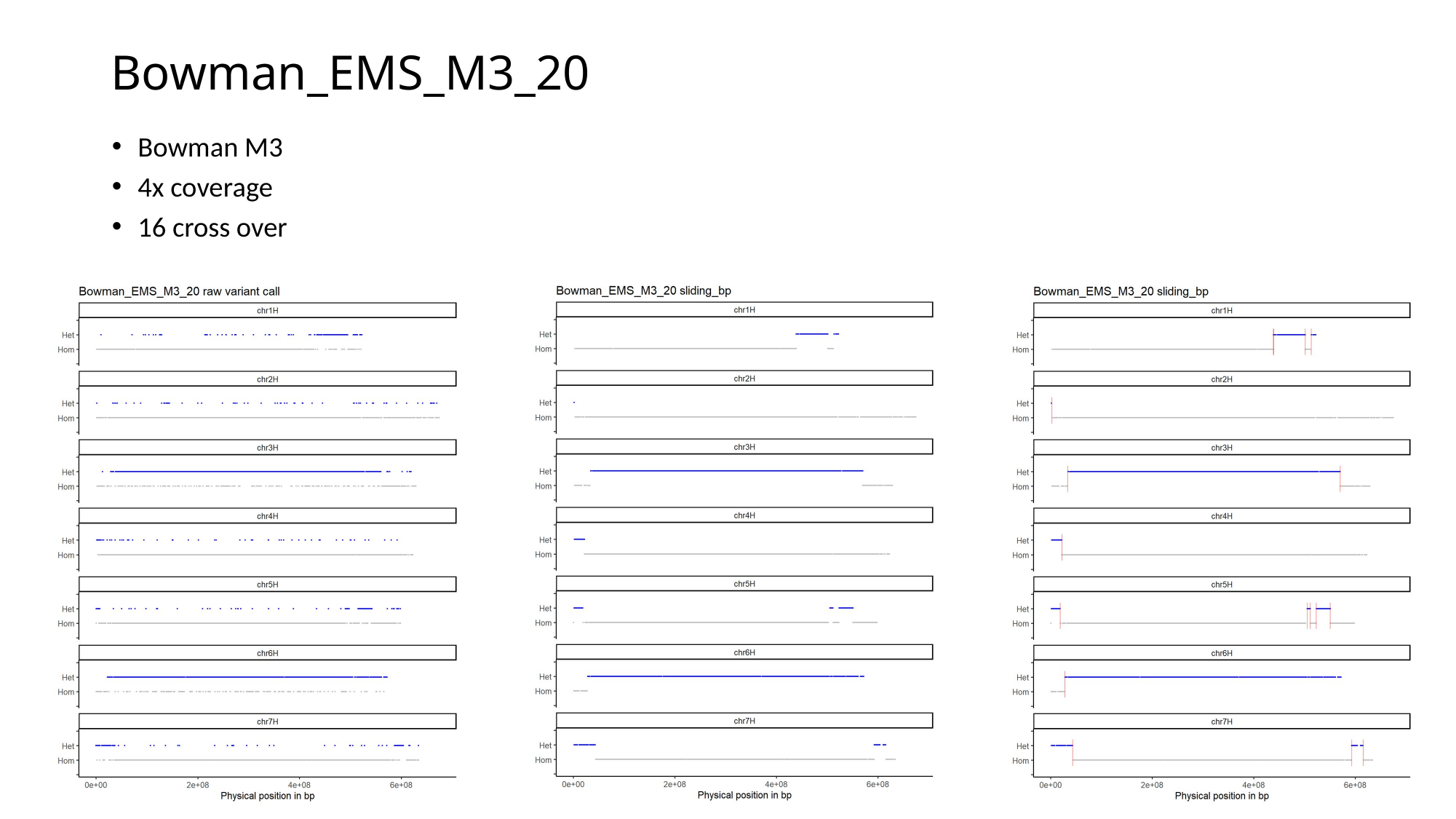

# Bowman_EMS_M3_20
Bowman M3
4x coverage
16 cross over

## Slide 22
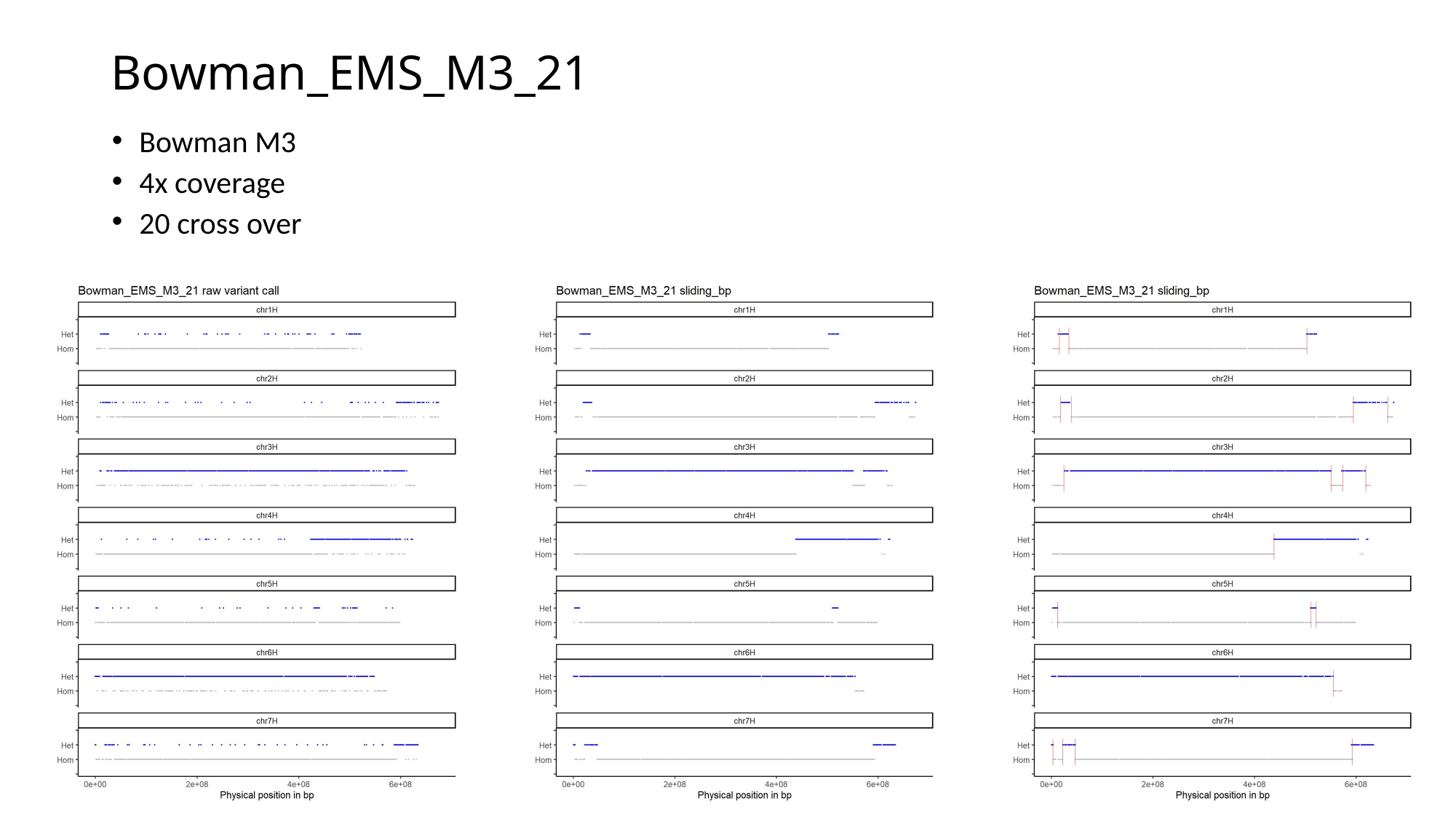

# Bowman_EMS_M3_21
Bowman M3
4x coverage
20 cross over

## Slide 23
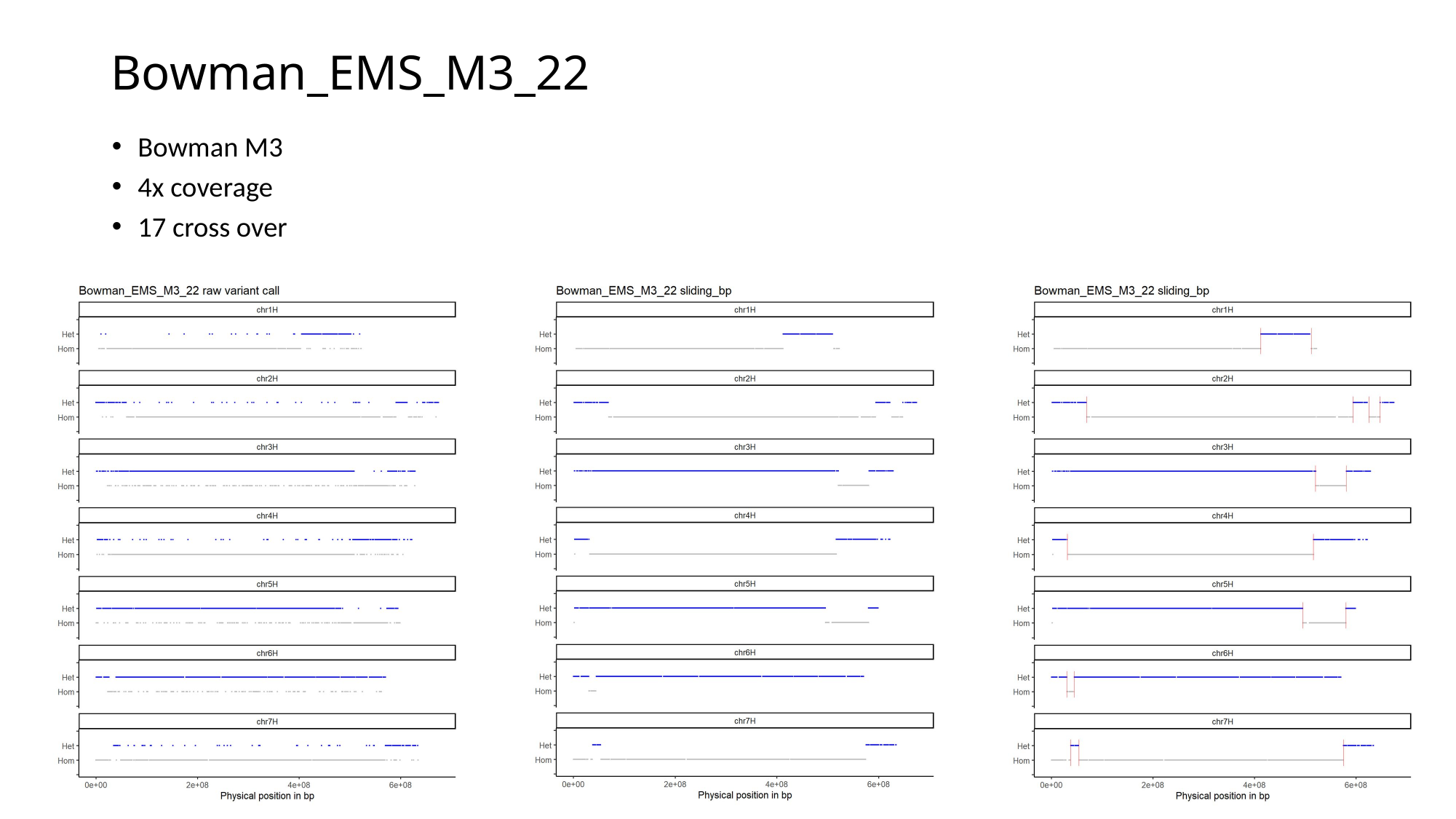

# Bowman_EMS_M3_22
Bowman M3
4x coverage
17 cross over

## Slide 24
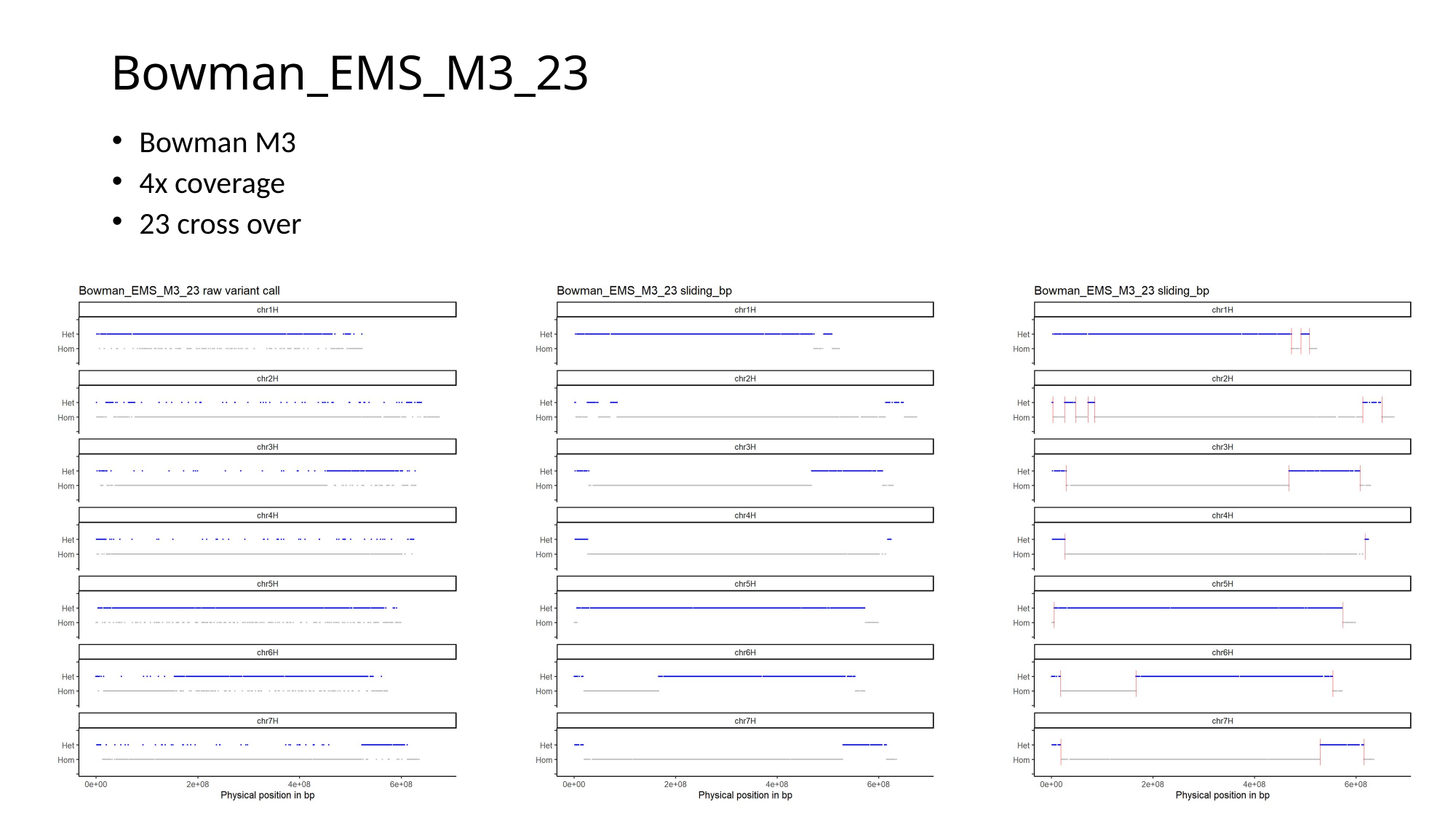

# Bowman_EMS_M3_23
Bowman M3
4x coverage
23 cross over

## Slide 25
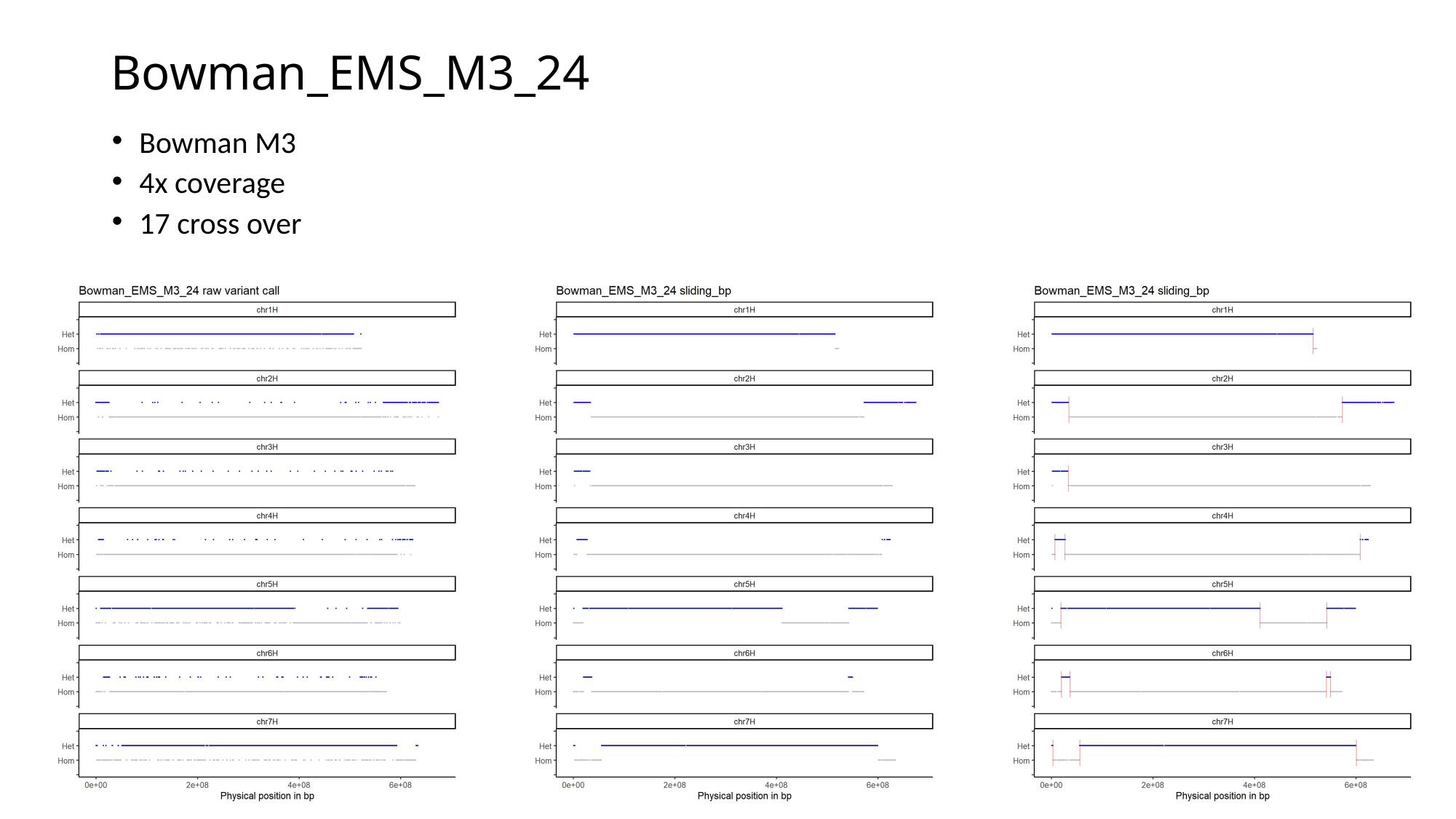

# Bowman_EMS_M3_24
Bowman M3
4x coverage
17 cross over

## Slide 26
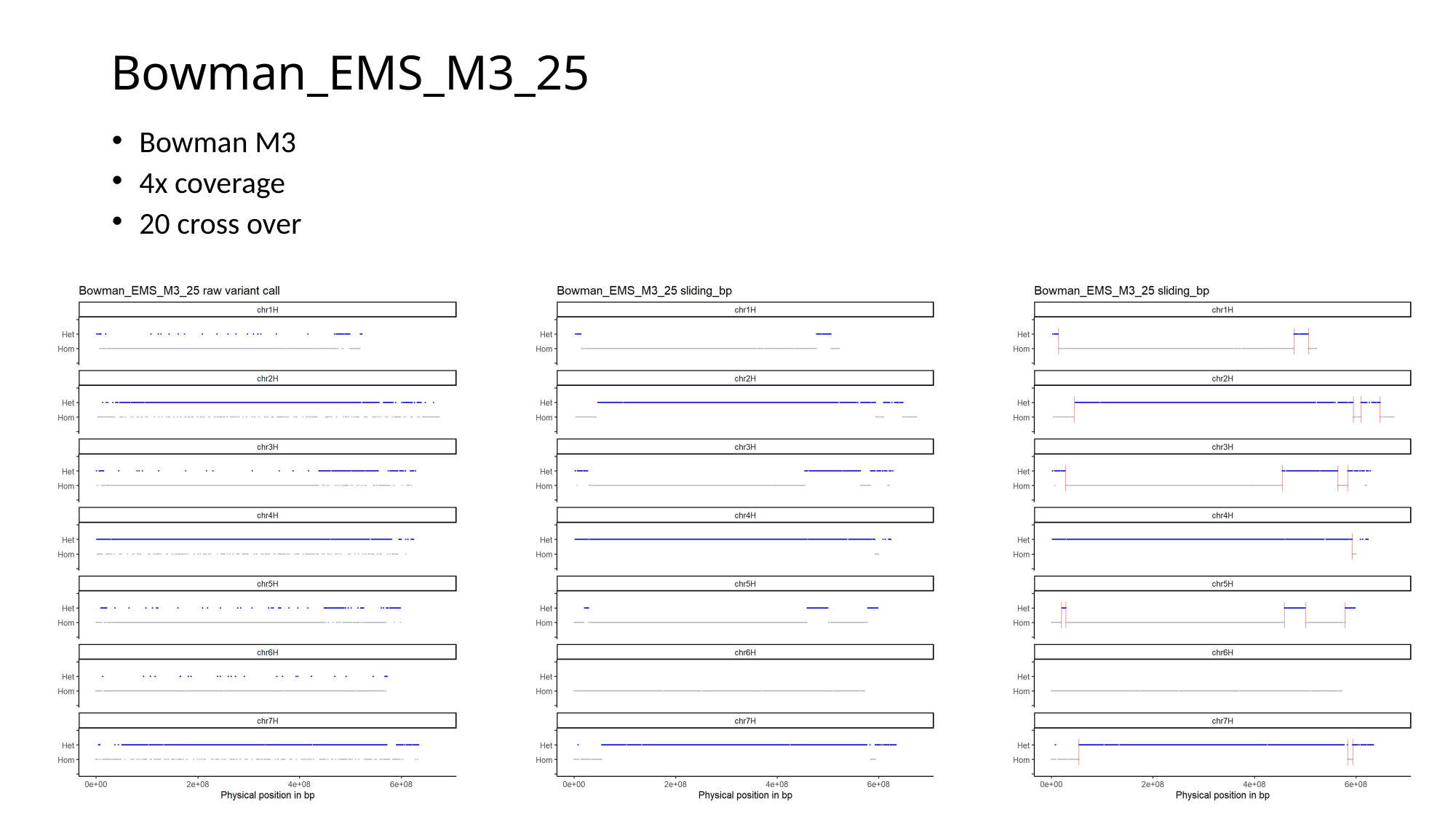

# Bowman_EMS_M3_25
Bowman M3
4x coverage
20 cross over

## Slide 27
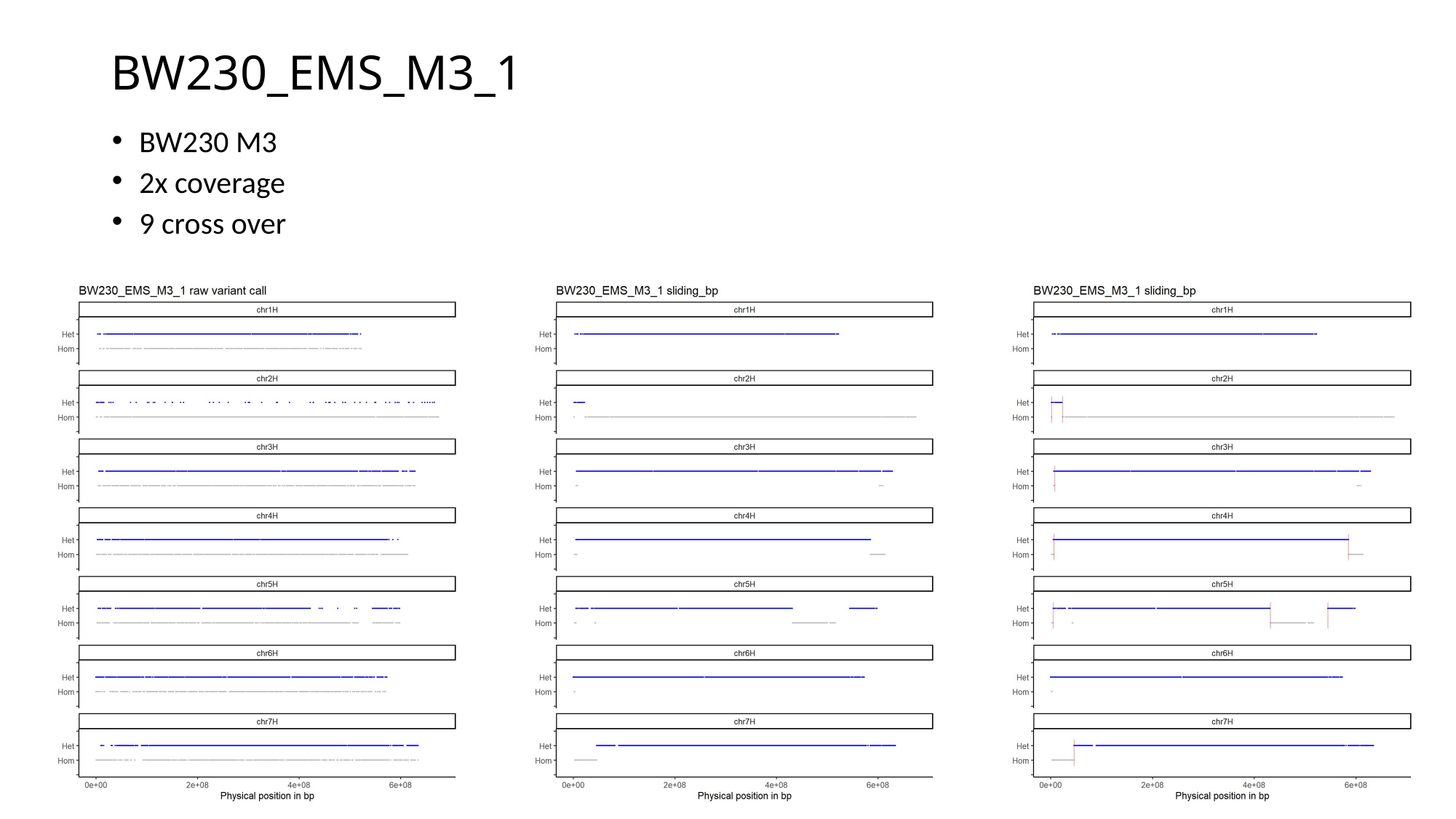

# BW230_EMS_M3_1
BW230 M3
2x coverage
9 cross over

## Slide 28
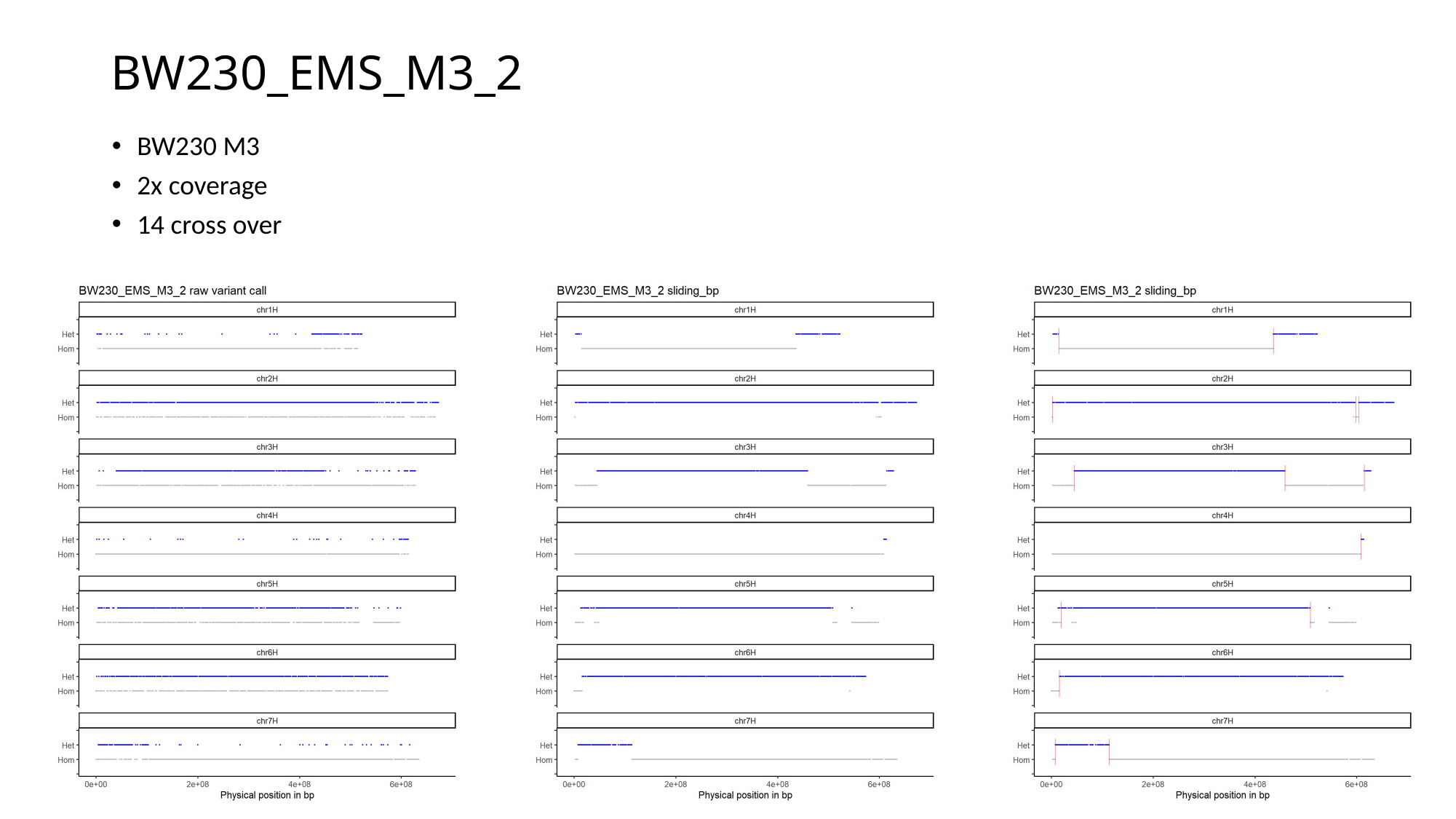

# BW230_EMS_M3_2
BW230 M3
2x coverage
14 cross over

## Slide 29
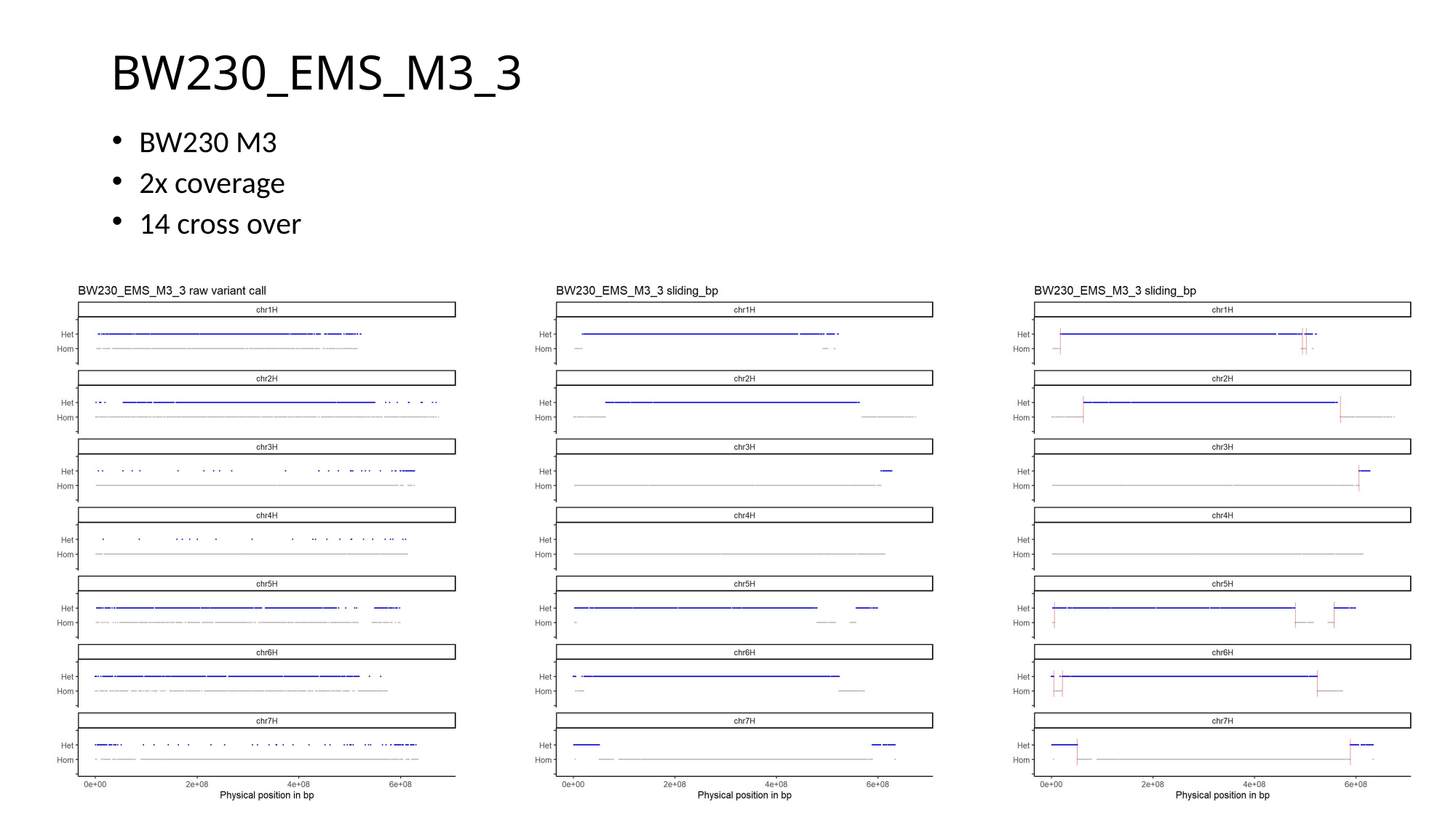

# BW230_EMS_M3_3
BW230 M3
2x coverage
14 cross over

## Slide 30
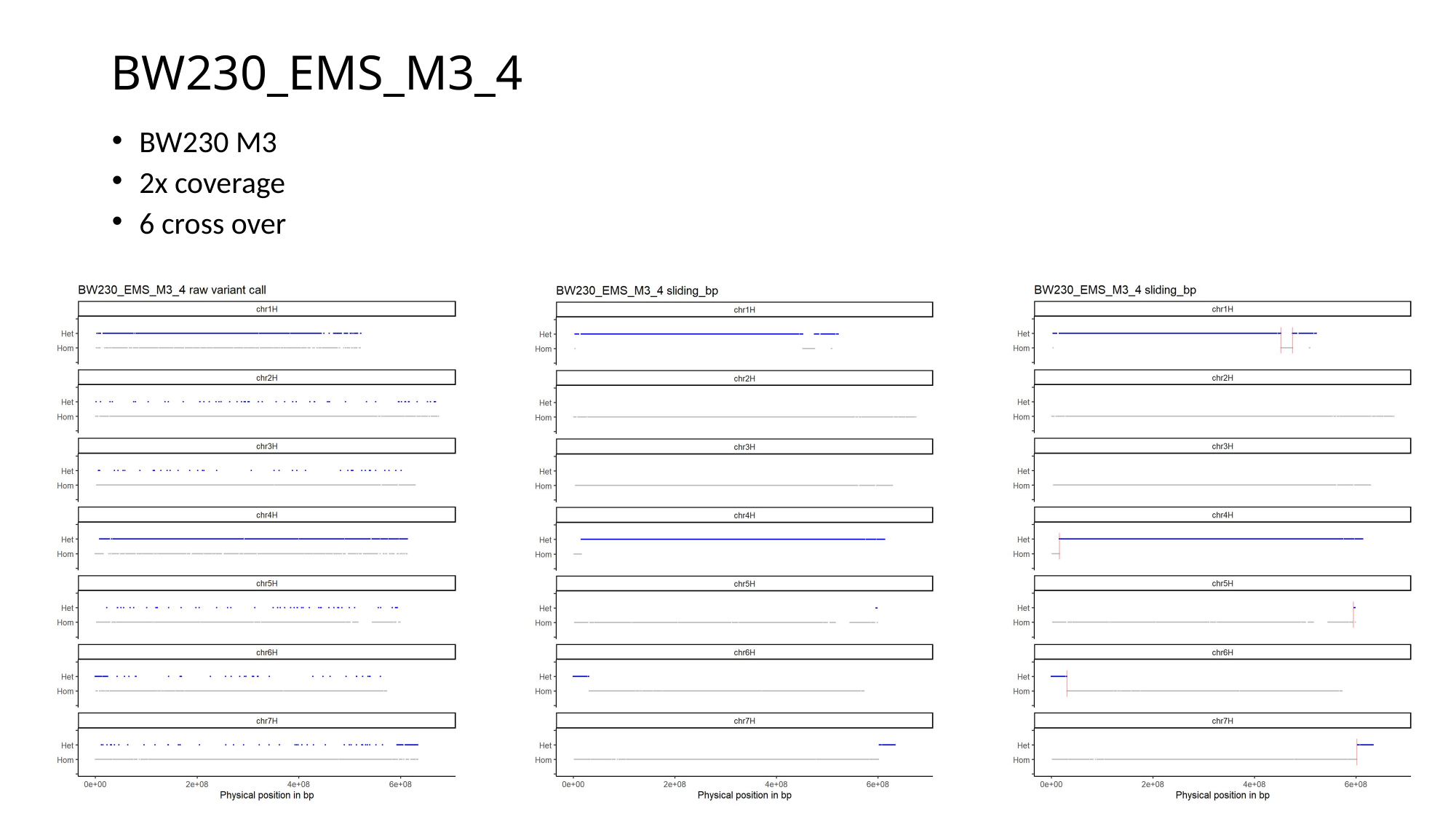

# BW230_EMS_M3_4
BW230 M3
2x coverage
6 cross over

## Slide 31
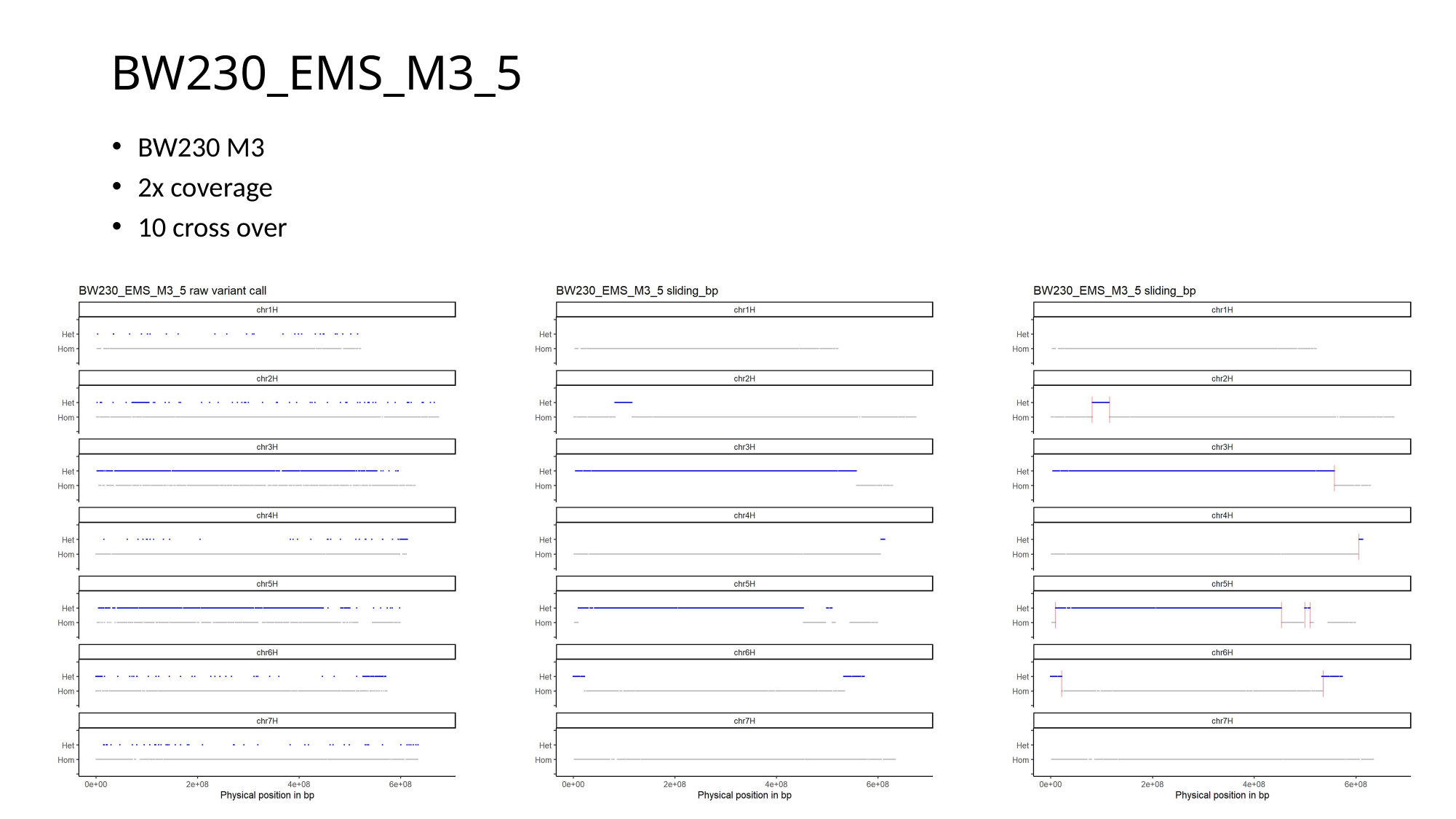

# BW230_EMS_M3_5
BW230 M3
2x coverage
10 cross over

## Slide 32
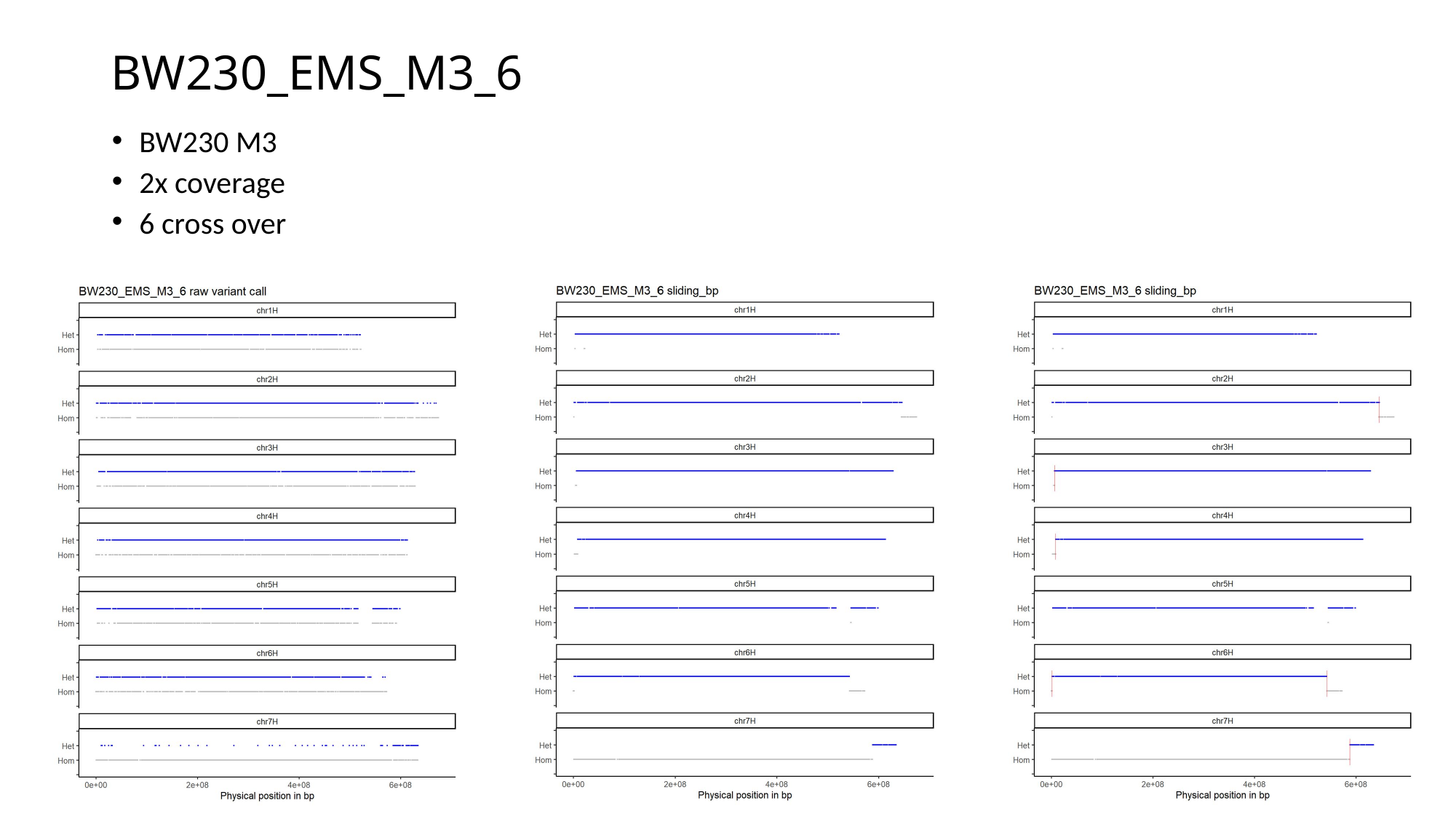

# BW230_EMS_M3_6
BW230 M3
2x coverage
6 cross over

## Slide 33
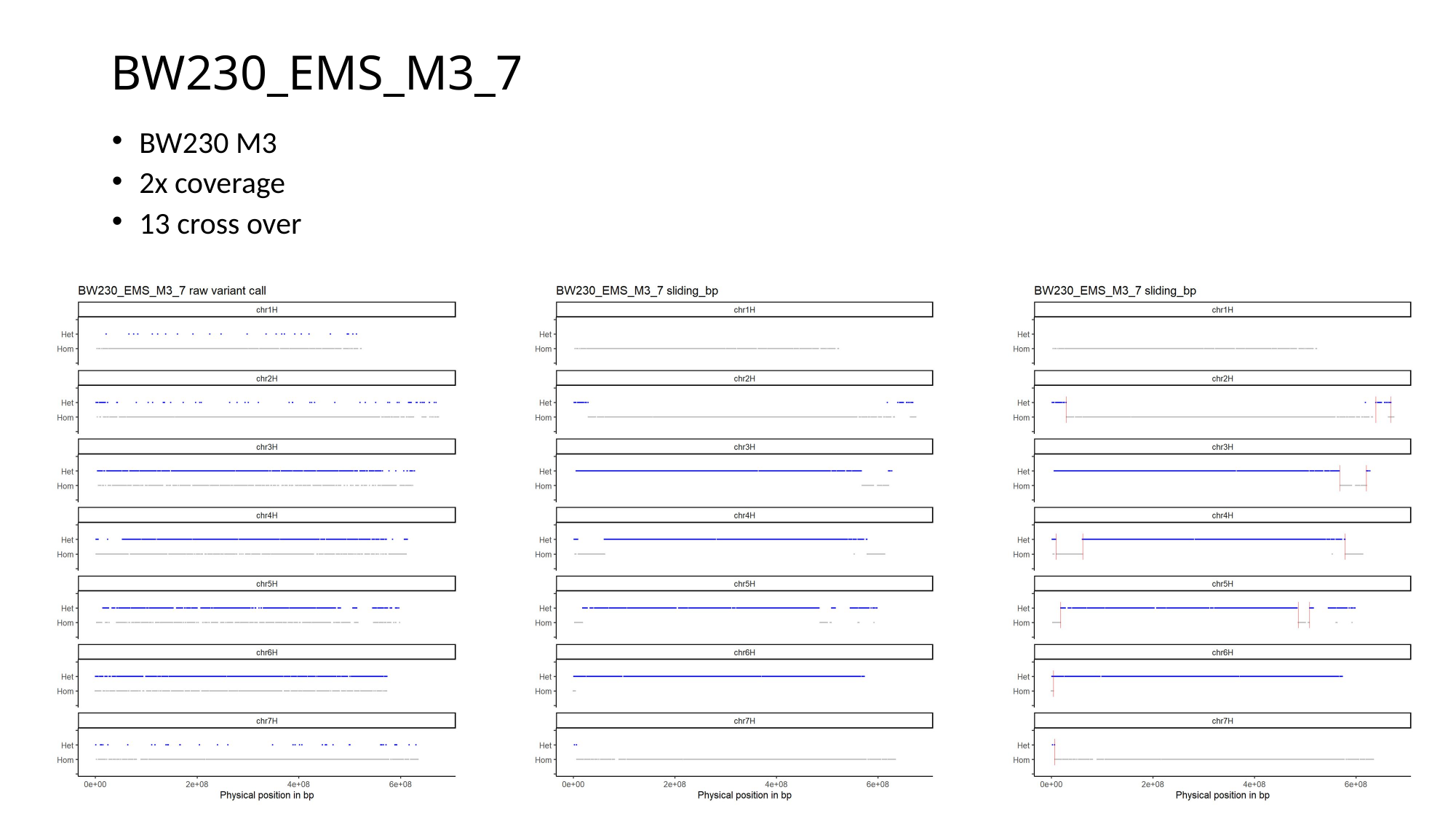

# BW230_EMS_M3_7
BW230 M3
2x coverage
13 cross over

## Slide 34
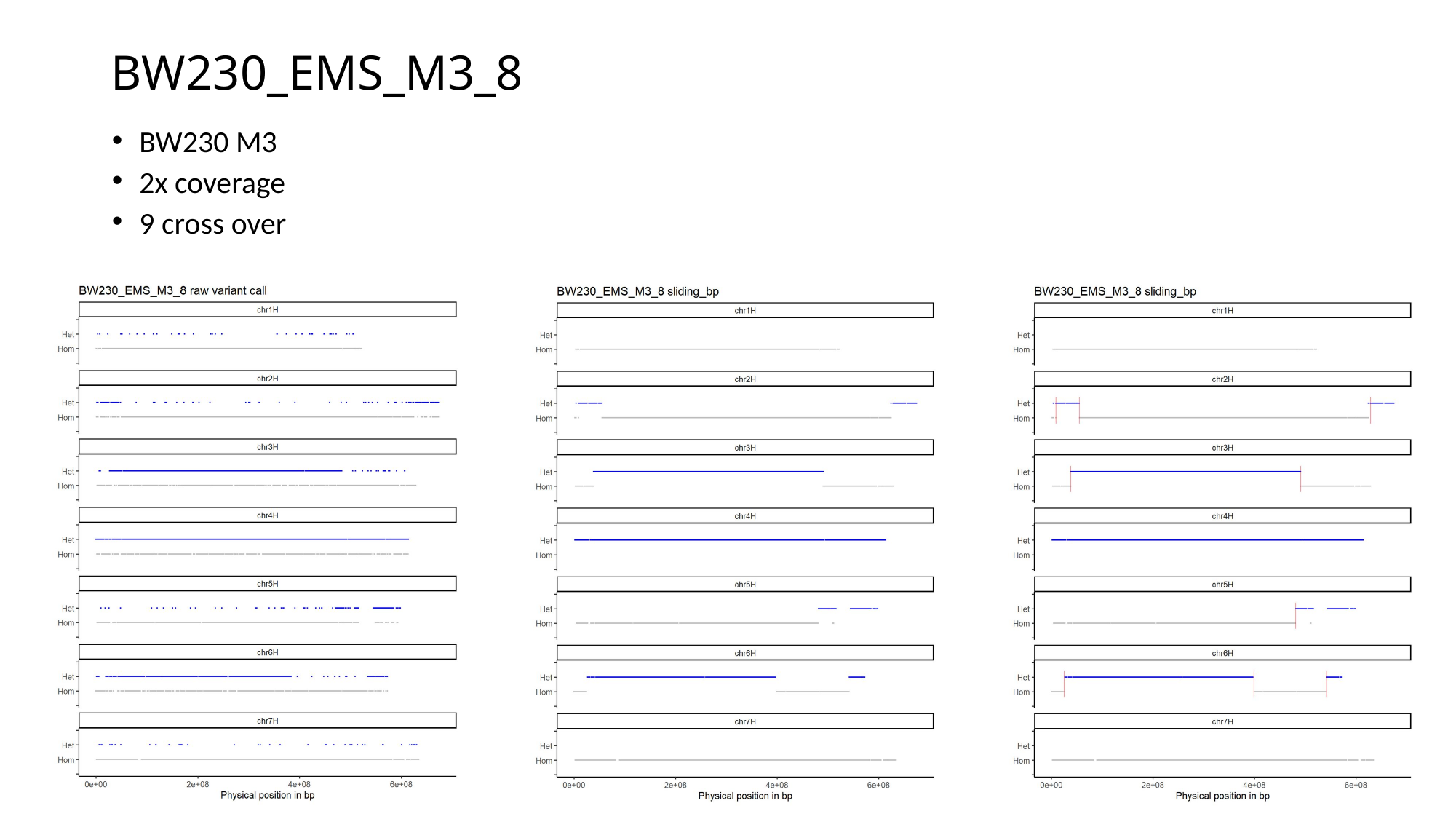

# BW230_EMS_M3_8
BW230 M3
2x coverage
9 cross over

## Slide 35
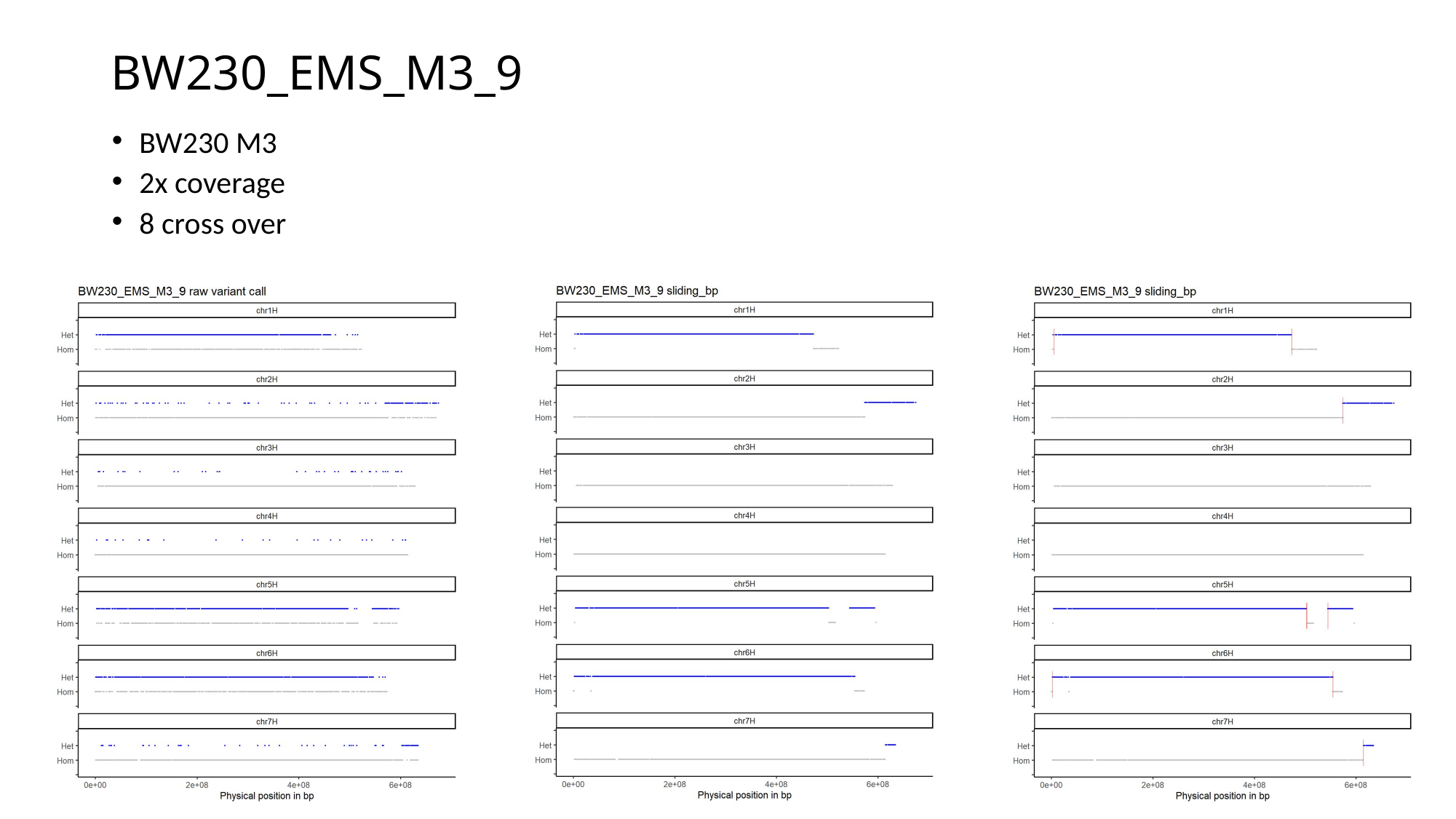

# BW230_EMS_M3_9
BW230 M3
2x coverage
8 cross over

## Slide 36
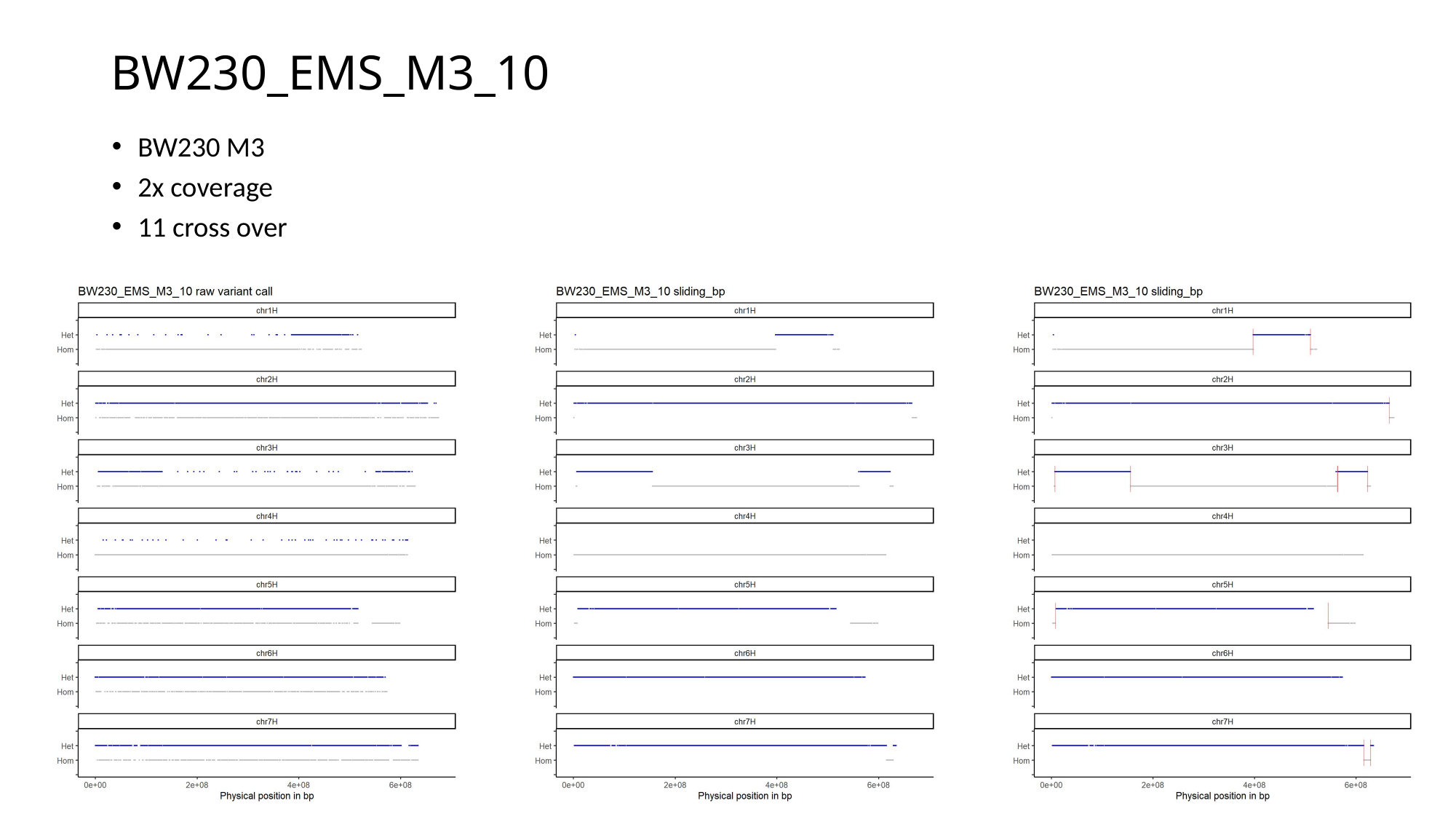

# BW230_EMS_M3_10
BW230 M3
2x coverage
11 cross over

## Slide 37
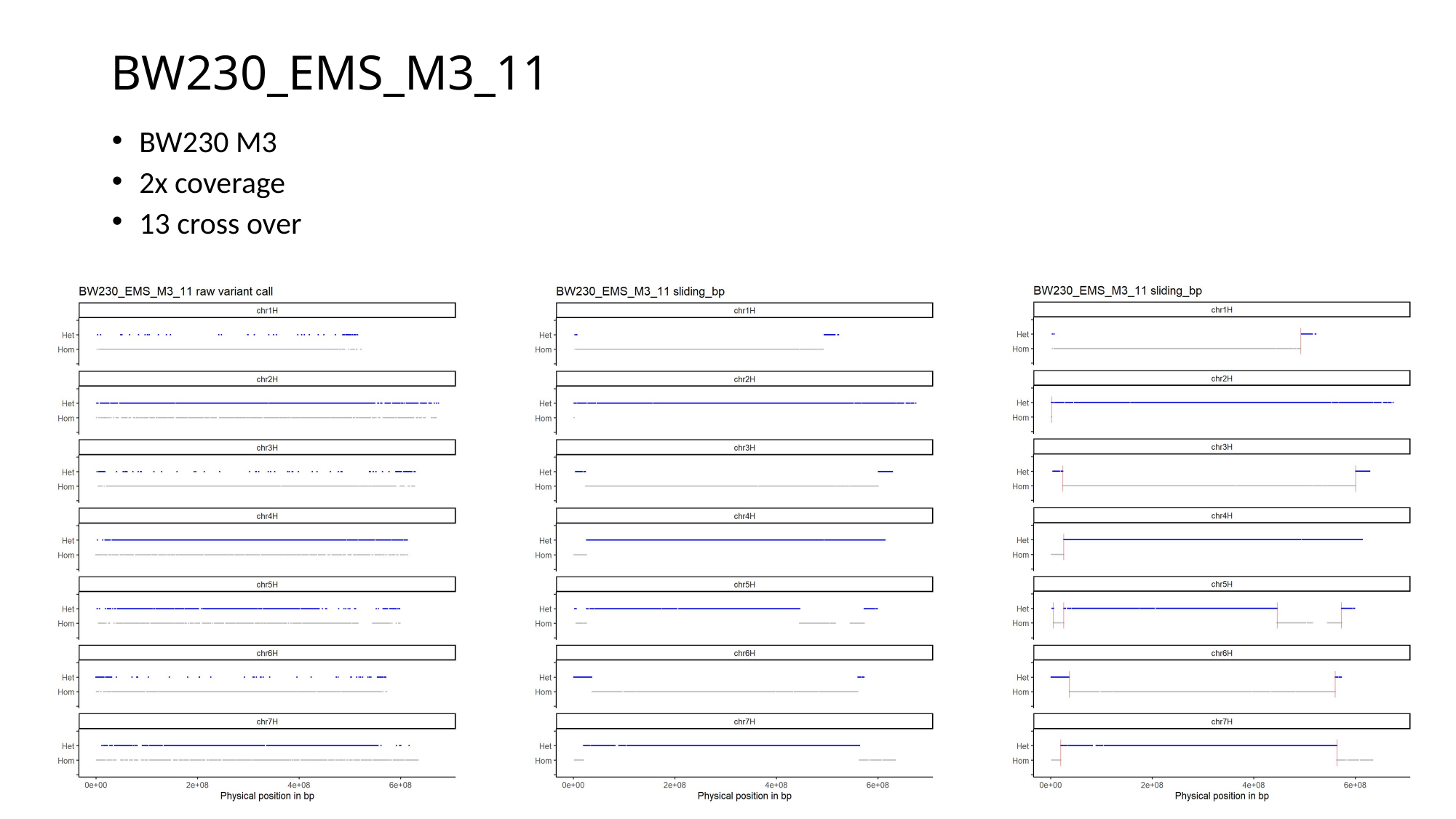

# BW230_EMS_M3_11
BW230 M3
2x coverage
13 cross over

## Slide 38
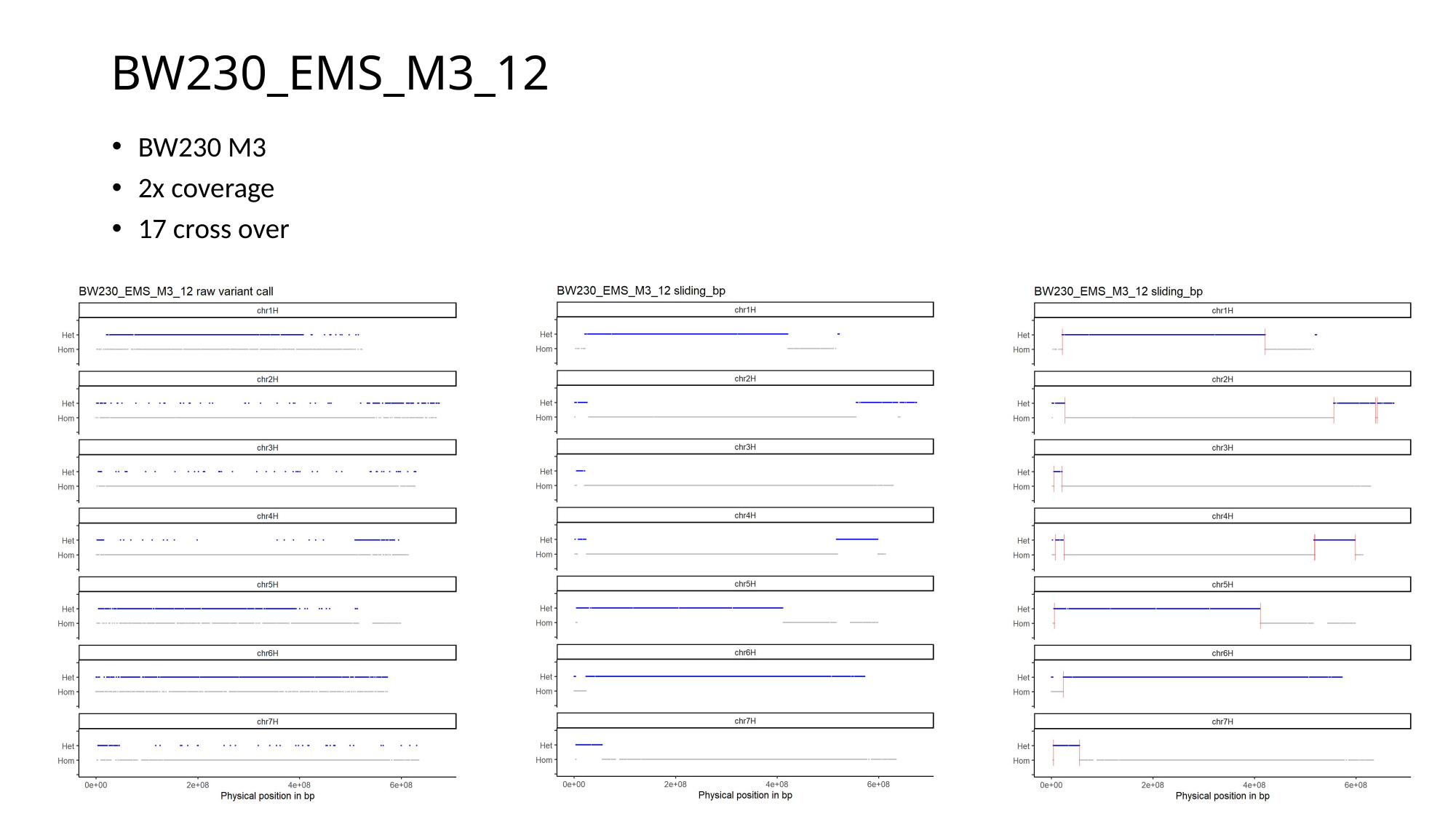

# BW230_EMS_M3_12
BW230 M3
2x coverage
17 cross over

## Slide 39
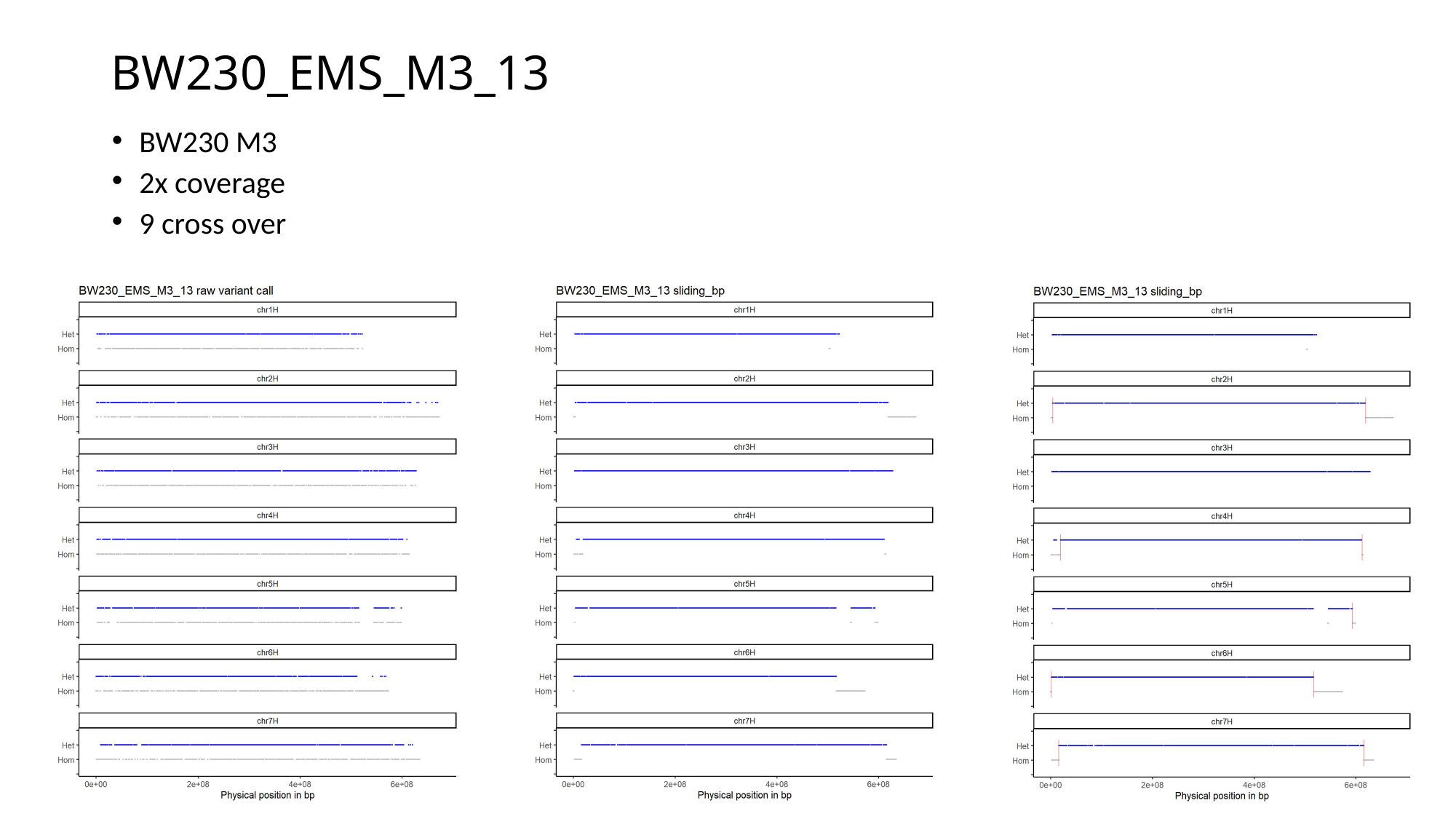

# BW230_EMS_M3_13
BW230 M3
2x coverage
9 cross over

## Slide 40
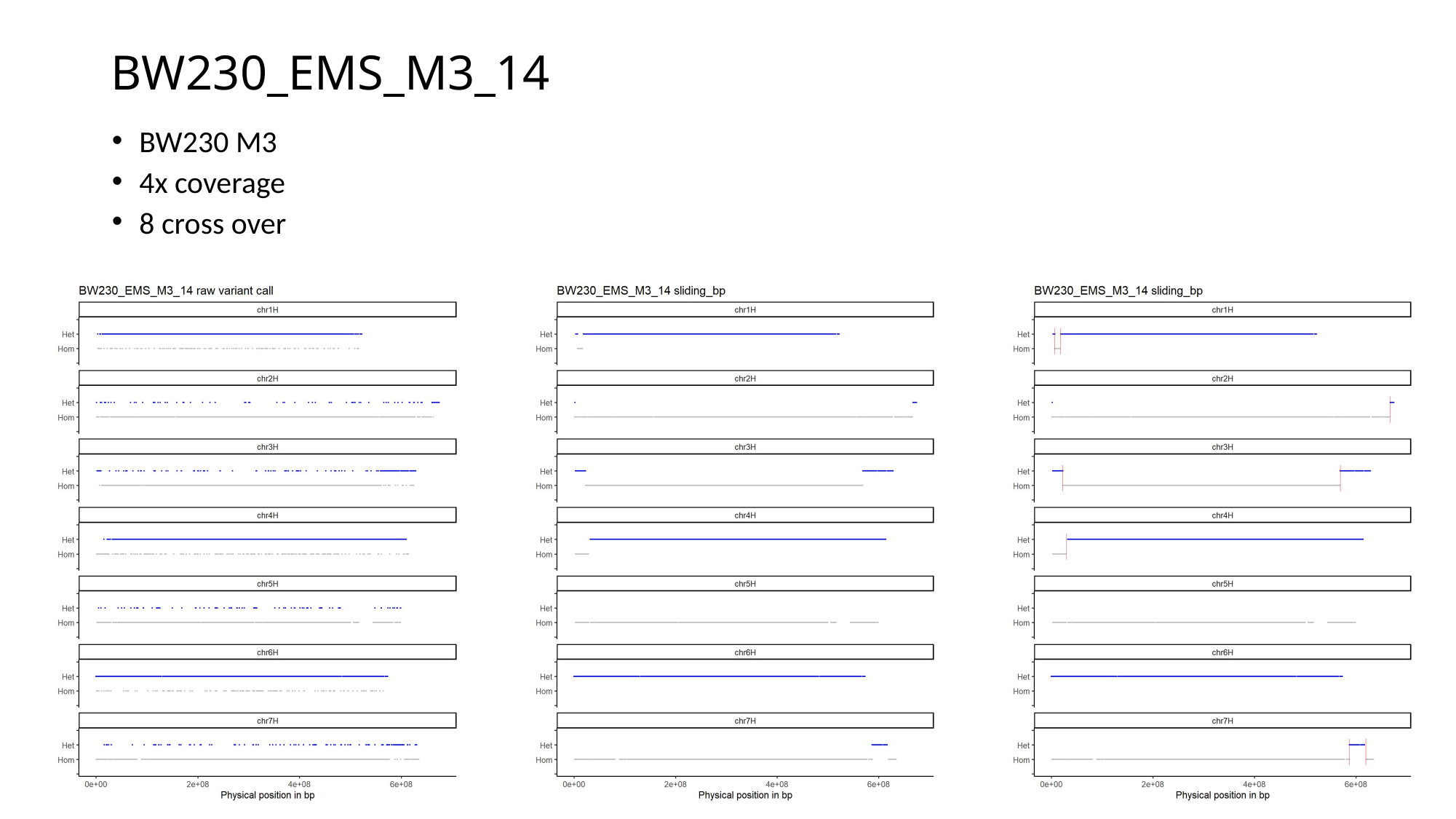

# BW230_EMS_M3_14
BW230 M3
4x coverage
8 cross over

## Slide 41
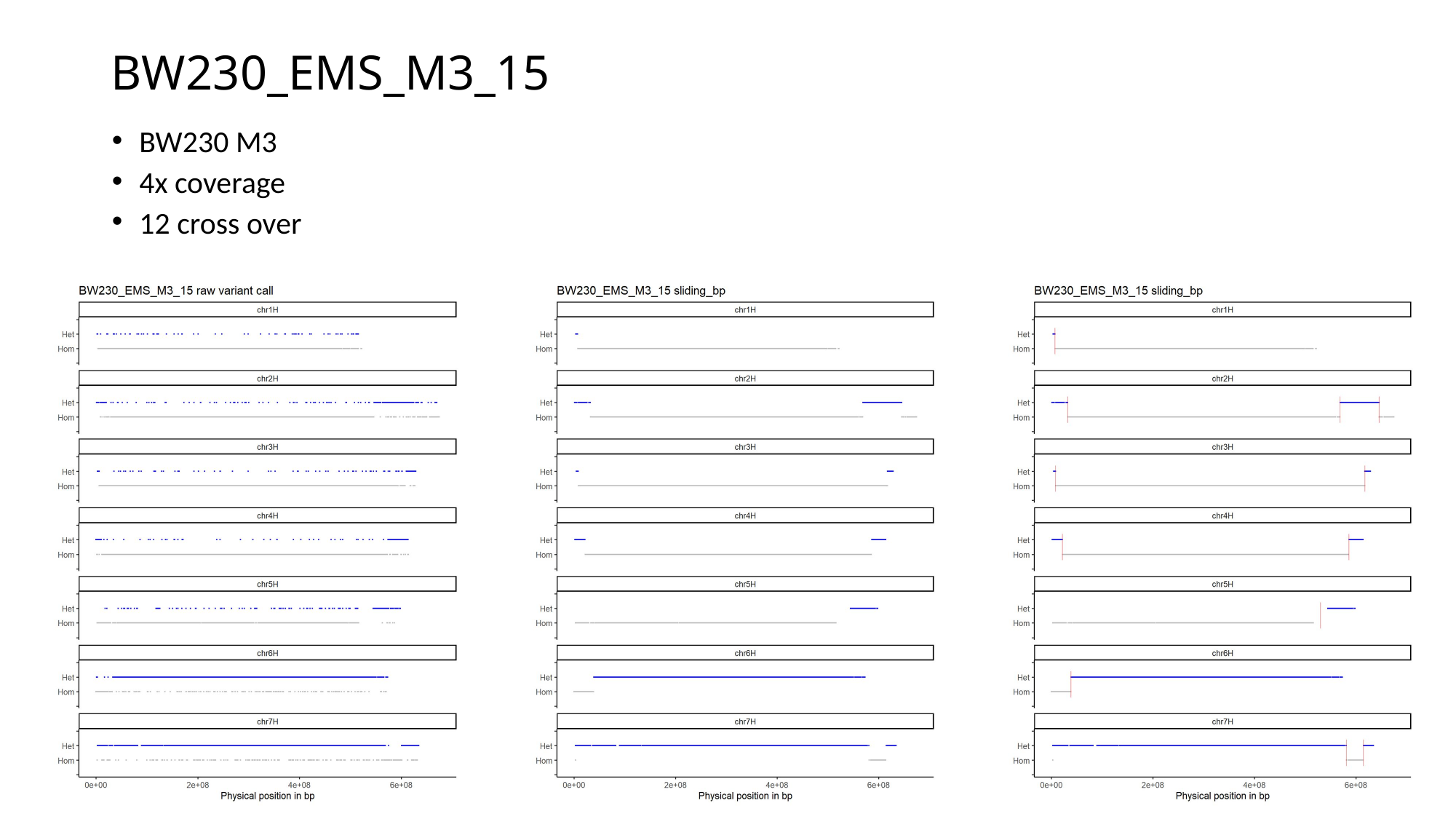

# BW230_EMS_M3_15
BW230 M3
4x coverage
12 cross over

## Slide 42
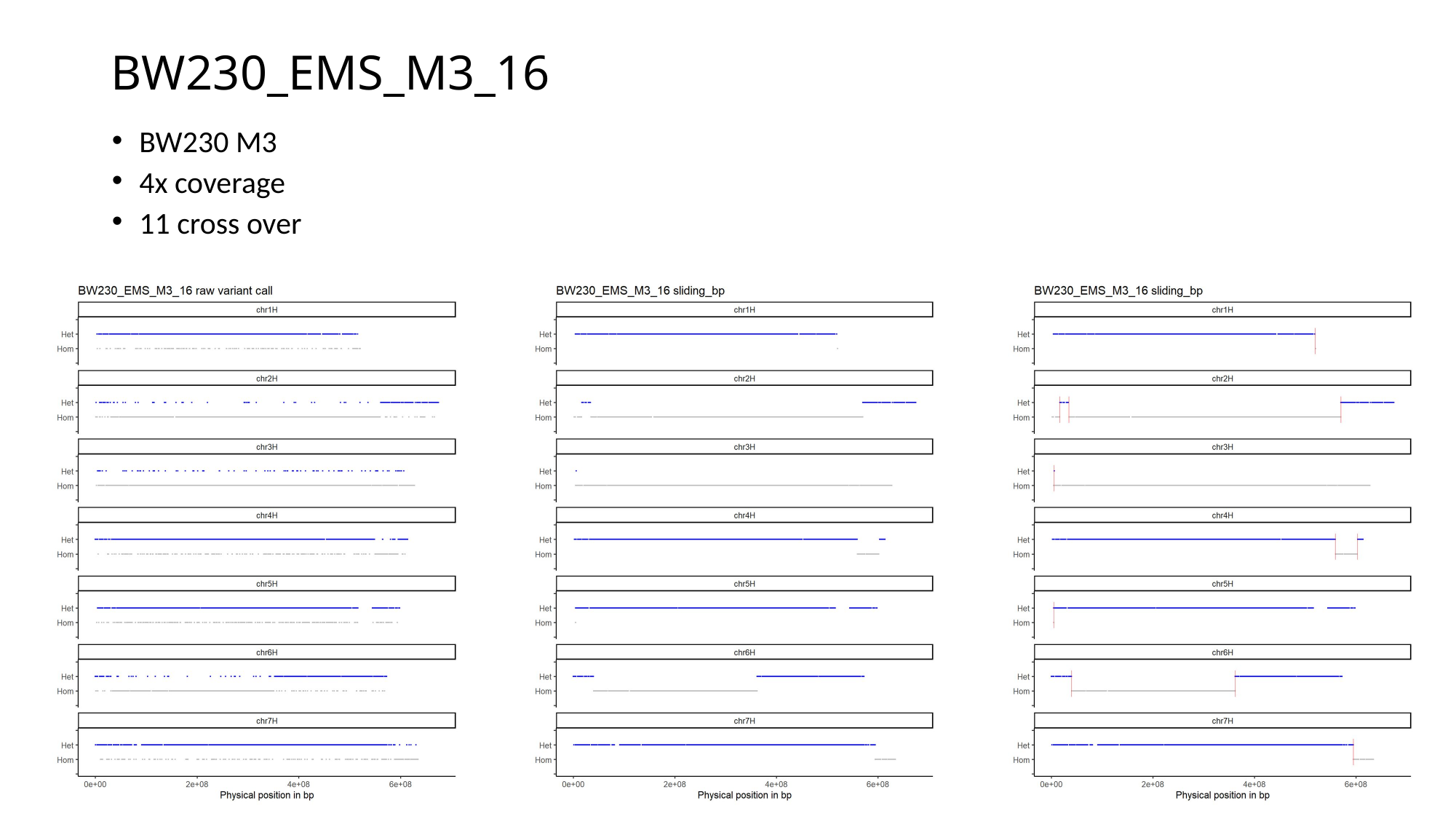

# BW230_EMS_M3_16
BW230 M3
4x coverage
11 cross over

## Slide 43
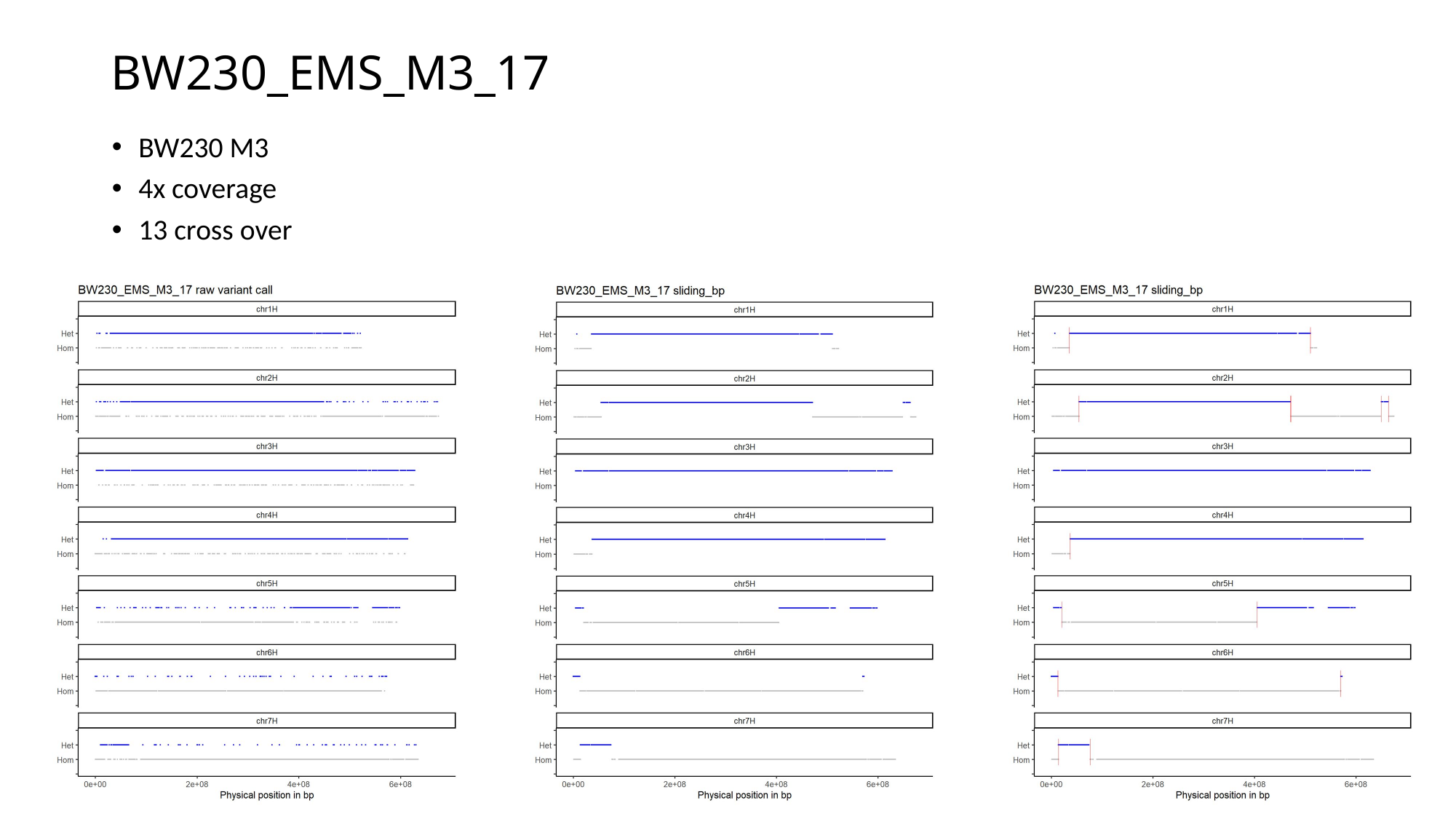

# BW230_EMS_M3_17
BW230 M3
4x coverage
13 cross over

## Slide 44
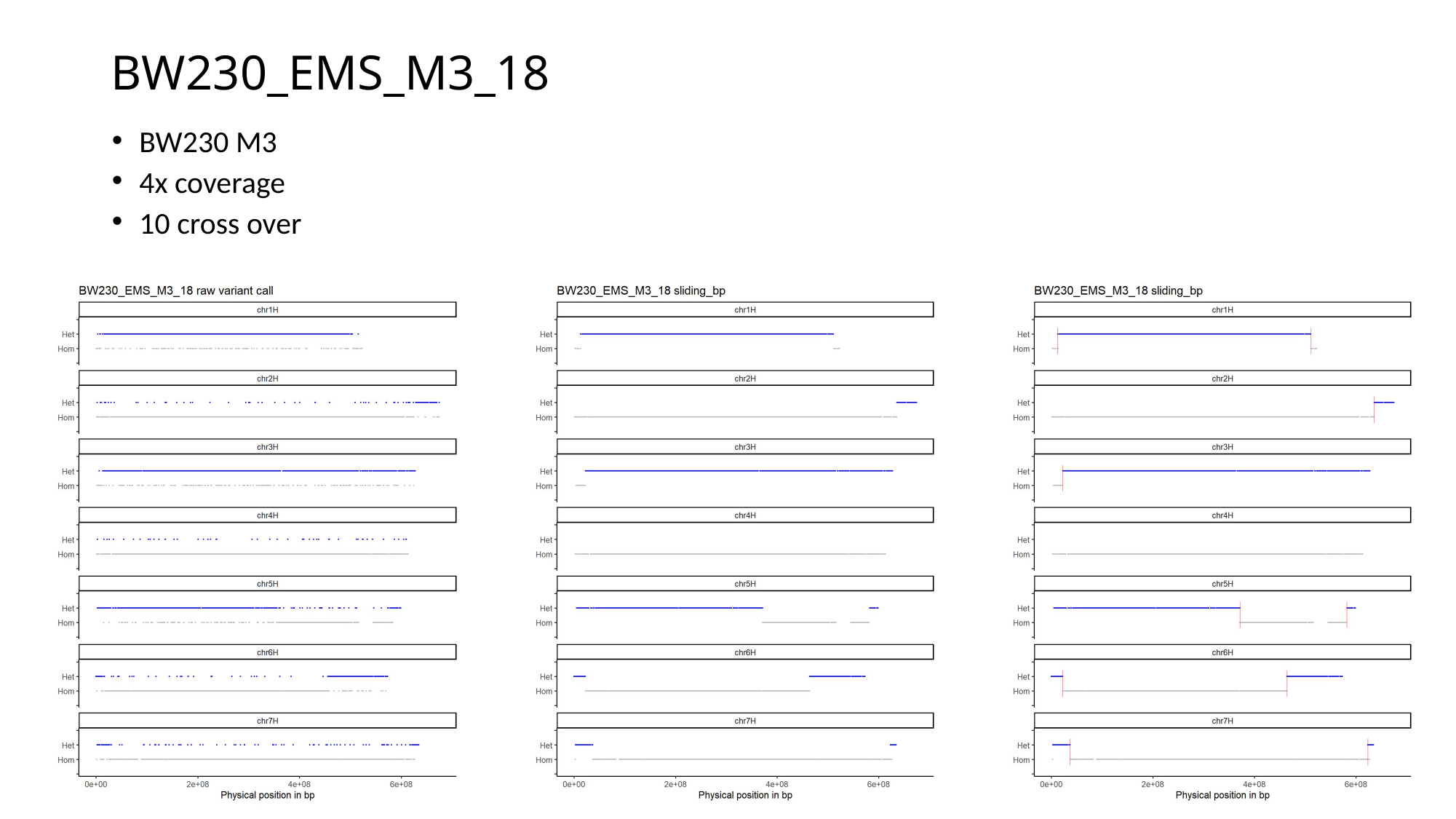

# BW230_EMS_M3_18
BW230 M3
4x coverage
10 cross over

## Slide 45
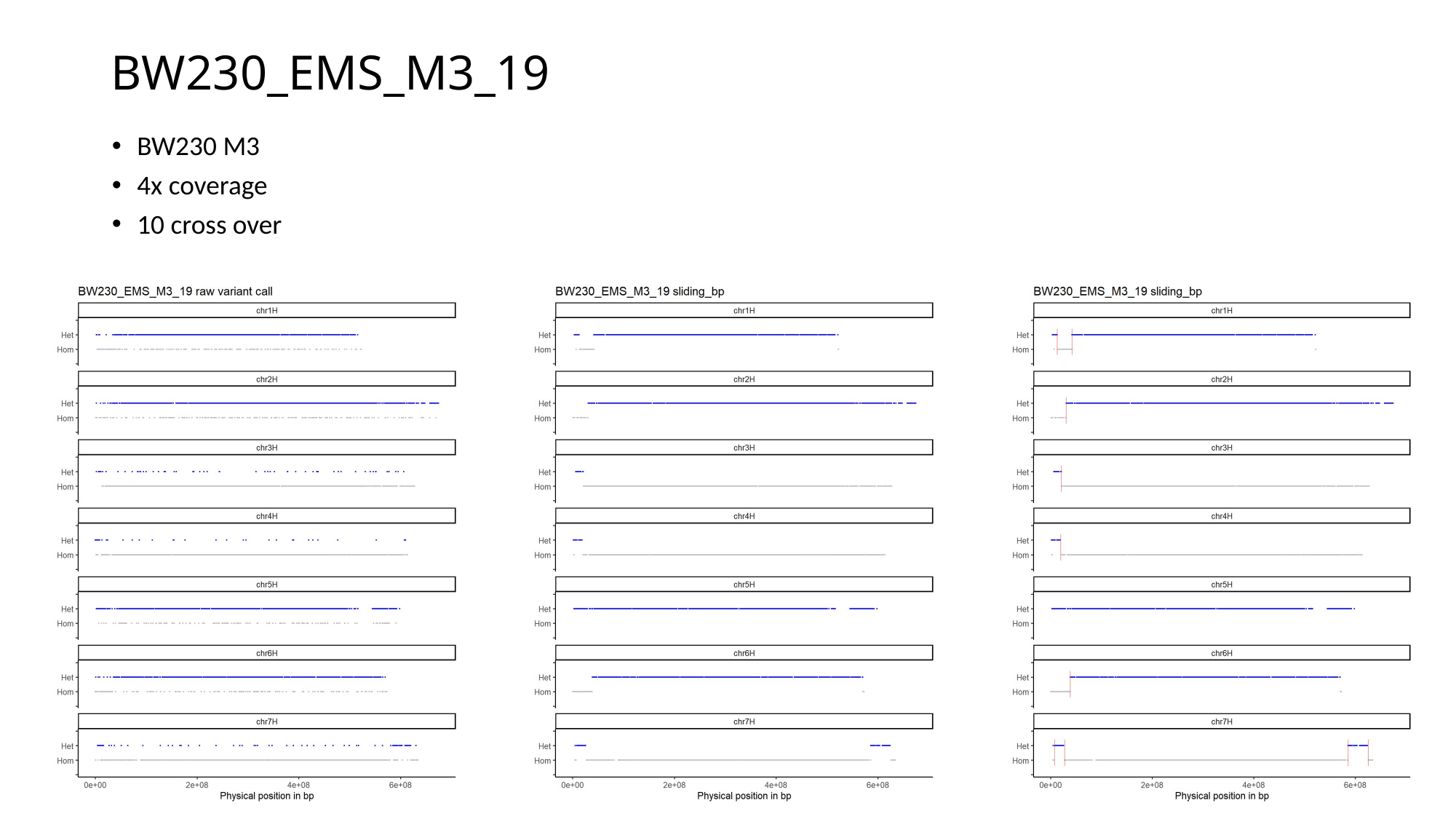

# BW230_EMS_M3_19
BW230 M3
4x coverage
10 cross over

## Slide 46
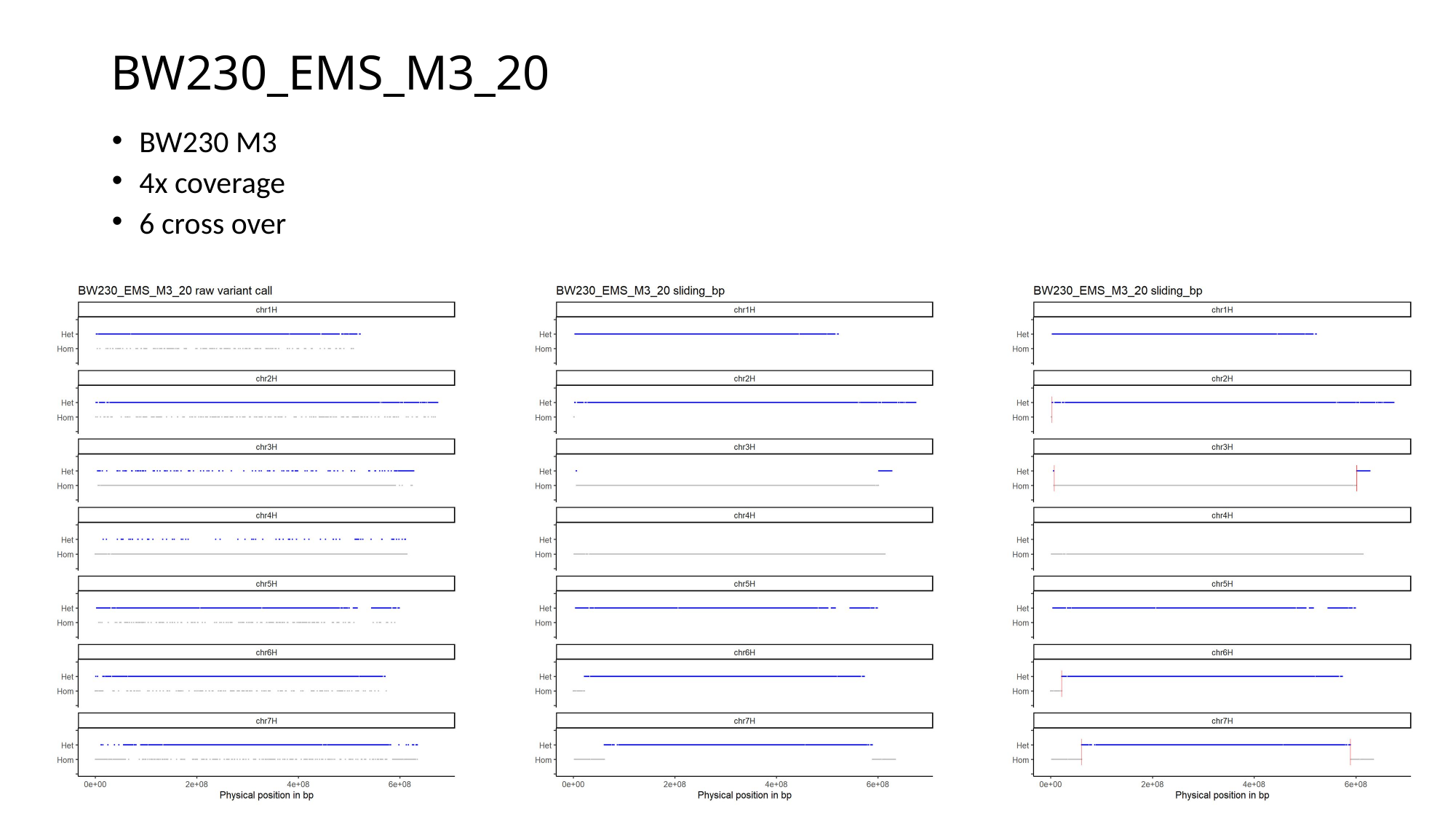

# BW230_EMS_M3_20
BW230 M3
4x coverage
6 cross over

## Slide 47
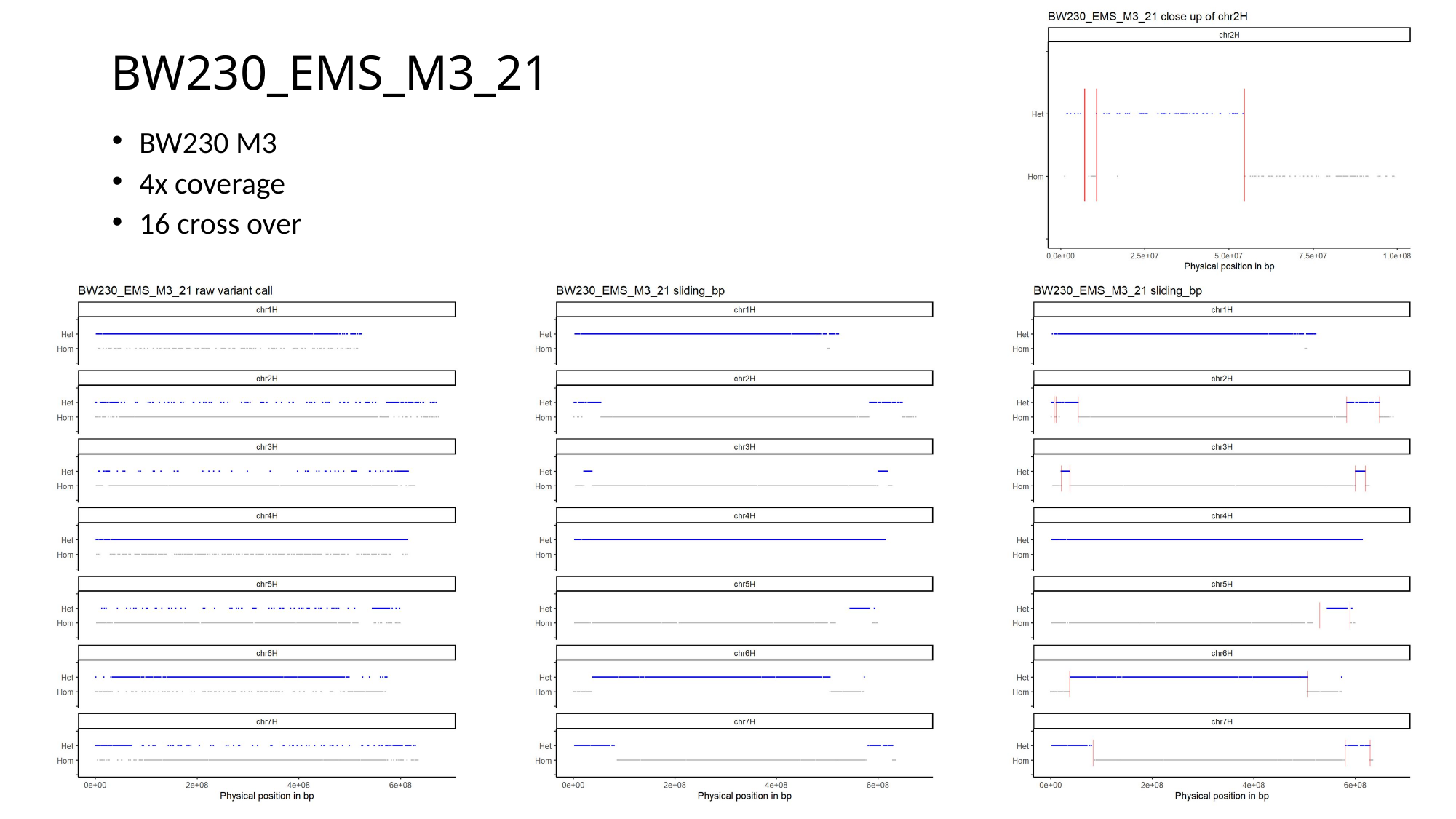

# BW230_EMS_M3_21
BW230 M3
4x coverage
16 cross over

## Slide 48
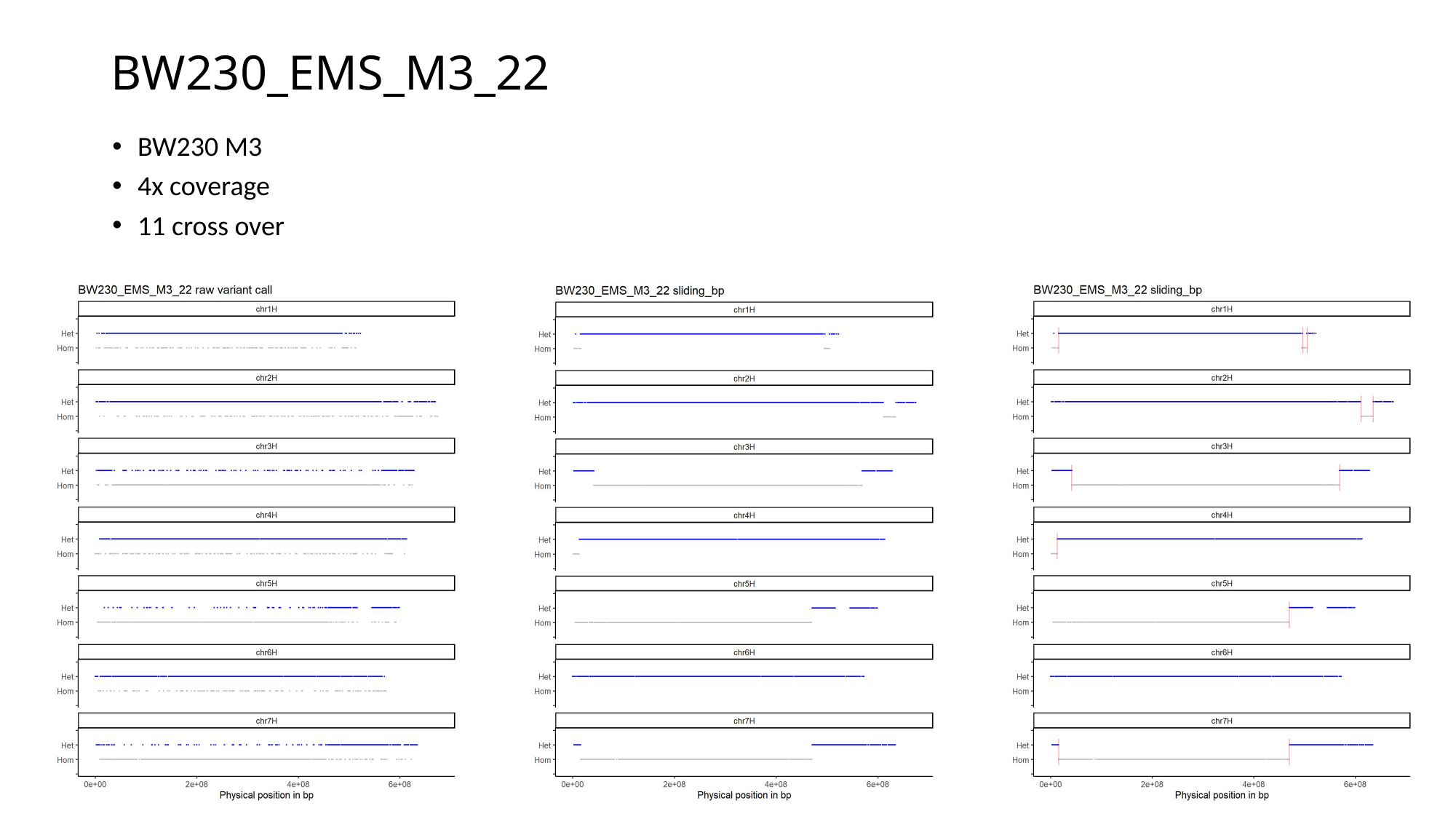

# BW230_EMS_M3_22
BW230 M3
4x coverage
11 cross over

## Slide 49
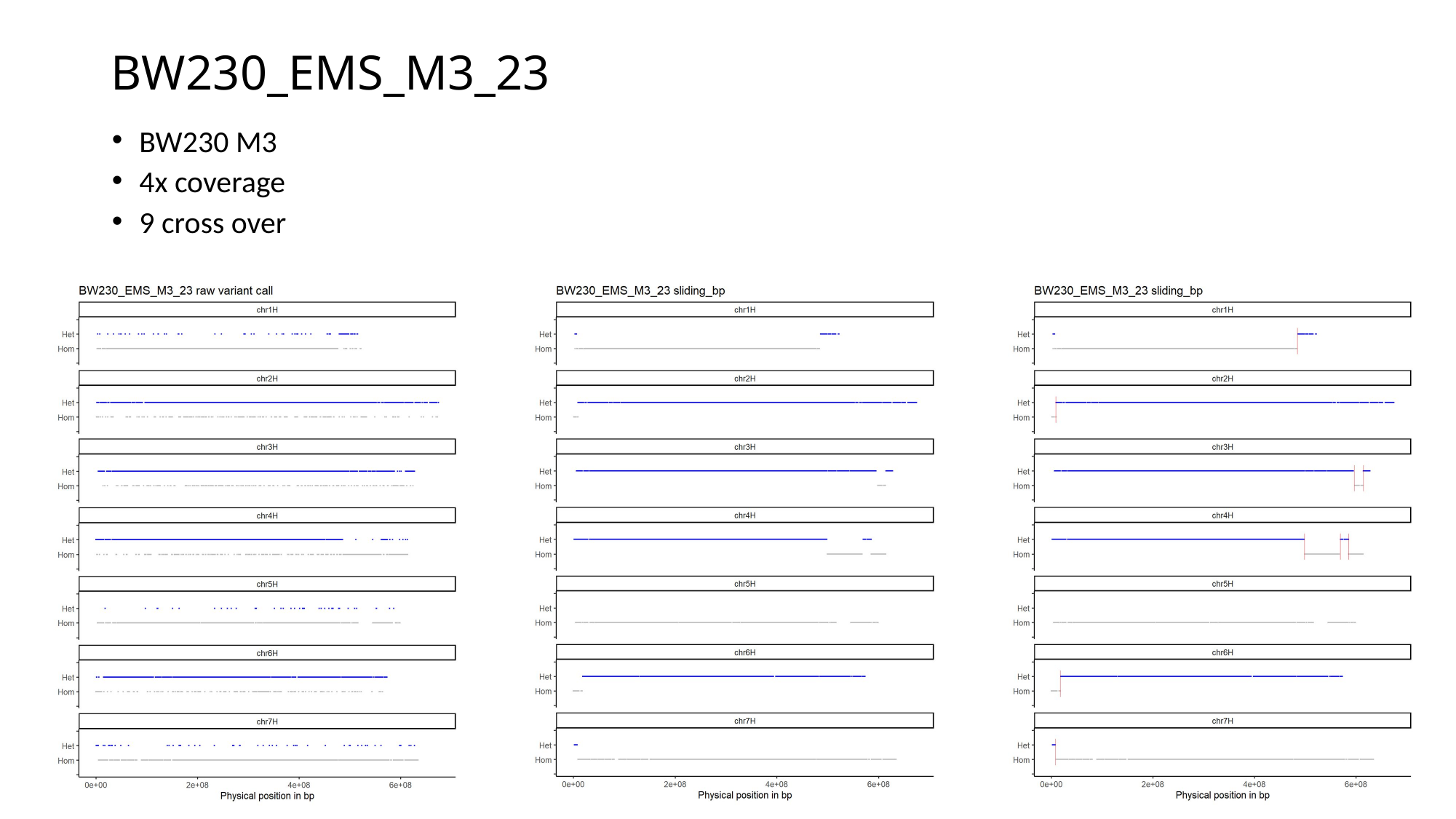

# BW230_EMS_M3_23
BW230 M3
4x coverage
9 cross over

## Slide 50
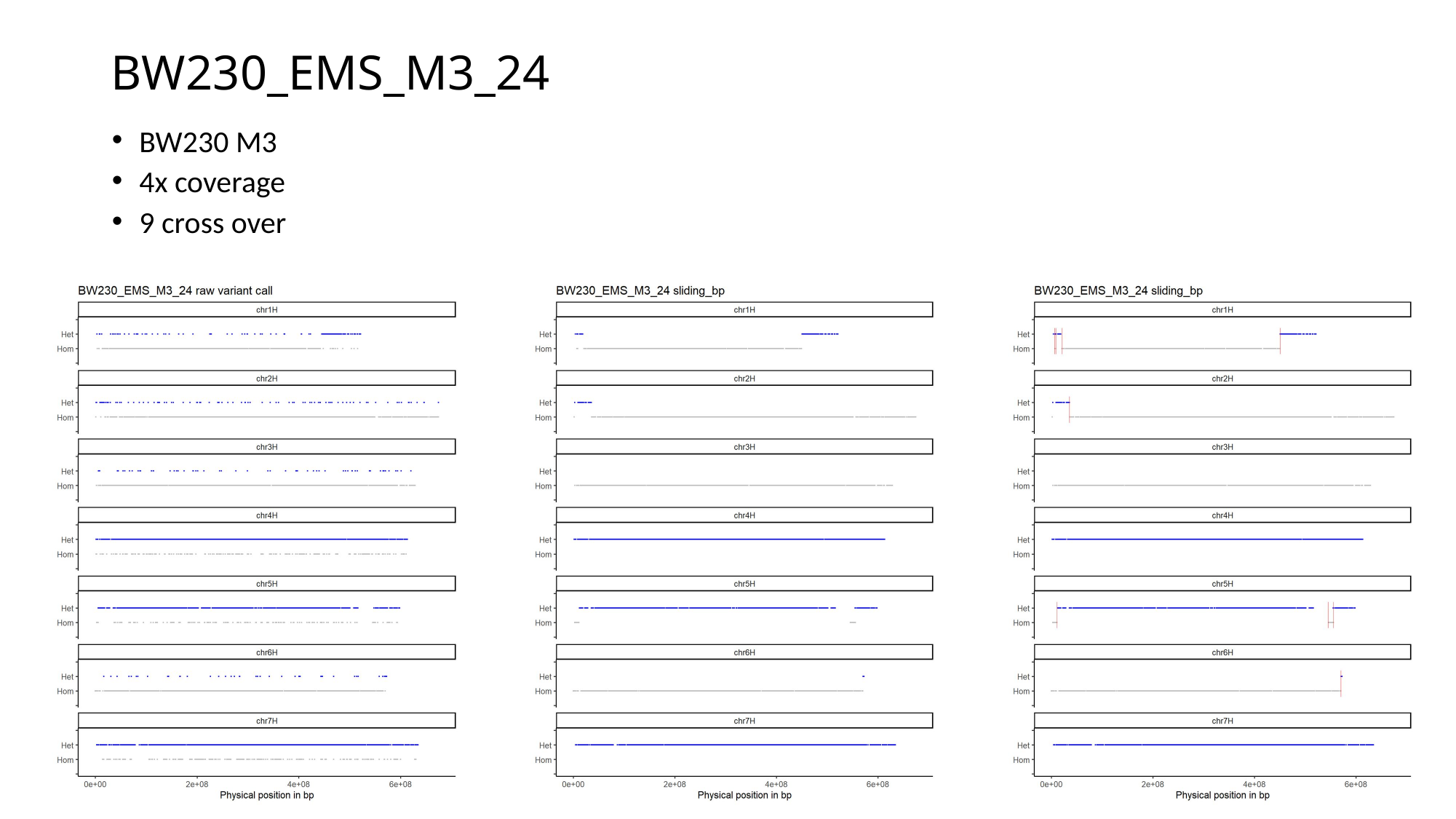

# BW230_EMS_M3_24
BW230 M3
4x coverage
9 cross over

## Slide 51
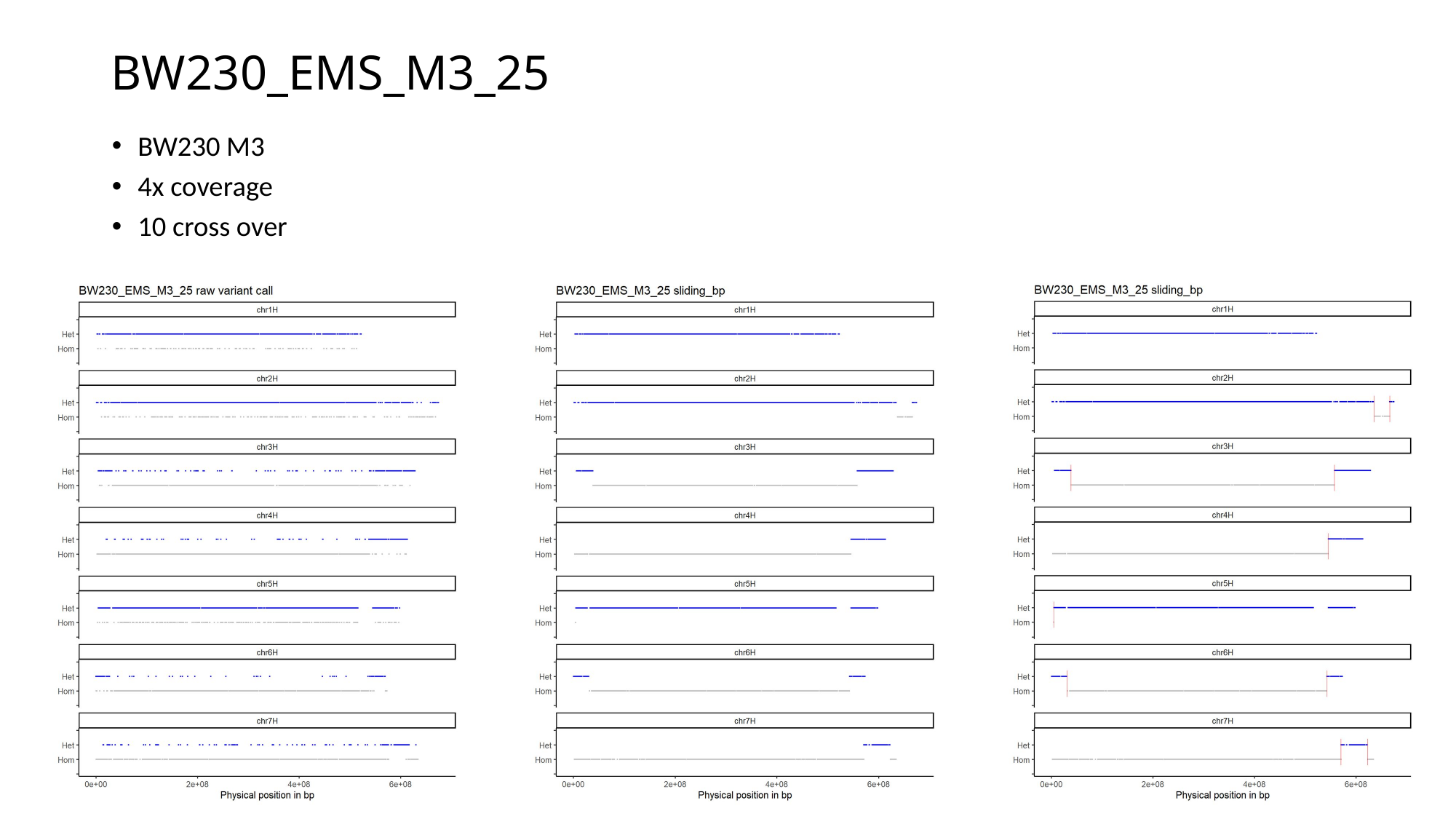

# BW230_EMS_M3_25
BW230 M3
4x coverage
10 cross over
