# Supplementary material for: Measuring the frequency and distribution of meiotic crossovers in homozygous barley inbred lines
Source: Front Plant Sci. 2022 Aug 11;13:965217. doi: 10.3389/fpls.2022.965217 (PMC9403744; doi:10.3389/fpls.2022.965217)
Supplement: Supplementary file 2 [file Presentation_2.pptx]

## Slide 1
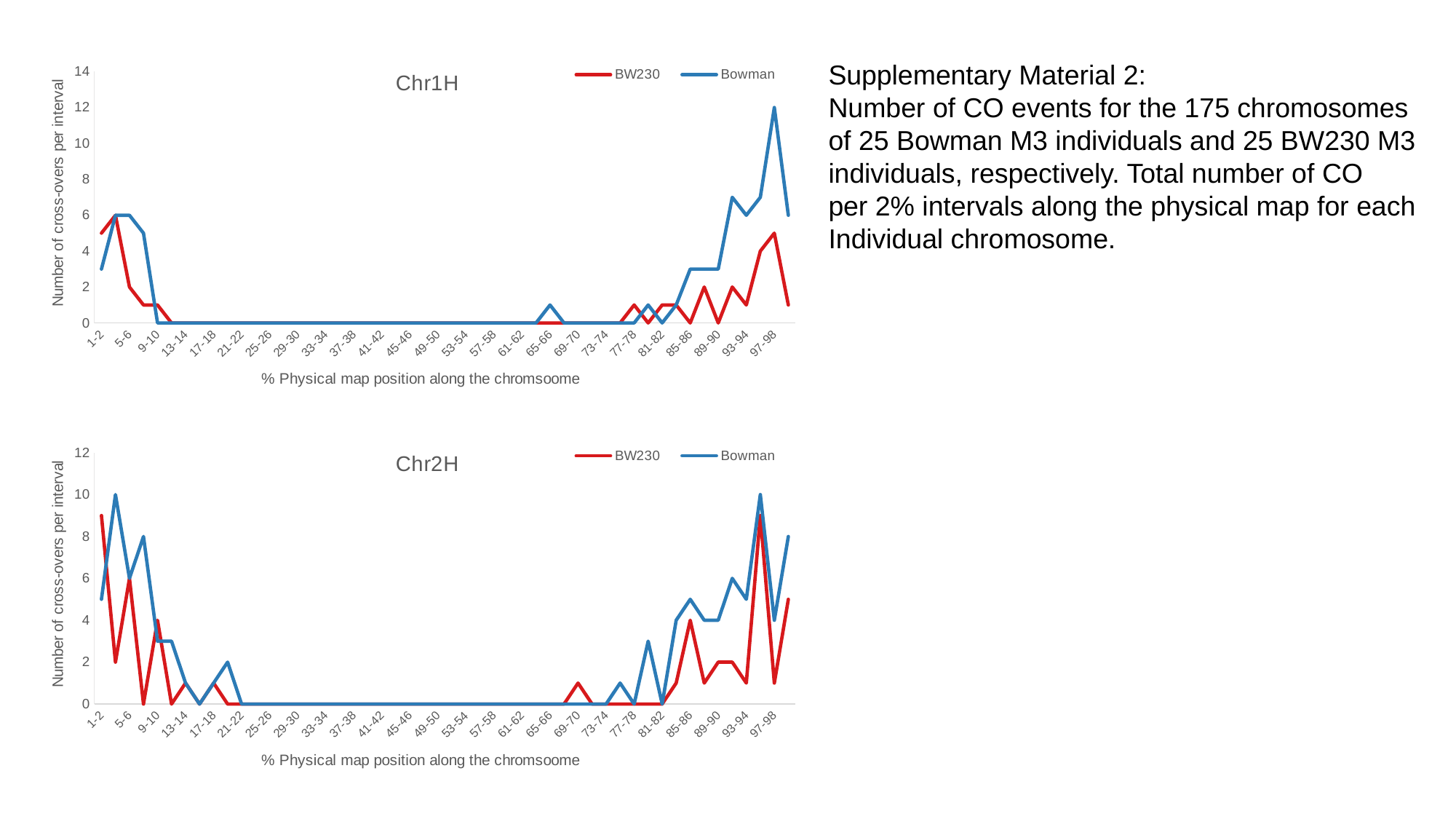

### Chart: Chr1H
| Category | BW230 | Bowman |
|---|---|---|
| 1-2 | 5.0 | 3.0 |
| 3-4 | 6.0 | 6.0 |
| 5-6 | 2.0 | 6.0 |
| 7-8 | 1.0 | 5.0 |
| 9-10 | 1.0 | 0.0 |
| 11-12 | 0.0 | 0.0 |
| 13-14 | 0.0 | 0.0 |
| 15-16 | 0.0 | 0.0 |
| 17-18 | 0.0 | 0.0 |
| 19-20 | 0.0 | 0.0 |
| 21-22 | 0.0 | 0.0 |
| 23-24 | 0.0 | 0.0 |
| 25-26 | 0.0 | 0.0 |
| 27-28 | 0.0 | 0.0 |
| 29-30 | 0.0 | 0.0 |
| 31-32 | 0.0 | 0.0 |
| 33-34 | 0.0 | 0.0 |
| 35-36 | 0.0 | 0.0 |
| 37-38 | 0.0 | 0.0 |
| 39-40 | 0.0 | 0.0 |
| 41-42 | 0.0 | 0.0 |
| 43-44 | 0.0 | 0.0 |
| 45-46 | 0.0 | 0.0 |
| 47-48 | 0.0 | 0.0 |
| 49-50 | 0.0 | 0.0 |
| 51-52 | 0.0 | 0.0 |
| 53-54 | 0.0 | 0.0 |
| 55-56 | 0.0 | 0.0 |
| 57-58 | 0.0 | 0.0 |
| 59-60 | 0.0 | 0.0 |
| 61-62 | 0.0 | 0.0 |
| 63-64 | 0.0 | 0.0 |
| 65-66 | 0.0 | 1.0 |
| 67-68 | 0.0 | 0.0 |
| 69-70 | 0.0 | 0.0 |
| 71-72 | 0.0 | 0.0 |
| 73-74 | 0.0 | 0.0 |
| 75-76 | 0.0 | 0.0 |
| 77-78 | 1.0 | 0.0 |
| 79-80 | 0.0 | 1.0 |
| 81-82 | 1.0 | 0.0 |
| 83-84 | 1.0 | 1.0 |
| 85-86 | 0.0 | 3.0 |
| 87-88 | 2.0 | 3.0 |
| 89-90 | 0.0 | 3.0 |
| 91-92 | 2.0 | 7.0 |
| 93-94 | 1.0 | 6.0 |
| 95-96 | 4.0 | 7.0 |
| 97-98 | 5.0 | 12.0 |
| 99-100 | 1.0 | 6.0 |Supplementary Material 2:
Number of CO events for the 175 chromosomes
of 25 Bowman M3 individuals and 25 BW230 M3
individuals, respectively. Total number of CO
per 2% intervals along the physical map for each
Individual chromosome.
### Chart: Chr2H
| Category | BW230 | Bowman |
|---|---|---|
| 1-2 | 9.0 | 5.0 |
| 3-4 | 2.0 | 10.0 |
| 5-6 | 6.0 | 6.0 |
| 7-8 | 0.0 | 8.0 |
| 9-10 | 4.0 | 3.0 |
| 11-12 | 0.0 | 3.0 |
| 13-14 | 1.0 | 1.0 |
| 15-16 | 0.0 | 0.0 |
| 17-18 | 1.0 | 1.0 |
| 19-20 | 0.0 | 2.0 |
| 21-22 | 0.0 | 0.0 |
| 23-24 | 0.0 | 0.0 |
| 25-26 | 0.0 | 0.0 |
| 27-28 | 0.0 | 0.0 |
| 29-30 | 0.0 | 0.0 |
| 31-32 | 0.0 | 0.0 |
| 33-34 | 0.0 | 0.0 |
| 35-36 | 0.0 | 0.0 |
| 37-38 | 0.0 | 0.0 |
| 39-40 | 0.0 | 0.0 |
| 41-42 | 0.0 | 0.0 |
| 43-44 | 0.0 | 0.0 |
| 45-46 | 0.0 | 0.0 |
| 47-48 | 0.0 | 0.0 |
| 49-50 | 0.0 | 0.0 |
| 51-52 | 0.0 | 0.0 |
| 53-54 | 0.0 | 0.0 |
| 55-56 | 0.0 | 0.0 |
| 57-58 | 0.0 | 0.0 |
| 59-60 | 0.0 | 0.0 |
| 61-62 | 0.0 | 0.0 |
| 63-64 | 0.0 | 0.0 |
| 65-66 | 0.0 | 0.0 |
| 67-68 | 0.0 | 0.0 |
| 69-70 | 1.0 | 0.0 |
| 71-72 | 0.0 | 0.0 |
| 73-74 | 0.0 | 0.0 |
| 75-76 | 0.0 | 1.0 |
| 77-78 | 0.0 | 0.0 |
| 79-80 | 0.0 | 3.0 |
| 81-82 | 0.0 | 0.0 |
| 83-84 | 1.0 | 4.0 |
| 85-86 | 4.0 | 5.0 |
| 87-88 | 1.0 | 4.0 |
| 89-90 | 2.0 | 4.0 |
| 91-92 | 2.0 | 6.0 |
| 93-94 | 1.0 | 5.0 |
| 95-96 | 9.0 | 10.0 |
| 97-98 | 1.0 | 4.0 |
| 99-100 | 5.0 | 8.0 |

## Slide 2
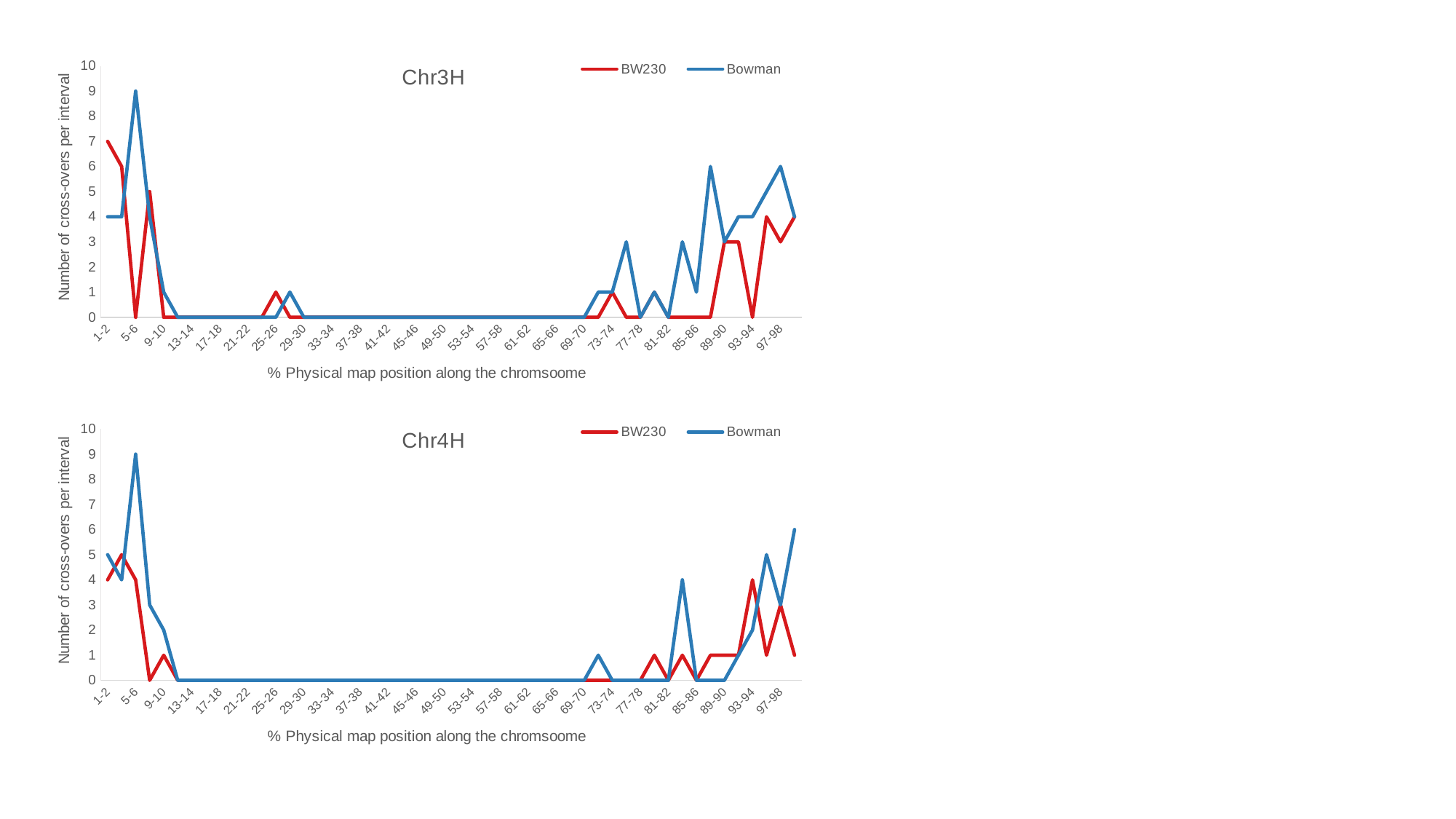

### Chart: Chr3H
| Category | BW230 | Bowman |
|---|---|---|
| 1-2 | 7.0 | 4.0 |
| 3-4 | 6.0 | 4.0 |
| 5-6 | 0.0 | 9.0 |
| 7-8 | 5.0 | 4.0 |
| 9-10 | 0.0 | 1.0 |
| 11-12 | 0.0 | 0.0 |
| 13-14 | 0.0 | 0.0 |
| 15-16 | 0.0 | 0.0 |
| 17-18 | 0.0 | 0.0 |
| 19-20 | 0.0 | 0.0 |
| 21-22 | 0.0 | 0.0 |
| 23-24 | 0.0 | 0.0 |
| 25-26 | 1.0 | 0.0 |
| 27-28 | 0.0 | 1.0 |
| 29-30 | 0.0 | 0.0 |
| 31-32 | 0.0 | 0.0 |
| 33-34 | 0.0 | 0.0 |
| 35-36 | 0.0 | 0.0 |
| 37-38 | 0.0 | 0.0 |
| 39-40 | 0.0 | 0.0 |
| 41-42 | 0.0 | 0.0 |
| 43-44 | 0.0 | 0.0 |
| 45-46 | 0.0 | 0.0 |
| 47-48 | 0.0 | 0.0 |
| 49-50 | 0.0 | 0.0 |
| 51-52 | 0.0 | 0.0 |
| 53-54 | 0.0 | 0.0 |
| 55-56 | 0.0 | 0.0 |
| 57-58 | 0.0 | 0.0 |
| 59-60 | 0.0 | 0.0 |
| 61-62 | 0.0 | 0.0 |
| 63-64 | 0.0 | 0.0 |
| 65-66 | 0.0 | 0.0 |
| 67-68 | 0.0 | 0.0 |
| 69-70 | 0.0 | 0.0 |
| 71-72 | 0.0 | 1.0 |
| 73-74 | 1.0 | 1.0 |
| 75-76 | 0.0 | 3.0 |
| 77-78 | 0.0 | 0.0 |
| 79-80 | 1.0 | 1.0 |
| 81-82 | 0.0 | 0.0 |
| 83-84 | 0.0 | 3.0 |
| 85-86 | 0.0 | 1.0 |
| 87-88 | 0.0 | 6.0 |
| 89-90 | 3.0 | 3.0 |
| 91-92 | 3.0 | 4.0 |
| 93-94 | 0.0 | 4.0 |
| 95-96 | 4.0 | 5.0 |
| 97-98 | 3.0 | 6.0 |
| 99-100 | 4.0 | 4.0 |
### Chart: Chr4H
| Category | BW230 | Bowman |
|---|---|---|
| 1-2 | 4.0 | 5.0 |
| 3-4 | 5.0 | 4.0 |
| 5-6 | 4.0 | 9.0 |
| 7-8 | 0.0 | 3.0 |
| 9-10 | 1.0 | 2.0 |
| 11-12 | 0.0 | 0.0 |
| 13-14 | 0.0 | 0.0 |
| 15-16 | 0.0 | 0.0 |
| 17-18 | 0.0 | 0.0 |
| 19-20 | 0.0 | 0.0 |
| 21-22 | 0.0 | 0.0 |
| 23-24 | 0.0 | 0.0 |
| 25-26 | 0.0 | 0.0 |
| 27-28 | 0.0 | 0.0 |
| 29-30 | 0.0 | 0.0 |
| 31-32 | 0.0 | 0.0 |
| 33-34 | 0.0 | 0.0 |
| 35-36 | 0.0 | 0.0 |
| 37-38 | 0.0 | 0.0 |
| 39-40 | 0.0 | 0.0 |
| 41-42 | 0.0 | 0.0 |
| 43-44 | 0.0 | 0.0 |
| 45-46 | 0.0 | 0.0 |
| 47-48 | 0.0 | 0.0 |
| 49-50 | 0.0 | 0.0 |
| 51-52 | 0.0 | 0.0 |
| 53-54 | 0.0 | 0.0 |
| 55-56 | 0.0 | 0.0 |
| 57-58 | 0.0 | 0.0 |
| 59-60 | 0.0 | 0.0 |
| 61-62 | 0.0 | 0.0 |
| 63-64 | 0.0 | 0.0 |
| 65-66 | 0.0 | 0.0 |
| 67-68 | 0.0 | 0.0 |
| 69-70 | 0.0 | 0.0 |
| 71-72 | 0.0 | 1.0 |
| 73-74 | 0.0 | 0.0 |
| 75-76 | 0.0 | 0.0 |
| 77-78 | 0.0 | 0.0 |
| 79-80 | 1.0 | 0.0 |
| 81-82 | 0.0 | 0.0 |
| 83-84 | 1.0 | 4.0 |
| 85-86 | 0.0 | 0.0 |
| 87-88 | 1.0 | 0.0 |
| 89-90 | 1.0 | 0.0 |
| 91-92 | 1.0 | 1.0 |
| 93-94 | 4.0 | 2.0 |
| 95-96 | 1.0 | 5.0 |
| 97-98 | 3.0 | 3.0 |
| 99-100 | 1.0 | 6.0 |

## Slide 3
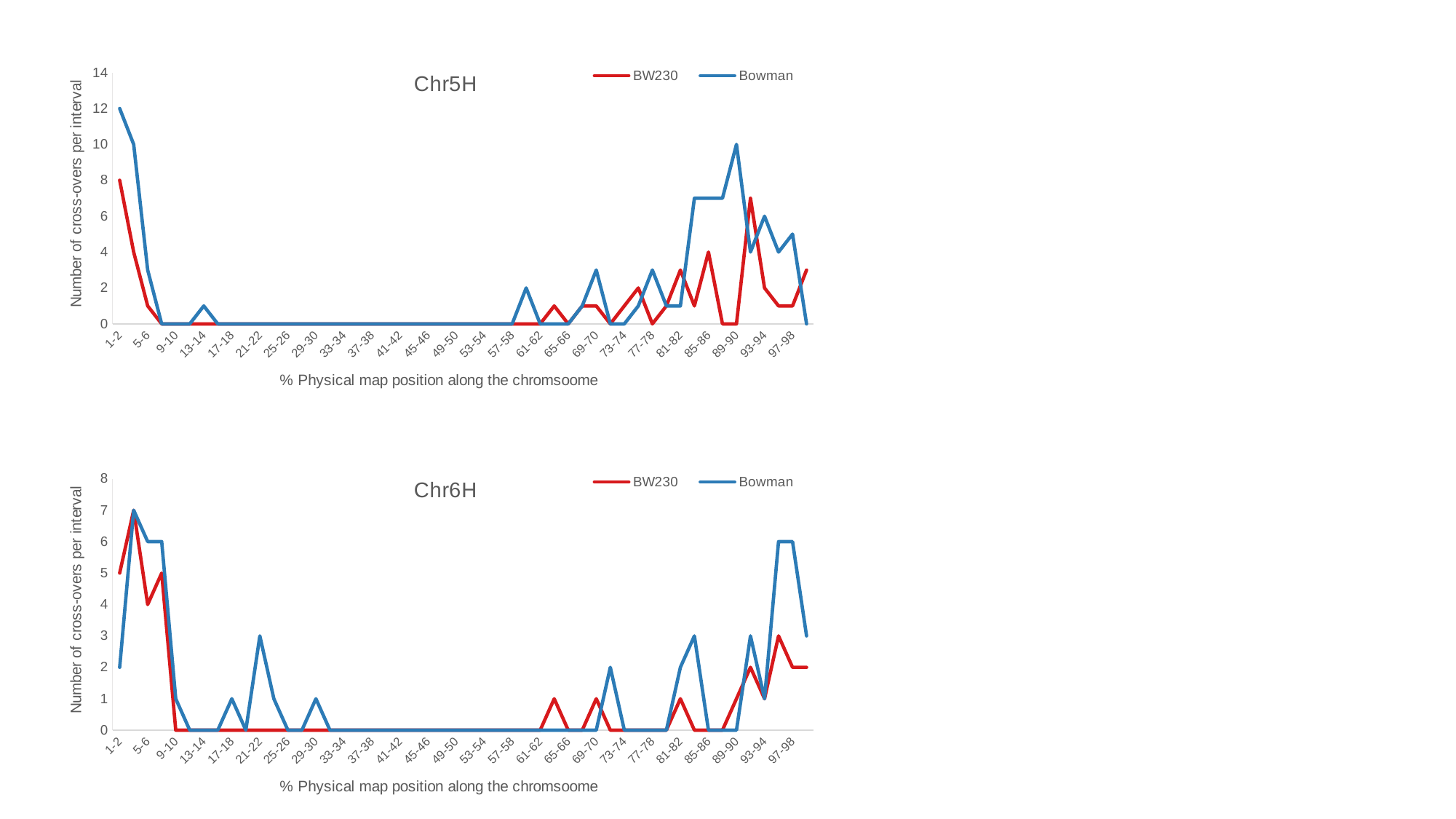

### Chart: Chr5H
| Category | BW230 | Bowman |
|---|---|---|
| 1-2 | 8.0 | 12.0 |
| 3-4 | 4.0 | 10.0 |
| 5-6 | 1.0 | 3.0 |
| 7-8 | 0.0 | 0.0 |
| 9-10 | 0.0 | 0.0 |
| 11-12 | 0.0 | 0.0 |
| 13-14 | 0.0 | 1.0 |
| 15-16 | 0.0 | 0.0 |
| 17-18 | 0.0 | 0.0 |
| 19-20 | 0.0 | 0.0 |
| 21-22 | 0.0 | 0.0 |
| 23-24 | 0.0 | 0.0 |
| 25-26 | 0.0 | 0.0 |
| 27-28 | 0.0 | 0.0 |
| 29-30 | 0.0 | 0.0 |
| 31-32 | 0.0 | 0.0 |
| 33-34 | 0.0 | 0.0 |
| 35-36 | 0.0 | 0.0 |
| 37-38 | 0.0 | 0.0 |
| 39-40 | 0.0 | 0.0 |
| 41-42 | 0.0 | 0.0 |
| 43-44 | 0.0 | 0.0 |
| 45-46 | 0.0 | 0.0 |
| 47-48 | 0.0 | 0.0 |
| 49-50 | 0.0 | 0.0 |
| 51-52 | 0.0 | 0.0 |
| 53-54 | 0.0 | 0.0 |
| 55-56 | 0.0 | 0.0 |
| 57-58 | 0.0 | 0.0 |
| 59-60 | 0.0 | 2.0 |
| 61-62 | 0.0 | 0.0 |
| 63-64 | 1.0 | 0.0 |
| 65-66 | 0.0 | 0.0 |
| 67-68 | 1.0 | 1.0 |
| 69-70 | 1.0 | 3.0 |
| 71-72 | 0.0 | 0.0 |
| 73-74 | 1.0 | 0.0 |
| 75-76 | 2.0 | 1.0 |
| 77-78 | 0.0 | 3.0 |
| 79-80 | 1.0 | 1.0 |
| 81-82 | 3.0 | 1.0 |
| 83-84 | 1.0 | 7.0 |
| 85-86 | 4.0 | 7.0 |
| 87-88 | 0.0 | 7.0 |
| 89-90 | 0.0 | 10.0 |
| 91-92 | 7.0 | 4.0 |
| 93-94 | 2.0 | 6.0 |
| 95-96 | 1.0 | 4.0 |
| 97-98 | 1.0 | 5.0 |
| 99-100 | 3.0 | 0.0 |
### Chart: Chr6H
| Category | BW230 | Bowman |
|---|---|---|
| 1-2 | 5.0 | 2.0 |
| 3-4 | 7.0 | 7.0 |
| 5-6 | 4.0 | 6.0 |
| 7-8 | 5.0 | 6.0 |
| 9-10 | 0.0 | 1.0 |
| 11-12 | 0.0 | 0.0 |
| 13-14 | 0.0 | 0.0 |
| 15-16 | 0.0 | 0.0 |
| 17-18 | 0.0 | 1.0 |
| 19-20 | 0.0 | 0.0 |
| 21-22 | 0.0 | 3.0 |
| 23-24 | 0.0 | 1.0 |
| 25-26 | 0.0 | 0.0 |
| 27-28 | 0.0 | 0.0 |
| 29-30 | 0.0 | 1.0 |
| 31-32 | 0.0 | 0.0 |
| 33-34 | 0.0 | 0.0 |
| 35-36 | 0.0 | 0.0 |
| 37-38 | 0.0 | 0.0 |
| 39-40 | 0.0 | 0.0 |
| 41-42 | 0.0 | 0.0 |
| 43-44 | 0.0 | 0.0 |
| 45-46 | 0.0 | 0.0 |
| 47-48 | 0.0 | 0.0 |
| 49-50 | 0.0 | 0.0 |
| 51-52 | 0.0 | 0.0 |
| 53-54 | 0.0 | 0.0 |
| 55-56 | 0.0 | 0.0 |
| 57-58 | 0.0 | 0.0 |
| 59-60 | 0.0 | 0.0 |
| 61-62 | 0.0 | 0.0 |
| 63-64 | 1.0 | 0.0 |
| 65-66 | 0.0 | 0.0 |
| 67-68 | 0.0 | 0.0 |
| 69-70 | 1.0 | 0.0 |
| 71-72 | 0.0 | 2.0 |
| 73-74 | 0.0 | 0.0 |
| 75-76 | 0.0 | 0.0 |
| 77-78 | 0.0 | 0.0 |
| 79-80 | 0.0 | 0.0 |
| 81-82 | 1.0 | 2.0 |
| 83-84 | 0.0 | 3.0 |
| 85-86 | 0.0 | 0.0 |
| 87-88 | 0.0 | 0.0 |
| 89-90 | 1.0 | 0.0 |
| 91-92 | 2.0 | 3.0 |
| 93-94 | 1.0 | 1.0 |
| 95-96 | 3.0 | 6.0 |
| 97-98 | 2.0 | 6.0 |
| 99-100 | 2.0 | 3.0 |

## Slide 4
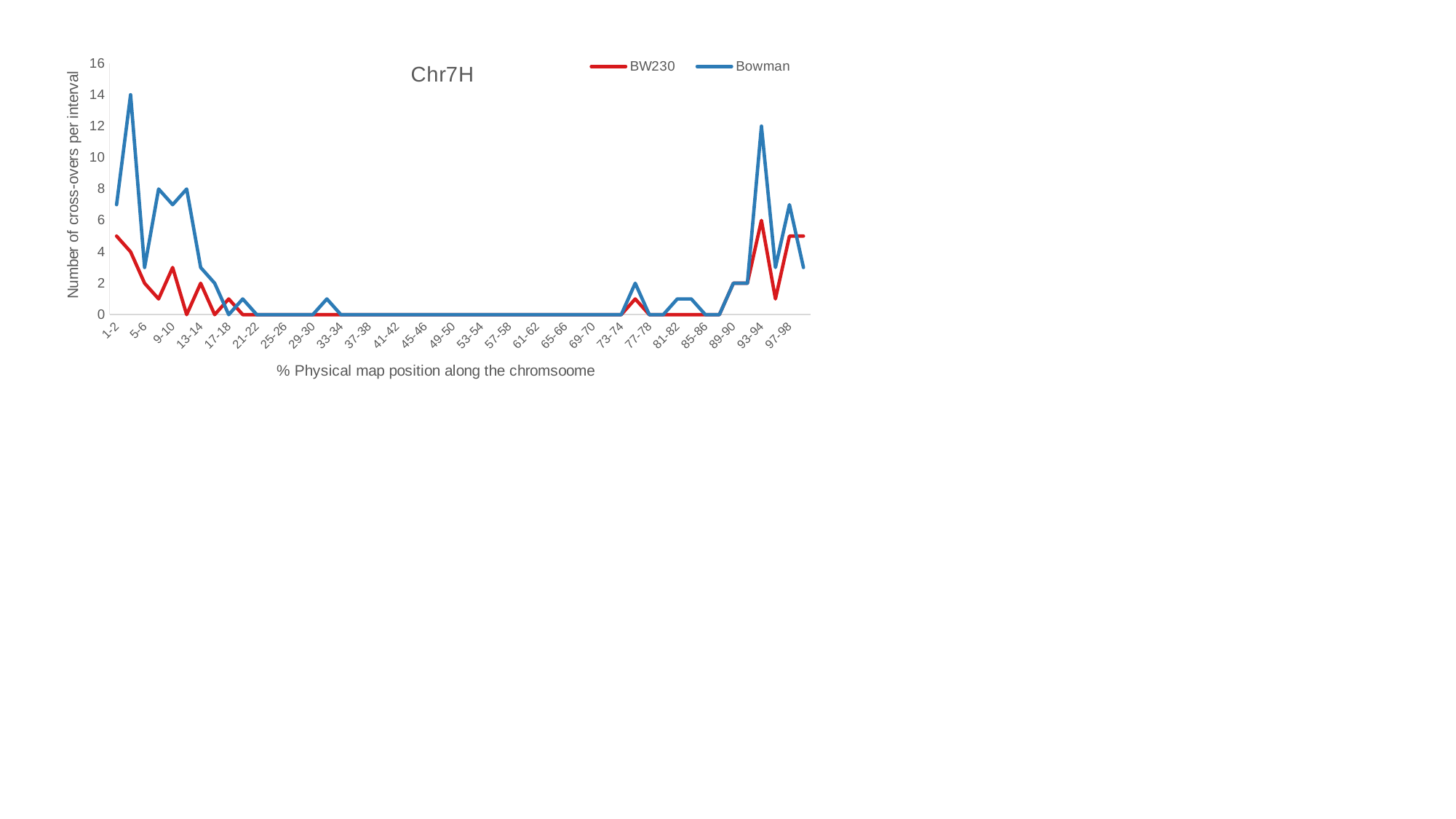

### Chart: Chr7H
| Category | BW230 | Bowman |
|---|---|---|
| 1-2 | 5.0 | 7.0 |
| 3-4 | 4.0 | 14.0 |
| 5-6 | 2.0 | 3.0 |
| 7-8 | 1.0 | 8.0 |
| 9-10 | 3.0 | 7.0 |
| 11-12 | 0.0 | 8.0 |
| 13-14 | 2.0 | 3.0 |
| 15-16 | 0.0 | 2.0 |
| 17-18 | 1.0 | 0.0 |
| 19-20 | 0.0 | 1.0 |
| 21-22 | 0.0 | 0.0 |
| 23-24 | 0.0 | 0.0 |
| 25-26 | 0.0 | 0.0 |
| 27-28 | 0.0 | 0.0 |
| 29-30 | 0.0 | 0.0 |
| 31-32 | 0.0 | 1.0 |
| 33-34 | 0.0 | 0.0 |
| 35-36 | 0.0 | 0.0 |
| 37-38 | 0.0 | 0.0 |
| 39-40 | 0.0 | 0.0 |
| 41-42 | 0.0 | 0.0 |
| 43-44 | 0.0 | 0.0 |
| 45-46 | 0.0 | 0.0 |
| 47-48 | 0.0 | 0.0 |
| 49-50 | 0.0 | 0.0 |
| 51-52 | 0.0 | 0.0 |
| 53-54 | 0.0 | 0.0 |
| 55-56 | 0.0 | 0.0 |
| 57-58 | 0.0 | 0.0 |
| 59-60 | 0.0 | 0.0 |
| 61-62 | 0.0 | 0.0 |
| 63-64 | 0.0 | 0.0 |
| 65-66 | 0.0 | 0.0 |
| 67-68 | 0.0 | 0.0 |
| 69-70 | 0.0 | 0.0 |
| 71-72 | 0.0 | 0.0 |
| 73-74 | 0.0 | 0.0 |
| 75-76 | 1.0 | 2.0 |
| 77-78 | 0.0 | 0.0 |
| 79-80 | 0.0 | 0.0 |
| 81-82 | 0.0 | 1.0 |
| 83-84 | 0.0 | 1.0 |
| 85-86 | 0.0 | 0.0 |
| 87-88 | 0.0 | 0.0 |
| 89-90 | 2.0 | 2.0 |
| 91-92 | 2.0 | 2.0 |
| 93-94 | 6.0 | 12.0 |
| 95-96 | 1.0 | 3.0 |
| 97-98 | 5.0 | 7.0 |
| 99-100 | 5.0 | 3.0 |
