# Supplementary material for: Measuring the frequency and distribution of meiotic crossovers in homozygous barley inbred lines
Source: Front Plant Sci. 2022 Aug 11;13:965217. doi: 10.3389/fpls.2022.965217 (PMC9403744; doi:10.3389/fpls.2022.965217)
Supplement: Supplementary file 3 [file Table_1.DOCX]

Supplemental Table 1: Genes with predicted deleterious amino acid changes by PROVEAN in the M2 plants. All identified variants were heterozygous. Gene confidence is split into low confidence (LC) and high confidence (HC) classes.

| **Gene** | **Position** | **Gene confidence class** | **Description** | **M2 Plant** | **PROVEAN Score** |
| --- | --- | --- | --- | --- | --- |
| HORVU.MOREX.r2.1HG0030920 | chr1H:250694835-250698991 | HC | Polyadenylate-binding protein | Bowman | -4.448 |
| HORVU.MOREX.r2.1HG0038690 | chr1H:329951945-329952370 | HC | Retrotransposon protein, putative, unclassified | Bowman | -6.3 |
| HORVU.MOREX.r2.1HG0048830 | chr1H:409839289-409841198 | HC | D-3-phosphoglycerate dehydrogenase | Bowman | -5.859 |
| HORVU.MOREX.r2.1HG0068090 | chr1H:496105330-496107477 | HC | Alcohol dehydrogenase | Bowman | -5.414 |
| HORVU.MOREX.r2.2HG0083310 | chr2H:11386832-11390307 | HC | Disease resistance protein | Bowman | -9.288 |
| HORVU.MOREX.r2.2HG0108870 | chr2H:145237539-145238525 | LC | Amine oxidase, putative | Bowman | -5.057 |
| HORVU.MOREX.r2.2HG0125800 | chr2H:358041591-358045117 | HC | Receptor-like kinase | Bowman | -7.879 |
| HORVU.MOREX.r2.2HG0130800 | chr2H:406423885-406427200 | LC | Basic helix-loop-helix transcription factor | Bowman | -6.202 |
| HORVU.MOREX.r2.2HG0177680 | chr2H:664509017-664512515 | HC | Receptor-like protein kinase-like protein | Bowman | -6.648 |
| HORVU.MOREX.r2.3HG0183620 | chr3H:5309287-5319127 | HC | Dipeptidyl peptidase family member 6 | Bowman | -7.913 |
| HORVU.MOREX.r2.3HG0190120 | chr3H:20699388-20703669 | HC | Protein mak16 | Bowman | -5.467 |
| HORVU.MOREX.r2.3HG0202100 | chr3H:90881395-90885357 | HC | Subtilisin-like protease | Bowman | -6.751 |
| HORVU.MOREX.r2.3HG0236200 | chr3H:466305149-466343488 | HC | Myosin | Bowman | -6.212 |
| HORVU.MOREX.r2.3HG0237020 | chr3H:471874878-471875578 | LC | Trehalose 6-phosphate phosphatase | Bowman | -7.816 |
| HORVU.MOREX.r2.3HG0261180 | chr3H:589771225-589772595 | HC | Kelch repeat-containing F-box protein-like | Bowman | -4.923 |
| HORVU.MOREX.r2.4HG0285410 | chr4H:39078042-39084297 | HC | 4-coumarate:CoA ligase-like protein | Bowman | -6.272 |
| HORVU.MOREX.r2.4HG0331810 | chr4H:558045719-558049589 | HC | Endo-1,4-beta-xylanase | Bowman | -4.871 |
| HORVU.MOREX.r2.4HG0339670 | chr4H:594444392-594450049 | HC | C2 calcium/lipid-binding and GRAM domain protein | Bowman | -7.738 |
| HORVU.MOREX.r2.5HG0364820 | chr5H:82881920-82886023 | HC | Diacylglycerol kinase | Bowman | -6.859 |
| HORVU.MOREX.r2.5HG0369790 | chr5H:131610292-131619750 | HC | Phenylalanine--tRNA ligase alpha subunit | Bowman | -7.065 |
| HORVU.MOREX.r2.5HG0377930 | chr5H:251560409-251561035 | LC | Retrotransposon protein, putative, unclassified | Bowman | -7.085 |
| HORVU.MOREX.r2.5HG0391800 | chr5H:393000003-393000218 | LC | Retrovirus-related Pol polyprotein from transposon TNT 1-94 | Bowman | -6.708 |
| HORVU.MOREX.r2.5HG0395820 | chr5H:420941243-420942231 | LC | Transposon protein, putative, Mutator sub-class | Bowman | -6.978 |
| HORVU.MOREX.r2.5HG0430150 | chr5H:553837631-553840367 | HC | BTB-POZ and MATH domain protein | Bowman | -4.574 |
| HORVU.MOREX.r2.5HG0444880 | chr5H:591669143-591672095 | HC | Receptor-kinase, putative | Bowman | -4.203 |
| HORVU.MOREX.r2.6HG0449000 | chr6H:3771116-3772750 | HC | Patatin | Bowman | -4.753 |
| HORVU.MOREX.r2.6HG0453960 | chr6H:15694935-15696614 | HC | DEAD box RNA helicase family protein | Bowman | -5.731 |
| HORVU.MOREX.r2.6HG0454420 | chr6H:16623225-16625997 | HC | Protein trichome birefringence | Bowman | -7.267 |
| HORVU.MOREX.r2.6HG0475700 | chr6H:155459812-155460372 | HC | Serine/arginine rich splicing factor, putative | Bowman | -7.257 |
| HORVU.MOREX.r2.6HG0494720 | chr6H:390439834-390440184 | LC | Transposase | Bowman | -6.691 |
| HORVU.MOREX.r2.6HG0508080 | chr6H:502106911-502108492 | HC | GDSL esterase/lipase | Bowman | -7.568 |
| HORVU.MOREX.r2.6HG0526460 | chr6H:572349455-572355288 | HC | Protein argonaute | Bowman | -4.332 |
| HORVU.MOREX.r2.7HG0568330 | chr7H:211071972-211073720 | HC | Bidirectional sugar transporter SWEET | Bowman | -6.519 |
| HORVU.MOREX.r2.7HG0581330 | chr7H:391968639-391980279 | LC | Monodehydroascorbate reductase | Bowman | -7.082 |
| HORVU.MOREX.r2.7HG0606620 | chr7H:584686166-584687107 | LC | LINE-1 reverse transcriptase-like protein | Bowman | -5.089 |
| HORVU.MOREX.r2.7HG0611930 | chr7H:603855609-603856931 | HC | Glutathione S-transferase T3 | Bowman | -5.539 |
| HORVU.MOREX.r2.7HG0614560 | chr7H:612276477-612281139 | LC | Retrotransposon protein, putative, Ty3-gypsy subclass, expressed | Bowman | -6.282 |
| HORVU.MOREX.r2.2HG0173110 | chr2H:653105226-653110931 | HC | Serine/threonine-protein kinase | BW230 | -9.929 |
| HORVU.MOREX.r2.6HG0488850 | chr6H:323675690-323676442 | LC | Transposon Ty3-G Gag-Pol polyprotein | BW230 | -9.833 |
| HORVU.MOREX.r2.1HG0012770 | chr1H:45731587-45732094 | HC | HAT family dimerisation domain containing protein | BW230 | -6.26 |
| HORVU.MOREX.r2.1HG0046240 | chr1H:390556109-390556444 | LC | Retrovirus-related Pol polyprotein LINE-1 | BW230 | -7.89 |
| HORVU.MOREX.r2.1HG0057920 | chr1H:460069043-460073381 | HC | S-adenosyl-L-methionine-dependent methyltransferases superfamily protein | BW230 | -4.395 |
| HORVU.MOREX.r2.1HG0068660 | chr1H:497757845-497760562 | HC | Phospholipase D | BW230 | -7.57 |
| HORVU.MOREX.r2.2HG0118280 | chr2H:241568738-241572087 | HC | WAT1-related protein | BW230 | -7.338 |
| HORVU.MOREX.r2.2HG0120020 | chr2H:269612514-269646141 | HC | DDT domain-containing protein | BW230 | -5.704 |
| HORVU.MOREX.r2.2HG0139090 | chr2H:485257003-485258641 | HC | Late embryogenesis abundant protein | BW230 | -4.187 |
| HORVU.MOREX.r2.4HG0275850 | chr4H:165432-168523 | HC | SPFH domain/band 7 family protein | BW230 | -4.882 |
| HORVU.MOREX.r2.4HG0315740 | chr4H:411782739-411788823 | HC | Apyrase-like protein | BW230 | -4.209 |
| HORVU.MOREX.r2.4HG0318290 | chr4H:441469583-441472462 | HC | Peptide chain release factor, putative | BW230 | -5.478 |
| HORVU.MOREX.r2.5HG0392150 | chr5H:395285559-395286527 | LC | Retrotransposon protein, putative, unclassified | BW230 | -7.15 |
| HORVU.MOREX.r2.5HG0435640 | chr5H:568834975-568837128 | LC | Retrotransposon protein, putative, Ty3-gypsy subclass | BW230 | -5.842 |
| HORVU.MOREX.r2.5HG0445270 | chr5H:592355515-592356855 | LC | LINE-1 reverse transcriptase-like protein | BW230 | -8.381 |
| HORVU.MOREX.r2.7HG0544650 | chr7H:46136592-46141009 | HC | Ribonucleoside-diphosphate reductase | BW230 | -4.651 |
| HORVU.MOREX.r2.7HG0583590 | chr7H:415577823-415581373 | HC | Amino acid transporter, putative | BW230 | -8.015 |
| HORVU.MOREX.r2.7HG0583780 | chr7H:418291692-418296667 | HC | Programmed cell death protein 4 | BW230 | -5.704 |
